# Therapeutic effects of mesenchymal stem cell-derived extracellular vesicles in osteoporosis models: a systematic review and meta-analysis of preclinical studies

**DOI:** 10.3389/fendo.2025.1625969

**Published:** 2025-09-16

**Authors:** Ying Zhang, Yi Liu, Shaoyun Wang, Chao Wang

**Affiliations:** ^1^ Department of Orthopedics, The Affiliated Hospital Southwest Medical University, Luzhou, Sichuan, China; ^2^ Trauma Center, The First Affiliated Hospital of Kunming Medical University, Kunming, Yunnan, China

**Keywords:** extracellular vesicle, osteoporosis, mesenchymal stem cell, bone mineral density, meta-analysis

## Abstract

**Objective:**

Traditional pharmacological treatments for osteoporosis face challenges due to various limitations, including long-term safety concerns and limited bone anabolic effects. Mesenchymal stem cell-derived extracellular vesicles (MSC-EVs) have emerged as a promising cell-free alternative therapy. However, their preclinical efficacy and the factors driving heterogeneity still require systematic evaluation.

**Methods:**

A systematic search was conducted in PubMed, EMBASE, Cochrane Library, and Web of Science (from inception to February 2025). Two independent authors performed literature screening, data extraction, and risk of bias assessment. A random-effects model was used to pool and analyze bone mineral density (BMD), bone volume fraction (BV/TV), trabecular/cortical structural parameters, and biomechanical test results. Publication bias was assessed using funnel plots and Egger’s test, while leave-one-out sensitivity analysis was performed to evaluate the stability of the results. Subgroup analyses were conducted based on animal type, EVs source, synthesis method, engineering approach, intervention route, frequency, and treatment duration.

**Results:**

A total of 17 studies were included. The results demonstrated that, compared to the control group, MSC-EVs significantly increased BMD, BV/TV, trabecular number (Tb.N), trabecular thickness (Tb.Th), cortical thickness (Ct.Th), mineral apposition rate (MAR), and the ultimate load-bearing capacity of the femur, while reducing trabecular separation (Tb.Sp). Significant heterogeneity and publication bias were observed in all analyses. Sensitivity analysis confirmed the robustness of all results.

**Conclusions:**

MSC-EVs demonstrate significant improvements in preclinical osteoporosis models, highlighting its potential for clinical translation. However, further standardized studies are needed to evaluate the long-term efficacy and safety of MSC-EVs.

## Introduction

Osteoporosis is a progressive systemic skeletal disease caused by an imbalance between bone formation and bone resorption, characterized by decreased bone mass and disruption of bone microstructure, which significantly increases the risk of fractures (1, 2). Globally, this disease affects approximately 200 million middle-aged and elderly individuals, with the risk of osteoporotic fractures increasing annually among those over 60 years old (3). Epidemiological data indicate that the annual cumulative number of osteoporotic fractures exceeds 8.9 million cases (4). The disease burden is particularly severe in older populations, with a prevalence of 77.1% in women over 80 years old and 46.3% in men of the same age group (5). Hip fractures, the most severe complication, result in approximately 20% of patients dying within one year after surgery, drawing widespread attention in the medical field (6, 7).

The current clinical treatment for osteoporosis primarily relies on bisphosphonate drugs, which inhibit bone resorption (8, 9). However, long-term use of these drugs may lead to severe adverse effects, such as osteonecrosis of the jaw and atypical femoral fractures (10). Although new anti-osteoporosis drugs, such as cathepsin K inhibitors and parathyroid hormone analogs, have been introduced in recent years, challenges remain, including high treatment costs, complex administration methods, and uncertain long-term efficacy (11, 12). These treatment limitations have driven researchers to explore novel, safe, and effective alternative therapies.

MSC-based cell therapy has garnered attention due to its regenerative and differentiation capabilities, demonstrating effectiveness in autoimmune diseases, graft-versus-host disease, and articular cartilage injuries (13, 14). The therapeutic potential of MSCs in osteoporosis relies on three mechanisms: migration and homing, induction of angiogenesis, and immunomodulation (15, 16). However, MSC-mediated cell therapy faces challenges, particularly in maintaining cell viability and efficacy throughout the treatment process (17). To address these limitations, extracellular vesicles secreted by mesenchymal stem cells (MSC-EVs) have emerged as a key mediator of paracrine effects and a research hotspot in regenerative medicine due to their unique nano-carrier properties. Compared to traditional stem cell transplantation, MSC-EVs can stably deliver functional miRNAs, cytokines, and signaling proteins while avoiding issues such as low cell survival rates, tumorigenic risks, and immune rejection (18, 19). In the field of osteoporosis treatment, Wang et al. (20) utilized “click chemistry” to conjugate MSC-EVs with alendronate, demonstrating high affinity for hydroxyapatite. This approach significantly promoted osteoblast differentiation *in vitro* and exhibited anti-osteoporotic effects and safety in osteoporotic mice. Another study found that miR-27a carried by MSC-EVs improved osteoporosis by inhibiting DKK2 expression, thereby activating the Wnt/β-catenin signaling pathway (21). Additionally, MSC-EVs can regulate vascular endothelial growth factor (VEGF) secretion to enhance local microvascular formation, which is crucial for providing nutritional support for bone regeneration (22, 23).

Since 2020, preclinical studies on MSC-EVs for osteoporosis treatment have increased; however, a comprehensive and up-to-date meta-analysis on their efficacy remains lacking, which is crucial for clinical translation. Notably, existing studies exhibit significant heterogeneity in EV preparation methods (such as isolation techniques and engineering strategies), administration protocols (including dosage, frequency, and delivery routes), and osteoporosis modeling approaches (such as ovariectomy-induced and drug-induced models). These variations may influence the analytical outcomes. Therefore, in addition to evaluating the potential benefits of MSC-EVs in improving osteoporosis models, we conducted a subgroup analysis to explore the impact of these influencing factors on therapeutic efficacy. This meta-analysis aims to provide evidence supporting the clinical translation of MSC-EVs for osteoporosis treatment.

## Materials and methods

### Systematic review

This study was conducted in accordance with the guidelines of the Preferred Reporting Items for Systematic Reviews and Meta-Analyses (PRISMA) checklist (24). The present study was registered in the International Prospective Register of Systematic Reviews (PROSPERO, https://www.crd.york.ac.uk/prospero/, CRD420251047216).

### Search strategy

Two researchers independently searched four major databases, including PubMed, EMBASE, Web of Science, and Cochrane Library, from their inception to January 1, 2025. The search strategy combined Medical Subject Headings (MeSH) and free-text terms, focusing on intervention-related terms (e.g., mesenchymal stem cell-derived extracellular vesicles, exosomes, or microvesicles) and disease models (e.g., animal osteoporosis or bone loss). The detailed search strategy is provided in **Supplementary Table 1**. Discrepancies in search results were resolved through discussion with a third researcher. Additionally, the references of studies meeting the inclusion criteria were reviewed to identify potentially relevant studies.

### Inclusion and exclusion criteria

#### Inclusion criteria

1. Controlled study design with MSC-EVs intervention in the experimental group, with no restrictions on engineering details or intervention methods;
2. Any osteoporosis animal model, including rats and mice, with no restrictions on induction methods (e.g., ovariectomy-induced osteoporosis models);
3. Studies including a control group receiving placebo or no treatment;
4. Reporting at least one bone-related quantitative outcome, such as bone mineral density (BMD), bone volume/total volume (BV/TV), trabecular number (Tb.N), trabecular separation (Tb.Sp), trabecular thickness (Tb.Th).

#### Exclusion criteria

1. Non-controlled studies or studies with combined interventions (e.g., EVs co-administered with drugs);
2. Reviews, meta-analyses, conference abstracts, or commentaries lacking original data;
3. Non-osteoporosis models (e.g., fracture healing or bone tumor models);
4. Studies not published in English;
5. Studies with unavailable or unextractable data.

### Study selection

Initially, all retrieved records were compiled, and duplicate entries were automatically removed using EndNote X20. Subsequently, preliminary screening was conducted based on titles and abstracts to exclude irrelevant studies. Finally, full-text articles were reviewed according to the inclusion and exclusion criteria to identify eligible studies for meta-analysis. The screening process was independently performed by two researchers, and discrepancies were resolved through discussion with a third researcher. The selection process strictly followed the PRISMA flowchart, with detailed documentation of the number of excluded studies and reasons at each stage.

### Data extraction

Two researchers independently extracted data using a standardized Excel template, including: (1) study characteristics (author, year, animal species, gender, weight, modeling method); (2) EVs properties (source, engineering method); (3) intervention protocols (frequency, route, duration); (4) outcome data (BMD, BV/TV, Tb.N, Tb.Sp, Tb.Th, Ct.Th). Graphical data were extracted using Origin software (2021 version), and quantitative data were presented as mean ± standard deviation (mean ± SD). Discrepancies in data extraction were resolved through discussion with a third researcher. For data not directly available, attempts were made to contact the corresponding authors for further information.

### Primary and secondary outcomes

Primary outcomes were obtained through microCT analysis, including BMD, BV/TV, and trabecular bone structural parameters (Tb.Th, Tb.N, and Tb.Sp). Secondary outcomes primarily included Ct.Th, mineral apposition rate (MAR, observed through double fluorescent labeling), and the ultimate load-bearing capacity of the femur (determined by three-point bending test). All parameters were reported as mean ± standard deviation (mean ± SD).

### Risk of bias assessment

The methodological quality of animal studies was assessed using the Systematic Review Centre for Laboratory Animal Experimentation (SYRCLE) risk of bias tool. This tool includes 10 criteria: sequence generation, allocation concealment, baseline characteristics, random housing, blinding of participants, random outcome assessment, blinding of outcome detection, incomplete data, selective reporting, and other biases. Two reviewers independently scored each study based on the criteria, with results categorized as “low risk,” “high risk,” or “unclear risk.” Discrepancies in assessment results were resolved through discussion with a third reviewer. The summarized results were visualized using Review Manager (RevMan) 5.3.

### Statistical analysis

Due to methodological heterogeneity, a random-effects model was applied for the meta-analysis of continuous data, with results presented as standardized mean differences (SMDs) with 95% confidence intervals (CIs). Heterogeneity was evaluated using the *I*² statistic, where *I*² ≥ 50% indicated significant heterogeneity. Subgroup analyses were conducted when at least 10 studies reported the relevant indicators, based on predefined categories, including animal species, age, EV source, size, isolation method, purification method, intervention route, dose, frequency, and duration. Sensitivity analysis was performed to assess the robustness of the pooled results. Publication bias was evaluated using funnel plots and Egger’s regression test. A *P*-value < 0.05 was considered statistically significant. All analyses were conducted using RevMan 5.3 and Stata SE 16.0 software.

## Results

### Literature selection

**Figure 1:**
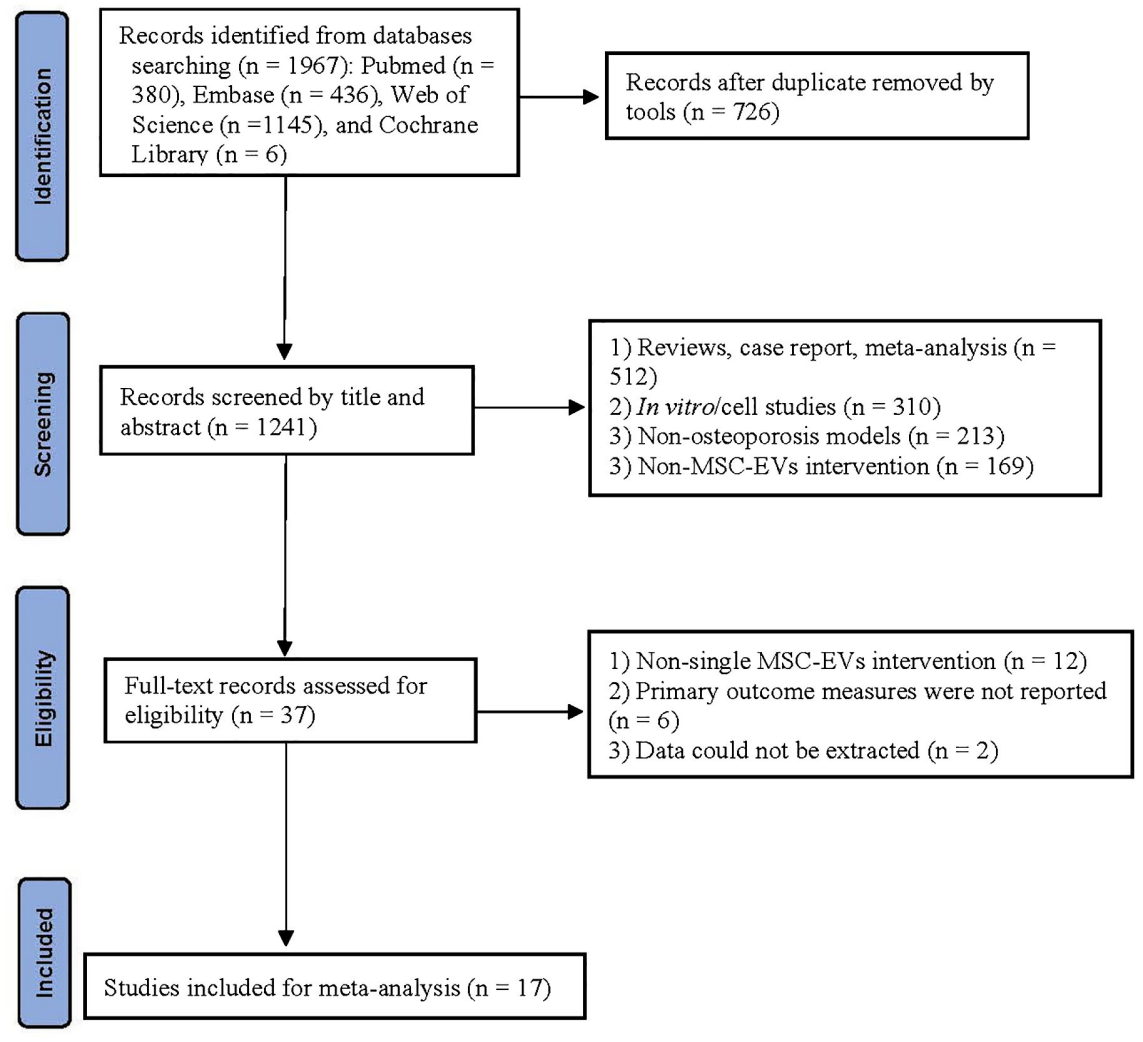
Flowchart of study selection.

A total of 1,967 records were identified through database searches: PubMed (380), Embase (436), Web of Science (1,145), and Cochrane Library (6). After removing 726 duplicates, 1,241 articles underwent title/abstract screening. Exclusions at this stage included reviews/case reports (512), *in vitro* studies (310), non-osteoporosis models (213), and non-MSC-EV interventions (169). Subsequently, full-text assessment of 37 articles led to the exclusion of 20 studies, with 17 studies (20, 21, 25–39) meeting the inclusion criteria. The literature selection process is detailed in the PRISMA flowchart (**Figure 1**).

### Study characteristics

Between 2016 and 2024, all 17 studies were conducted in China. Notably, 16 studies were published in 2020 or later, indicating increasing attention to MSC-EVs in osteoporosis treatment in recent years. The animal models primarily used female animals (15/17 studies), with Sprague-Dawley rats (n = 6) and C57BL/6 mice (n = 9) as the main species. Except for one study using a hindlimb unloading (HU)-induced osteoporosis model, the remaining studies (n = 15) employed ovariectomy-induced osteoporosis models. **Table 1** details the main characteristics of the animal models.

**Table 1:** Characteristics of animal models in the included studies.

Author	Year	Country	Specie	Gender	Age	Weight	Total number	Model of osteoporosis
Ge et al (25)	2021	China	C57BL/6 mice	Female	10-week-old	Not description	40	Ovariectomized
Gui et al (26)	2024	China	C57BL/6 mice	Female	8-week-old	Not description	50	Ovariectomized
Hu et al (27)	2020	China	C57BL/6 mice	Female	8-week-old	Not description	30	Ovariectomized
Huang et al (28)	2021	China	Sprague Dawley (SD) rats	Female	10-week-old	230 - 250 g	40	Ovariectomized
Li et al (29)	2021	China	Sprague Dawley (SD) rats	Female	8-week-old	294 ± 11 g	40	Ovariectomized
Li et al (30)	2023	China	Not description	Female	6-week-old	Not description	24	Ovariectomized
Li et al (31)	2024	China	C57BL/6J mice	Female	2-month-old	Not description	20	Ovariectomized
Lu et al (32)	2020	China	C57BL/6J mice	Male	3-month-old	Not description	15	Not description
Lu et al (33)	2021	China	BALB/c mice	Female	8-week-old	25-30g	30	Ovariectomized
Qi et al (34)	2016	China	Sprague Dawley (SD) rats	Female	12 weeks old	250-300 g	60	Ovariectomized
Qi et al (35)	2023	China	Sprague–Dawley (SD) rats	Female	10 weeks old	230–250 g	18	Ovariectomized
Qiu et al (36)	2020	China	Sprague Dawley (SD) rats	Female	12 weeks old	280-300 g	66	Ovariectomized
Wang et al (20)	2020	China	Sprague Dawley (SD) rats	Female	6-month-old	300–350 g	50	Ovariectomized
Wang et al (21)	2022	China	C57BL/6J mice	Female	12 weeks old	28–30 g	40	Ovariectomized
Wang et al (37)	2023	China	C57BL/6 mice	Female	Not description	Not description	42	Ovariectomized
Xiao et al (38)	2021	China	C57BL/6J mice	Male	6-month-old	Not description	20	Osteoporosis caused by mechanical unloading
Yang et al (39)	2022	China	C57BL/6J mice	Female	8-week-old	Not description	40	Ovariectomized

Additionally, **Table 2** presents the characteristics of EVs and intervention details in the included studies. Specifically, EVs were primarily derived from bone marrow mesenchymal stem cells (BMSCs, n = 10) and human umbilical cord mesenchymal stem cells (n = 2). The diameter of EVs, reported in 14 studies, ranged from 30 to 5000 nm. The most common methods for isolating and purifying EVs are ultracentrifugation (n = 14) and filtration (n = 11), respectively. Regarding MSC-EVs intervention details, 12 studies administered EVs via intravenous injection, 2 studies via intraperitoneal injection, 1 study via scaffold loading, and 1 study via femoral periosteal injection. Injection frequencies included once a week (n = 8), twice a week (n = 5), thrice a week (n = 1), every 3 days (n = 1), once a day (n = 1), and once (n = 1). Treatment durations included 1 week (n = 1), 2 weeks (n = 1), 4 weeks (n = 5), 6 weeks (n = 1), 2 months (n = 7), and 3 months (n = 2).

**Table 2:** Characteristics and therapeutic method of EVs.

Author	Year	Characteristics of EV									Therapeutic methods			
		Isolation and purification				EV characterization								
		Source	Cell culture	Isolation	Purification	TEM	Particle concentration	Protein concentration	Marker	Diameter (nm)	Route of administration	Dose of administration	Time of administration	Duration
Ge et al (25)	2021	hUC-MSC	Cultivate to P3	Ultracentrifugation	Filtered through a 0.22 µm sterile filter membrane	Sphere-like morphology	Not description	Not description	CD9, CD63 , and TSG101	20-200 μm	Intraperitoneally	0.5 mg/kg	Every 3 days	6 weeks
Gui et al (26)	2024	BMSCs	BMSCs were treated with staurosporine (0.5 µM) for 6h	Ultracentrifugation	Not description	Cup-shaped morphology	Not description	Not description	PKH67	220-396 nm	Intravenously	10 mg/kg	Once a week	4 weeks
Hu et al (27)	2020	hUC-MSC	Cultivate to P2-P6	Ultracentrifugation	Filtered through a 0.22 μm filter	Cup- or sphere-like morphology	Not description	Not description	CD9, CD63, CD81, and TSG101	60 nm-150 nm	Intravenously	100 μg/100 μL PBS	Once a week	3 months
Huang et al (28)	2021	BMSCs	Cultivate to P2-P4	Ultracentrifugation	Filtered through a 0.22 μm filter	Cup- or sphere-like morphology	Not description	100 μg/ml	CD9, CD63, and CD81	40-120 nm)	Intravenously	100 μg	Once a week	2 months
Li et al (29)	2021	hBMSCs	Cultivate	Polymer precipitation kits	Not description	Not description	Not description	Not description	Alixs, CD63, and CD81	100-150nm	Intravenously	100 μL	Once a week	1 month
Li et al (30)	2023	BMSCs	Cultivate	Not description	Not description	Not description	Not description	Not description	Not description	Not description	Intravenously	Not description	Once a week	4 weeks
Li et al (31)	2024	MSCs	STS inducing apoptosis	Ultracentrifugation	Suspended in ice-cold	Round shape	Not description	4.6×10^9^ particles/mL	Annexin V, Histone 3, Cleaved-caspase 3, and CD63	50-5000 nm	Intravenously	100 μg	Once a week	2 months
Lu et al (32)	2020	BMSCs	Cultivate	Ultracentrifugation	Filtered through a 0.22 μm filter	Round shape	1-2 × 10^10^ particles/Ml	Not description	Syntenin 1, and TSG101	30-150nm	Not description	100 μg	Twice a week	2 months
Lu et al (33)	2021	Wharton’s jelly-MSCs	Cultivate	Ultracentrifugation	Filtered through a 0.22 μm filter	Round shape	Not description	Not description	CD9, CD63, and HSP70	185 nm	Intravenously	200 μg	Once a week	2 months
Qi et al (34)	2016	MSCs	Cultivate to 80-90%	Ultracentrifugation	Filtered through a 0.22 μm filter	Not description	Not description	Not description	CD9, CD63, and CD81	50-150 nm	Scaffold loading	200 µg	Once	2 months
Qi et al (35)	2023	BMSCs	Cultivate to P3	Ultracentrifugation	Filtered through a 0.22 μm filter	Hollow spherical microvesicles	Not description	Not description	CD63, CD81, and TSG101	50-120 nm	Intravenously	100 μg	Once a week	2 months
Qiu et al (36)	2020	BMSCs	Cultivate to P3	ExoEasy Maxi Kit	Filtered through a 0.45 μm filter	Low-density electrons in the vesicles	Not description	Not description	CD63 and CD9	30-100 nm.	Intravenously	100 μg	Once a day	2 weeks
Wang et al (20)	2020	mMSCs	Cultivate	Ultracentrifugation	Not description	Round shape	Not description	Not description	Not description	Not description	Intravenously	750 μg	Twice a week	2 months
Wang et al (21)	2022	MSCs	Cultivate	Ultracentrifugation	Filtered through a 0.22 μm filter	Round shape	Not description	Not description	CD63 and CD9	40-100 nm	Injected through periosteum of the femur	20 μL	Twice a week	1 week
Wang et al (37)	2023	BMSCs	Cultivate	Ultracentrifugation	Filtered through a 0.22 μm filter	Cup-shaped morphology	Not description	Not description	CD9, CD63, and CD81	100 nm	Intravenously	100 μg	Twice a week	3 months
Xiao et al (38)	2021	BMSCs	Cultivate to 80-90%	Ultracentrifugation	Filtered through a 0.22 μm filter	Round shape	Not description	Not description	CD63 and TSG101	40-260 nm	Intravenously	100 μL	Twice a week	4 weeks
Yang et al (39)	2022	BMSCs	Cultivate to 50-60%	Ultracentrifugation	The pellet was washed with PBS	Cup-shaped morphology	Not description	Not description	Alix, CD63, TSG101, and CD81	500nm	Intraperitoneally	100 μg	Thrice a week	4 weeks

BMSC, Bone marrow mesenchymal stem cell; hUC-MSC, Human umbilical cord mesenchymal stromal cell; hBMSC, Human bone marrow mesenchymal stem cell; MSC, Mesenchymal stem cell; mMSC, Mouse mesenchymal stem cell; STS, Staurosporine.

### Risk of bias assessment

**Figure 2:**
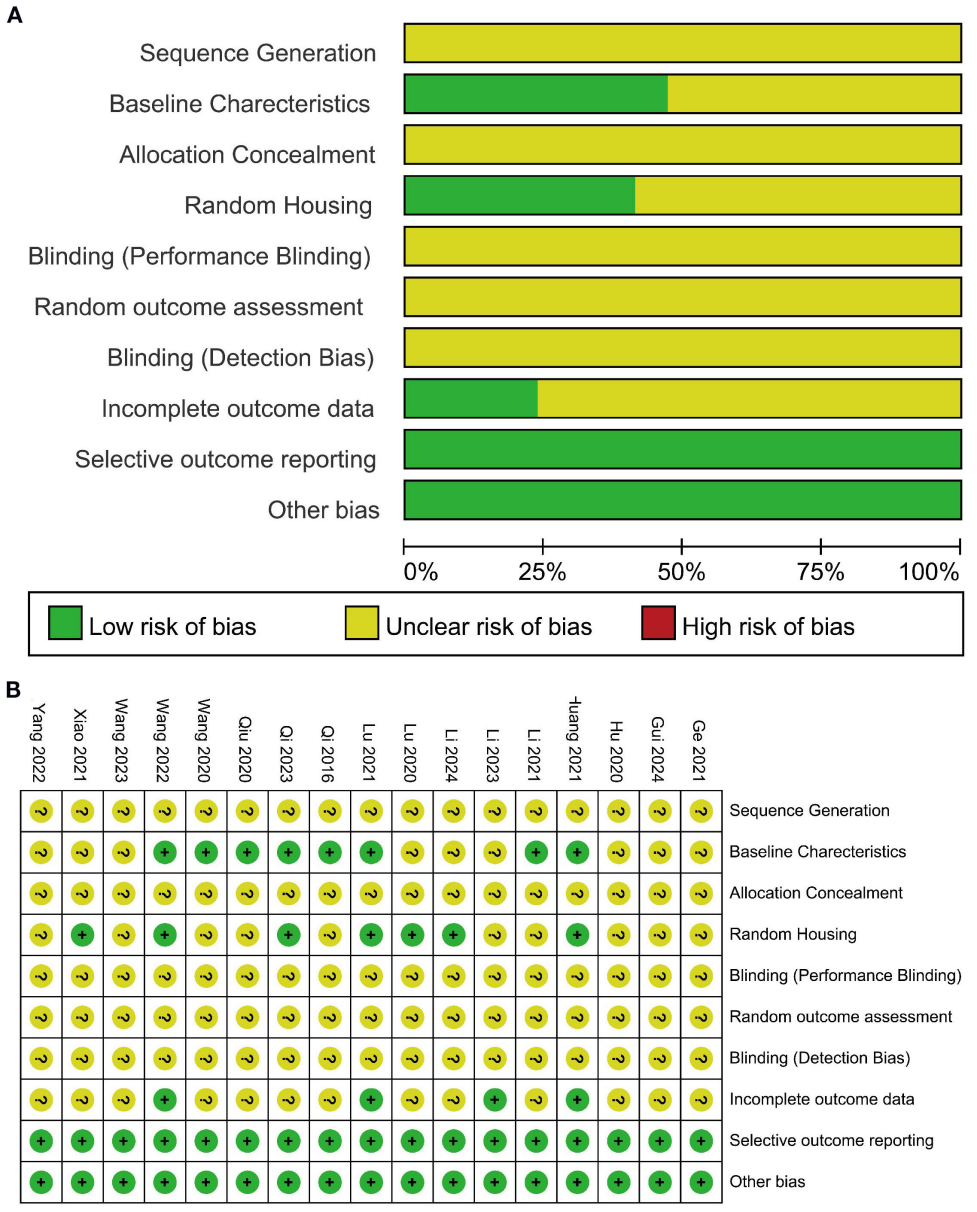
Risk of bias assessment results for 17 studies based on SYRCLE’s ROB tool. **(A)** Risk of bias graph; **(B)** Risk of bias summary.

The included studies did not clearly specify whether sequence generation methods were used for animal grouping, nor did they provide detailed descriptions of allocation concealment. Unclear risks of bias were identified in the areas of blinding of participants, blinding of outcome assessment, and randomization of outcome evaluation. Eight studies reported baseline characteristics of the included animals in detail, and seven studies described random housing of animals, which were considered to have a low risk of bias. Additionally, the included studies exhibited a low risk of bias in selective reporting. Overall, the included studies generally had unclear risks of bias, though some studies showed low risks of bias in specific domains. The detailed results of the risk of bias assessment are presented in **Figures 2A, B**.

### Meta-analysis results

#### MSC-EVs intervention significantly increases bone mineral density and bone volume in osteoporosis models

**Figure 3:**
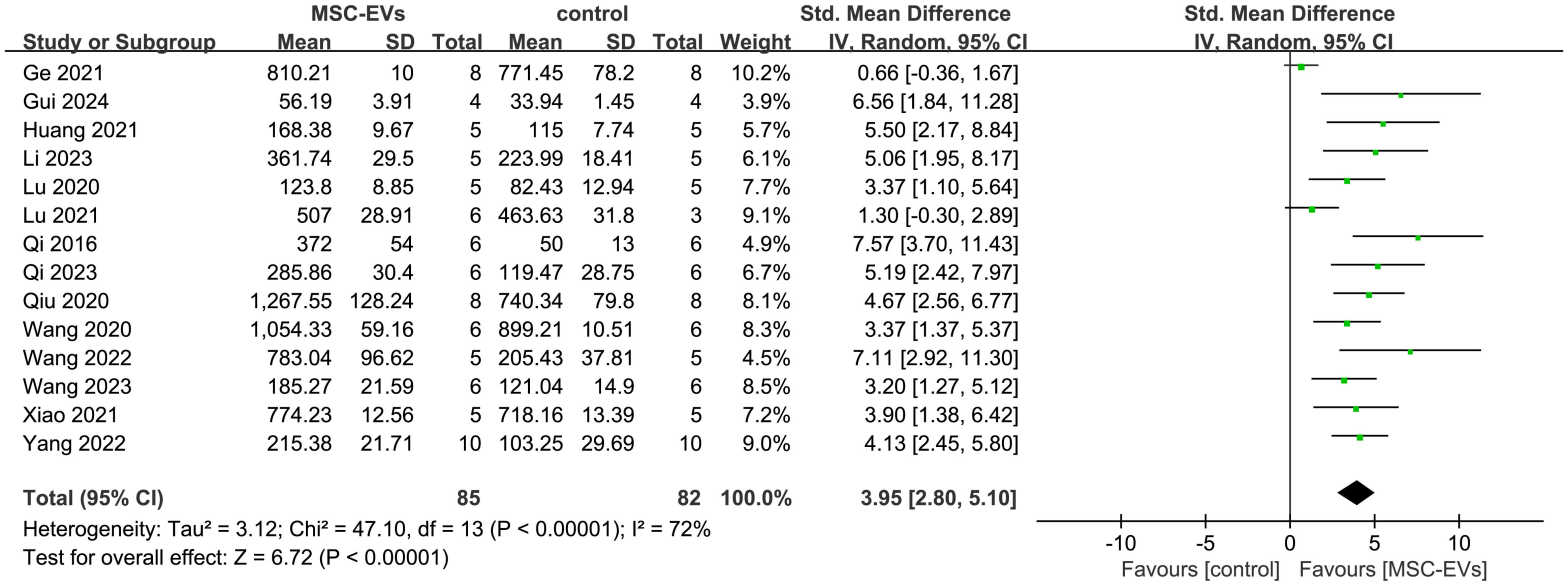
Forest plot showing the effect of MSC-EVs on BMD in osteoporosis models. Data are presented as standardized mean differences (SMD) with 95% confidence intervals (CI).

Fourteen studies reported the effects of MSC-EVs on BMD in osteoporosis models. Meta-analysis results showed that MSC-EVs intervention significantly increased BMD in animal models (SMD = 3.95; 95% CI: 2.80 to 5.10; *P* < 0.00001) (**Figure 3**). Due to significant heterogeneity (*I*² = 72%, *P* < 0.00001), subgroup analyses were further conducted. Subgroups were categorized based on stem cell source (BMSC or non-BMSC), animal ages (immature or adult), gender (male or female), isolation method (ultracentrifugation), purification technique (filtered through a filter), EV size (small or large EVs), intervention routes (intravenous injection), frequency (once or twice a week), dose (≤ 100 μl/μg or > 100 μl/μg) and duration (<2 months or ≥2 months). Results indicated that all subgroups significantly increased BMD in osteoporosis models, but none were significant sources of heterogeneity (**Supplementary Figures 1–10**).

**Figure 4:**
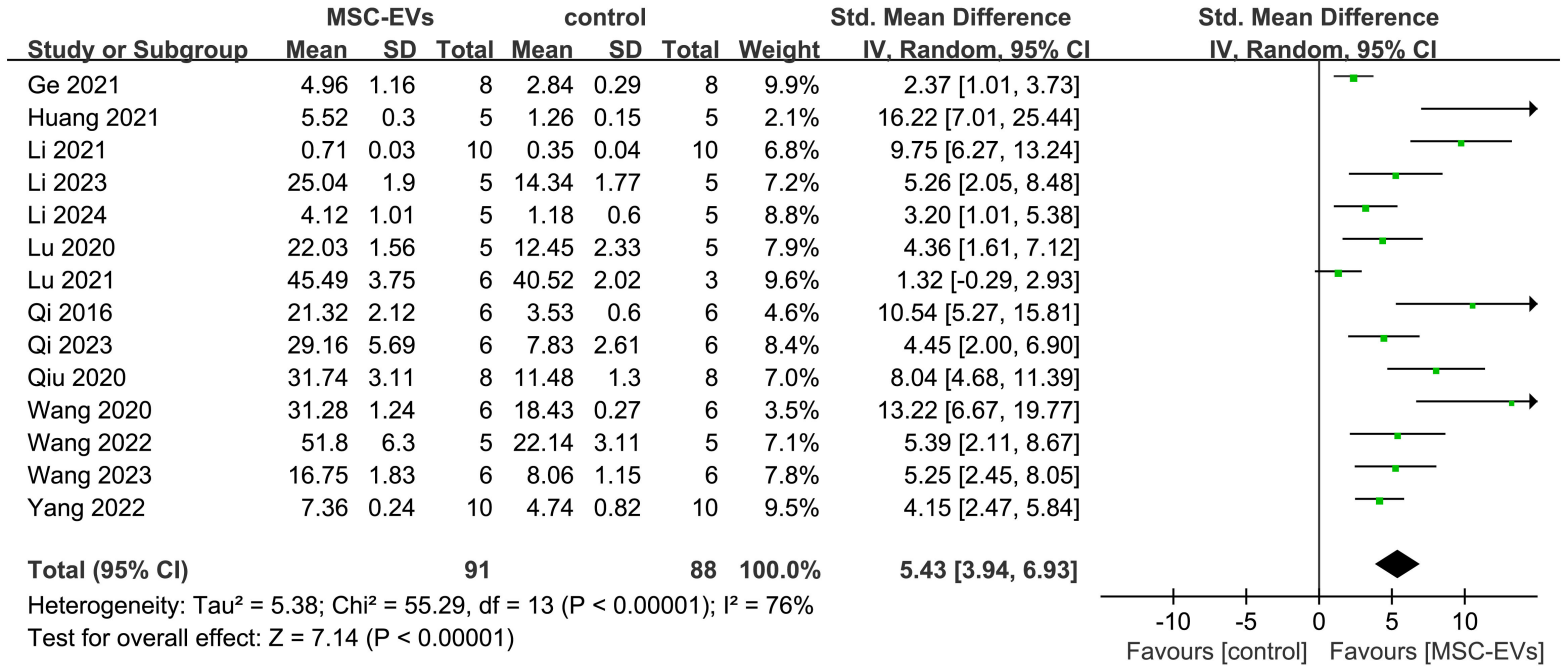
Forest plot depicting the effect of MSC-EVs on BV/TV in osteoporosis models. Data are presented as standardized mean differences (SMD) with 95% confidence intervals (CI).

Additionally, to explore the effects of MSC-EVs on bone volume in osteoporosis models, 14 studies reporting BV/TV were pooled. Results showed that MSC-EVs intervention significantly increased bone volume compared to the control group (SMD = 5.43; 95% CI: 3.94 to 6.93; *P* < 0.00001; *I*² = 76%, *P* < 0.00001) (**Figure 4**). Further subgroup analyses revealed that, except for the “> 100 μl/μg” subgroup, all other subgroups improved bone volume in osteoporosis models. However, none were significant sources of heterogeneity (**Supplementary Figures 11–19**). These results demonstrate that MSC-EVs intervention significantly increases BMD and bone volume in models, thereby ameliorating osteoporosis-induced bone loss.

#### MSC-EVs intervention significantly improves bone structural parameters in osteoporosis models

**Figure 5:**
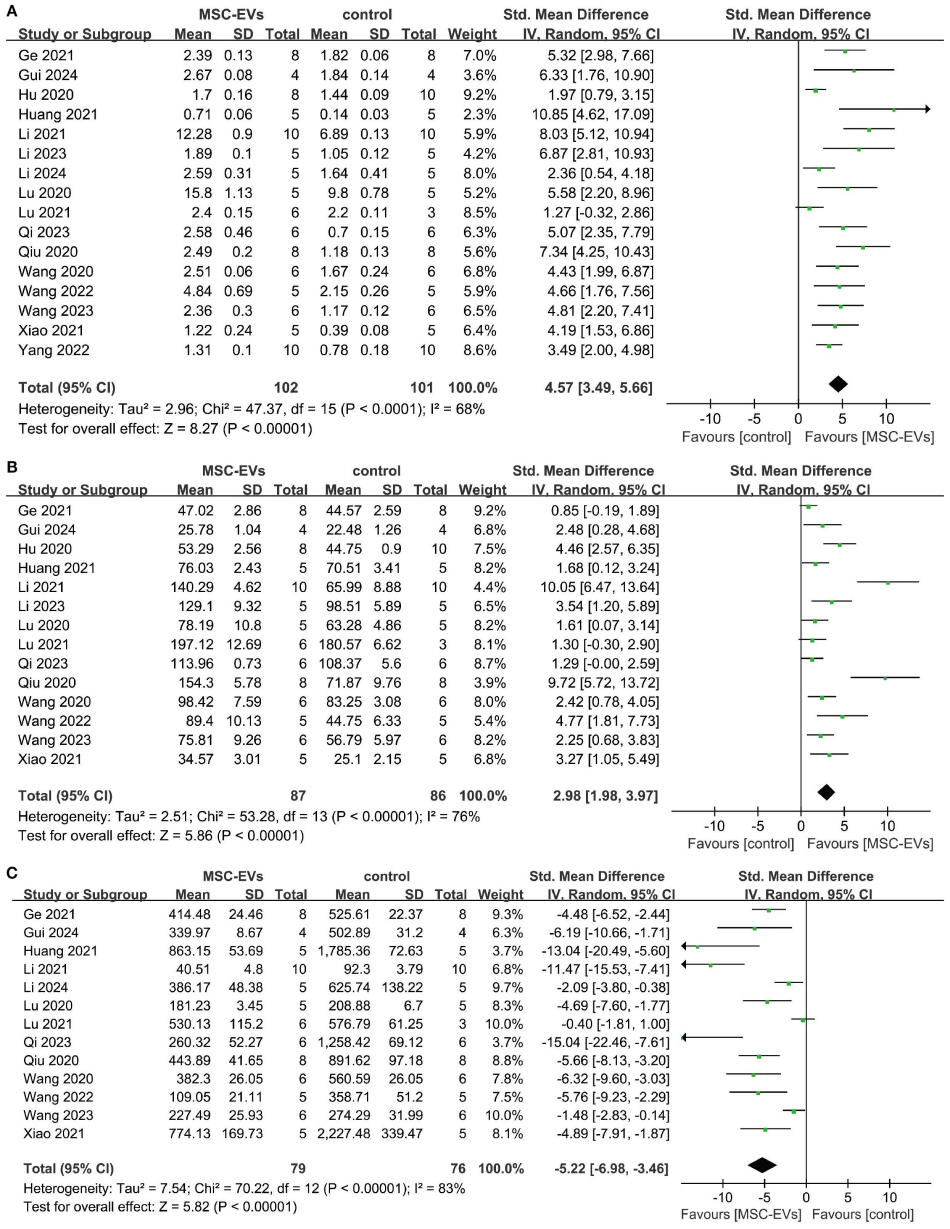
Forest plot showing the effect of MSC-EVs on trabecular structural parameters in osteoporosis models. **(A)** trabecular number (Tb. N); **(B)** trabecular thickness (Tb. Th); **(C)** trabecular separation/marrow thickness (Tb. Sp). Data are presented as standardized mean differences (SMD) with 95% confidence intervals (CI).

Trabecular bone structural parameters (Tb.N, Tb.Th, and Tb.Sp) are key indicators for assessing the spatial morphology of trabecular bone. Sixteen studies reported Tb.N parameters before and after MSC-EVs intervention. Meta-analysis results showed that MSC-EVs intervention significantly increased Tb.N in animal models (SMD = 4.57; 95% CI: 3.49 to 5.66; *P* < 0.00001; *I*² = 68%, *P* < 0.0001) (**Figure 5A**). Subgroup analyses revealed that, except for the “> 100 μl/μg” subgroup in intervention dose, all other subgroups significantly increased Tb.N in osteoporosis models, but none were significant sources of heterogeneity (**Supplementary Figures 20–29**). Pooled analysis of 14 studies showed that MSC-EVs intervention significantly increased Tb.Th in animal models (SMD = 2.98; 95% CI: 1.98 to 3.97; *P* < 0.00001) (**Figure 5B**). Due to significant heterogeneity (*I*² = 76%, *P* < 0.00001), further subgroup analyses indicated that, except for the “other frequencies” and “other routes” subgroups, all subgroups significantly increased Tb.Th in osteoporosis models, but none were significant sources of heterogeneity (**Supplementary Figures 30–39**). Next, pooled analysis of 13 studies on Tb.Sp before and after MSC-EVs intervention showed that MSC-EVs intervention significantly reduced Tb.Sp in osteoporosis models (SMD = -5.22; 95% CI: -6.98 to -3.46; *P* < 0.00001; *I*² = 83%, *P* < 0.0001) (**Figure 5C**). Further subgroup analyses revealed that, except for the “> 100 μl/μg” subgroup in intervention dose, all other subgroups reduced Tb.Sp (**Supplementary Figures 40–49**). However, none of the subgroups were significant sources of heterogeneity.

**Figure 6:**
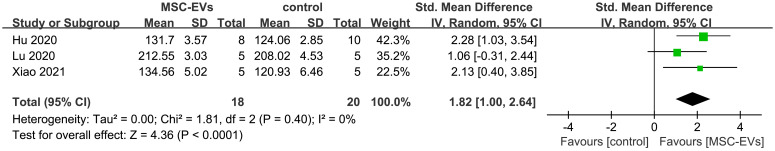
Forest plot depicting the effect of MSC-EVs on Ct.Th in osteoporosis models. Data are presented as standardized mean differences (SMD) with 95% confidence intervals (CI).

**Figure 7:**
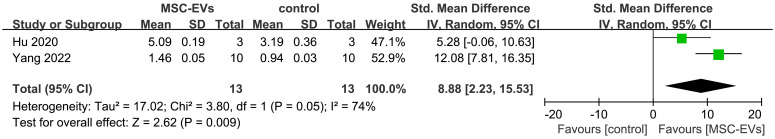
Forest plot showing the effect of MSC-EVs on mineral apposition rate (MAR) in the osteoporosis model. Data are presented as standardized mean differences (SMD) with 95% confidence intervals (CI).

Additionally, three studies reported Ct.Th in the models. Pooled analysis showed that MSC-EVs intervention significantly increased Ct.Th (SMD = 1.82; 95% CI: 1.00 to 2.64; *P* < 0.0001; *I*² = 0%, *P* = 0.40) (**Figure 6**). Two studies reported the bone remodeling parameter mineral apposition rate (MAR). Meta-analysis showed that MSC-EVs intervention accelerated bone mineralization, possibly indicating increased osteoblast activity (SMD = 8.88; 95% CI: 2.23 to 15.53; *P* = 0.009; *I*² = 74%, *P* = 0.05) (**Figure 7**). Overall, compared to the control group, MSC-EVs intervention significantly improved trabecular and cortical bone structural parameters in osteoporosis models and promoted bone mineralization.

#### Ultimate load-bearing capacity of femur

**Figure 8:**
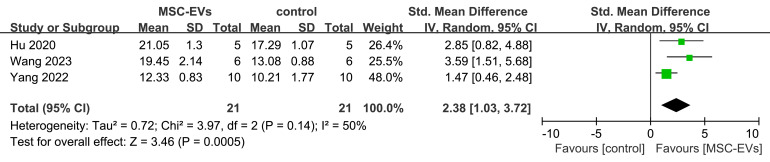
Forest plot depicting the effect of MSC-EVs on ultimate load-bearing capacity of the femur in osteoporosis model. Data are presented as standardized mean differences (SMD) with 95% confidence intervals (CI).

Three studies also evaluated the biomechanical properties of the femur in animal models before and after MSC-EVs intervention. Meta-analysis results showed that MSC-EVs intervention significantly increased the ultimate load-bearing capacity of the femur (SMD = 2.38; 95% CI: 1.03 to 3.72; P = 0.0005; *I*² = 50%, *P* = 0.14) (**Figure 8**).

### Sensitivity analysis and publication bias

**Figure 9:**
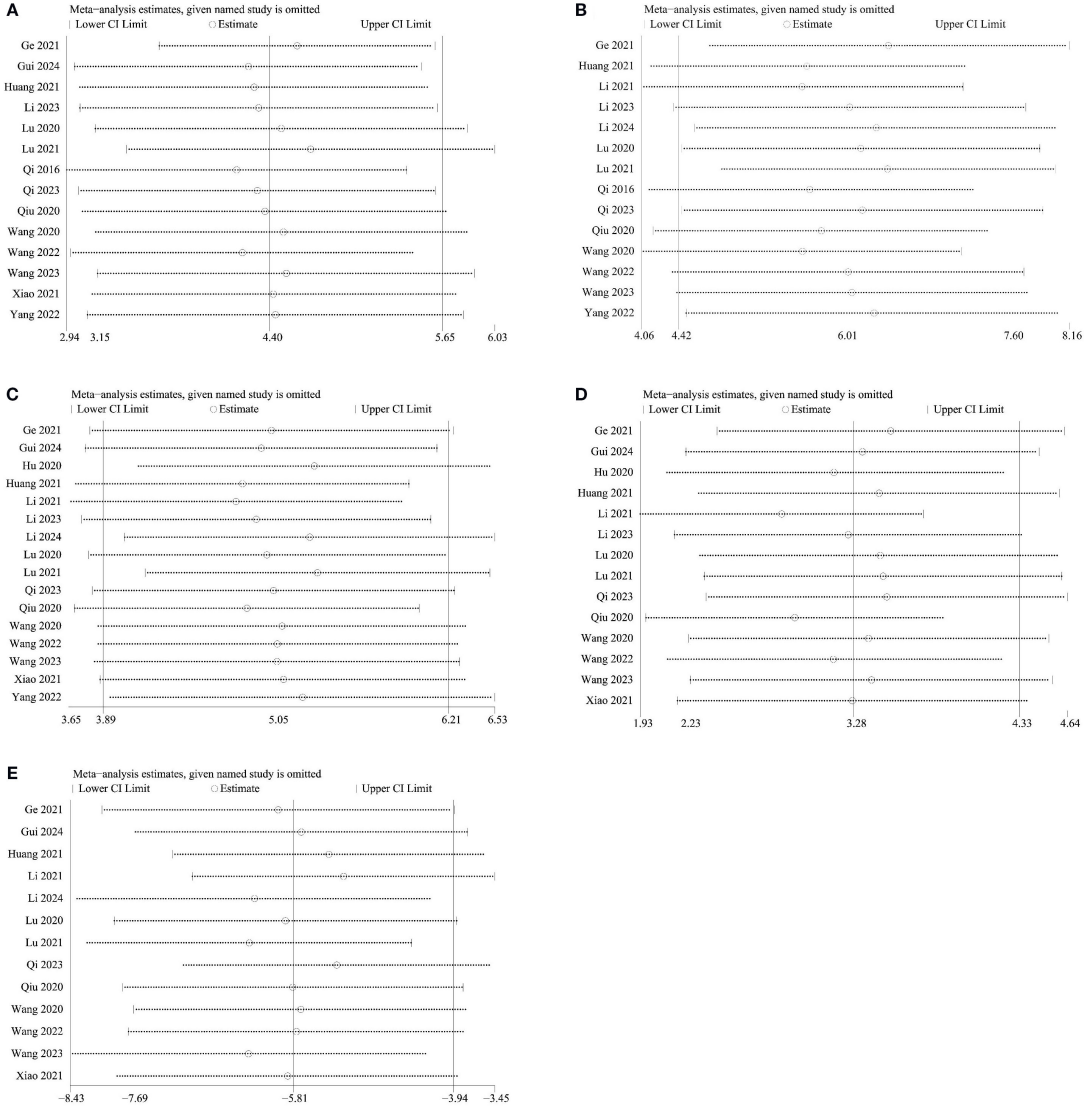
Sensitivity analysis results for primary outcomes. **(A)** BMD; **(B)** BV/TV; **(C)** Tb. N; **(D)** Tb. Th; **(E)** Tb. Sp.

To evaluate the robustness of the results, sensitivity analyses were conducted for BMD, BV/TV, Tb.N, Tb.Th, and Tb.Sp. Results showed that the outcomes remained consistent after excluding each individual study (**Figures 9A–E**), demonstrating the reliability and stability of the results. Further assessment of publication bias revealed asymmetry in the funnel plots, indicating the presence of publication bias (**Supplementary Figure 50**), which was confirmed by Egger’s test (**Table 3**). Trim-and-fill analysis for BMD, BV/TV, Tb.N, Tb.Th, and Tb.Sp showed no significant changes in heterogeneity, suggesting robust outcomes (**Supplementary Figure 51**).

**Table 3:** Publication bias analysis.

Outcome	SMD	95% CI	Egger's test (*p* value)	*t* value	Pooling model
SMD	2	[2.80, 5.10]	0.000	8.23	Random
BV/TV	5.43	[3.94, 6.93]	0.000	8.19	Random
Tb.N	4.57	[3.49, 5.66]	0.000	7.30	Random
Tb.Th	2.98	[1.98, 3.97]	0.000	9.06	Random
Tb.Sp	-5.22	[-6.98, -3.46]	0.000	-8.52	Random

## Discussion

To our knowledge, this is the first meta-analysis assessing the preclinical therapeutic efficacy of MSC-EVs for osteoporosis, providing a certain degree of reference value for further mechanistic exploration and clinical translation. This meta-analysis included 17 preclinical studies involving 625 animals. The pooled analysis results indicated that MSC-EVs intervention improved BMD, bone mass, structural parameters, bone remodeling parameters (MAR), and bone biomechanical properties in osteoporosis. Specifically, it increased BMD, BV/TV, Tb.N, Tb.Th, Ct.Th, MAR, and the ultimate load-bearing capacity of the femur while reducing Tb.Sp. These improvements suggest that MSC-EVs may contribute to the overall structural repair of osteoporotic bone, demonstrating promising potential for osteoporosis treatment in animal models. However, considering the limitations of study heterogeneity and the number of studies, further research is still needed to support the beneficial effects of MSC-EVs in osteoporosis models.

BMD, as an indicator of bone strength, is a key factor in the clinical diagnosis and treatment of osteoporosis as well as in the assessment of fracture risk (40). Specifically, an increase in BMD indicates that bone formation exceeds bone loss, resulting in increased bone mass. BV/TV represents the ratio of bone volume to tissue volume, directly reflecting changes in bone mass and playing a crucial role in evaluating the efficacy of osteoporosis treatments (41, 42). Among the included studies, 14 reported pre- and post-intervention measurements of BMD and BV/TV, highlighting their potential reference value and clinical significance. Based on the meta-analysis results, MSC-EVs increased BMD levels in the osteoporosis model compared to the control group, demonstrating a beneficial effect on bone strength and bone mass. However, given the significant heterogeneity observed in the pooled results for both indicators, these findings should be interpreted with caution. Although subgroup analysis showed that improvements in BMD and BV/TV were observed across various subgroups classified by EVs source, engineering methods, targets, intervention pathways, frequency, duration, and animal model types, none of these factors were identified as significant contributors to the observed heterogeneity.

Additionally, trabecular and cortical bone structural parameters are equally important for evaluating the therapeutic effects of osteoporosis treatment (43). Trabecular bone forms a porous lattice structure through interconnections and is arranged according to stress distribution patterns, which helps enhance the mechanical strength of bone tissue (44). As key indicators of trabecular spatial morphology, Tb.N, Tb.Th, and Tb.Sp were analyzed in this meta-analysis. The results showed that, compared to the control group, MSC-EVs treatment increased Tb.N and Tb.Th while reducing Tb.Sp, indicating that bone formation exceeded bone resorption, leading to significant structural improvements in the osteoporotic model. Compared to trabecular parameters, fewer studies have measured cortical bone parameters, as cortical bone changes often occur later than trabecular bone alterations. Among the included studies, three reported Ct.Th measurements, showing that MSC-EVs increased Ct.Th, which may suggest that MSC-EVs also hold considerable therapeutic potential in the later stages of bone formation. However, further studies with longer treatment durations are necessary to validate these findings.

**Figure 10:**
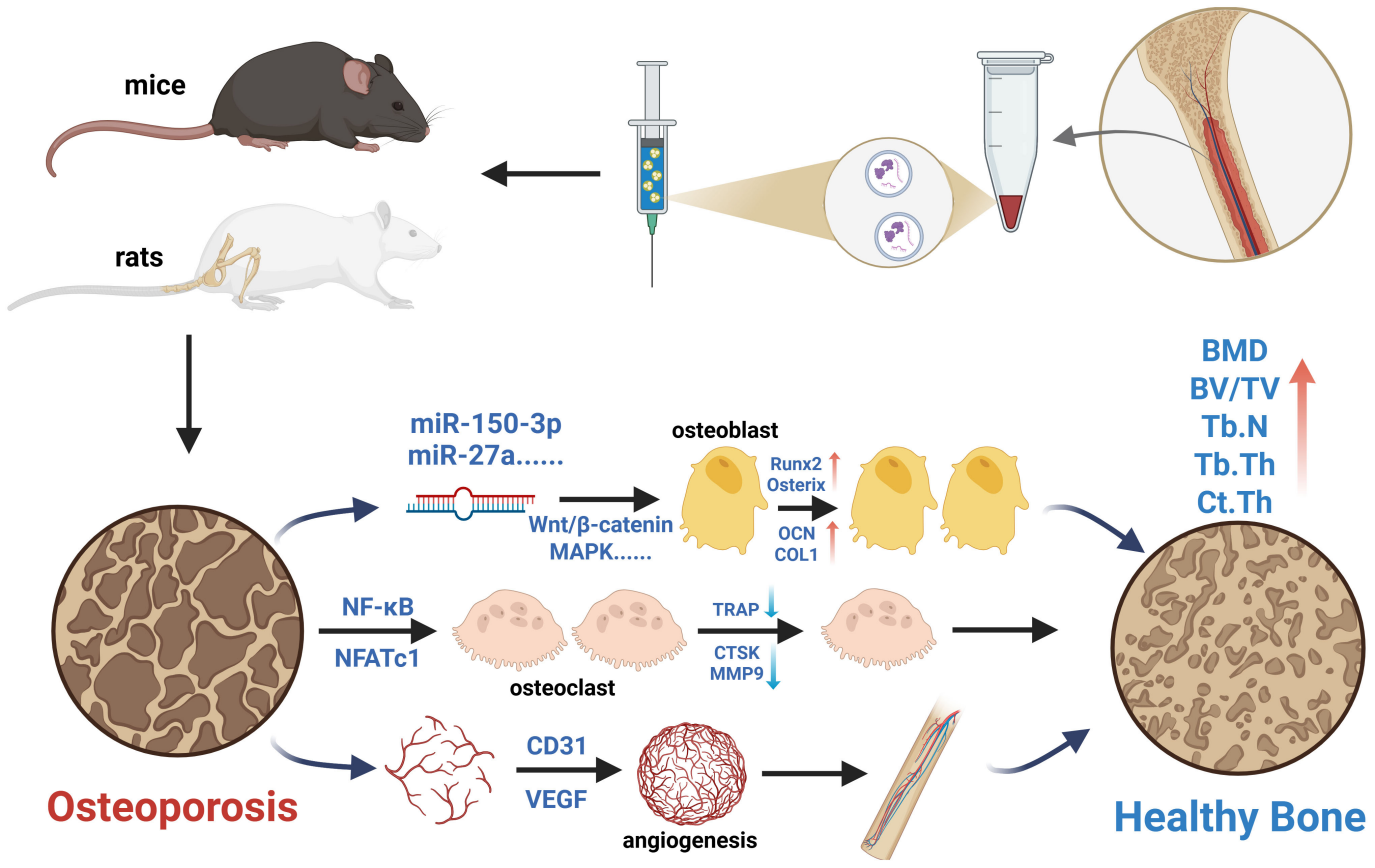
Schematic illustration of the potential mechanisms of MSC-EVs in the treatment of osteoporosis models. Created in https://BioRender.com.

Clinical drugs primarily improve bone strength and increase bone mass by inhibiting bone resorption and promoting bone formation, thereby regulating bone metabolism. Similar to clinical drugs, the therapeutic strategy of MSC-EVs also focuses on bone metabolism regulation (45). Mechanistically, multiple signaling pathways are involved in the bone remodeling process mediated by MSC-EVs in osteoporosis models, including the RANKL/RANK/OPG, WNT/β-catenin, Hippo, and PI3K/Akt pathways. Zhao et al. (46) found that BMSC-EVs promote osteoblast proliferation and differentiation *in vitro* via the MAPK pathway. Another study reported that BMSC-EVs reduce intracellular oxidative stress, promote DNA damage repair, and mitigate bone loss by activating the Wnt/β-catenin signaling pathway (47). Additionally, Li et al. (29) demonstrated that EVs derived from BMSCs facilitate bone repair in osteoporotic rats by delivering miR-186 through the Hippo pathway. Similar to these findings, this meta-analysis included seven studies investigating the potential mechanisms by which MSC-EVs improve osteoporosis, involving signaling pathways such as MAPK (25, 31), Wnt/β-catenin (21, 28, 37), PI3K/Akt (33), and NF-κB (38) (**Figure 10**). Given that the precise mechanisms underlying MSC-EVs treatment for osteoporosis remain unclear, further research is needed to supplement and refine current knowledge.

Currently, research on MSC-EVs intervention in osteoporotic animal models primarily focuses on the efficacy comparison of bone structural parameters while overlooking the therapeutic mechanisms and potential microscopic effects of MSC-EVs. These include the activity and function of osteoblasts, osteoclasts, human umbilical vein endothelial cells, and immune cells. Therefore, beyond bone metabolism regulation, future studies should place greater emphasis on exploring the angiogenic and immunomodulatory effects of MSC-EVs to further elucidate their therapeutic potential.

### Limitations

However, several study limitations must be considered. Firstly, significant differences in baseline characteristics among the included studies may have influenced the meta-analysis results, including variations in animal models, EVs preparation, and intervention characteristics (such as administration route, frequency, and treatment duration). Although subgroup analysis indicated that these factors were not significant contributors to heterogeneity, the interpretation of results should still be approached with caution. Future research should emphasize efficacy evaluation and comparison under standardized conditions based on animal models and EVs characteristics. Secondly, publication bias was present in all analytical results, which affected the quality of evidence in the meta-analysis. Future studies with larger sample sizes and standardized methodologies are needed to address this limitation. Thirdly, although sensitivity analysis confirmed the stability of the results, the absence of randomization and blinding procedures may have led to an overestimation of the therapeutic effects of MSC-EVs. Moreover, the analysis revealed varying degrees of heterogeneity and publication bias. Future studies should carefully consider negative or null findings to ensure the objectivity and robustness of the conclusions. Finally, most studies lacked safety data on MSC-EVs treatment, including toxicity and immunogenicity. Future research should prioritize the long-term monitoring of safety parameters to ensure the clinical applicability of MSC-EVs.

### Conclusions

In conclusion, this meta-analysis highlights the potential therapeutic value of MSC-EVs in osteoporotic animal models by assessing bone strength, bone mass, structural parameters, remodeling parameters, and biomechanical properties. The pooled analysis results provide evidence supporting the efficacy of MSC-EVs therapy in preclinical osteoporosis models. However, due to significant heterogeneity and publication bias, the findings should be interpreted with caution. Additionally, further studies are needed to establish standardized protocols and evaluate the safety of MSC-EVs interventions in more animal models and clinical trials.
