# Supplementary material for: Therapeutic effects of mesenchymal stem cell-derived extracellular vesicles in osteoporosis models: a systematic review and meta-analysis of preclinical studies
Source: Front Endocrinol (Lausanne). 2025 Sep 16;16:1625969. doi: 10.3389/fendo.2025.1625969 (PMC12479284; doi:10.3389/fendo.2025.1625969)
Supplement: Supplementary file 1 [file DataSheet1.docx]

**Supplementary Materials**

| **Supplementary Table 1.** Search Strages in four databases (Search Cut-off Date: January 1, 2025). | | | |
| --- | --- | --- | --- |
| **Databases** | **No.** | **Query** | **Results** |
| **Pubmed** | #1 | “mesenchymal stem cell-derived extracellular vesicles” OR “MSC-EVs” OR “MSC-derived extracellular vesicles” OR “mesenchymal stromal cell-derived extracellular vesicles” OR “extracellular vesicle” OR “exovesicles” OR “exovesicle” OR “apoptotic bodies” OR “exosomes” OR “microvesicles” | 43,934 |
|  | #2 | “osteoporosis” OR “osteoporotic bone loss” OR “ovariectomized” OR “OVX” OR “glucocorticoid-induced osteoporosis” OR “bone microarchitecture” OR “bone loss” OR “bone resorption” | 196,072 |
|  | #3 | #1 AND #2 | 380 |
| **Cochrane Library** | #4 | (mesenchymal stem cell-derived extracellular vesicles):ab,ti,kw OR (MSC-EVs):ab,ti,kw OR (MSC-derived extracellular vesicles):ab,ti,kw OR (mesenchymal stromal cell-derived extracellular vesicles):ab,ti,kw OR (extracellular vesicle):ab,ti,kw OR (exovesicles):ab,ti,kw OR (exovesicle):ab,ti,kw OR (apoptotic bodies):ab,ti,kw OR (exosomes):ab,ti,kw OR (microvesicles):ab,ti,kw | 465 |
|  | #5 | (osteoporosis):ab,ti,kw OR (osteoporotic bone loss):ab,ti,kw OR (ovariectomized):ab,ti,kw OR (OVX):ab,ti,kw OR (glucocorticoid-induced osteoporosis):ab,ti,kw OR (bone microarchitecture):ab,ti,kw OR (bone loss):ab,ti,kw OR (bone resorption):ab,ti,kw | 24,453 |
|  | #6 | #4 AND #5 | 6 |
| **Web of Science** | #7 | TS=(mesenchymal stem cell-derived extracellular vesicles OR MSC-EVs OR MSC-derived extracellular vesicles OR mesenchymal stromal cell-derived extracellular vesicles OR extracellular vesicle OR exovesicles OR exovesicle OR apoptotic bodies OR exosomes OR microvesicles) | 85,761 |
|  | #8 | TS=(osteoporosis OR osteoporotic bone loss OR ovariectomized OR OVX OR glucocorticoid-induced osteoporosis OR bone microarchitecture OR bone loss OR bone resorption) | 261,719 |
|  | #9 | #7 AND #8 | 1,145 |
| **Embase** | #10 | ('mesenchymal stem cell-derived extracellular vesicles':ab,ti,kw OR 'MSC-EVs':ab,ti,kw OR 'MSC-derived extracellular vesicles':ab,ti,kw OR 'mesenchymal stromal cell-derived extracellular vesicles':ab,ti,kw OR 'extracellular vesicle':ab,ti,kw OR 'exovesicles':ab,ti,kw OR 'exovesicle':ab,ti,kw OR 'apoptotic bodies':ab,ti,kw OR 'exosomes':ab,ti,kw OR 'microvesicles':ab,ti,kw) | 54,990 |
|  | #11 | ('osteoporosis':ab,ti,kw OR 'osteoporotic bone loss':ab,ti,kw OR 'ovariectomized':ab,ti,kw OR 'OVX':ab,ti,kw OR 'glucocorticoid-induced osteoporosis':ab,ti,kw OR 'bone microarchitecture':ab,ti,kw OR 'bone loss':ab,ti,kw OR 'bone resorption':ab,ti,kw) | 231,962 |
|  | #12 | #10 AND #11 | 436 |
| **Total** | #13 | #3 AND #6 AND #9 AND #12 | 1,967 |

**Supplementary figures**

**
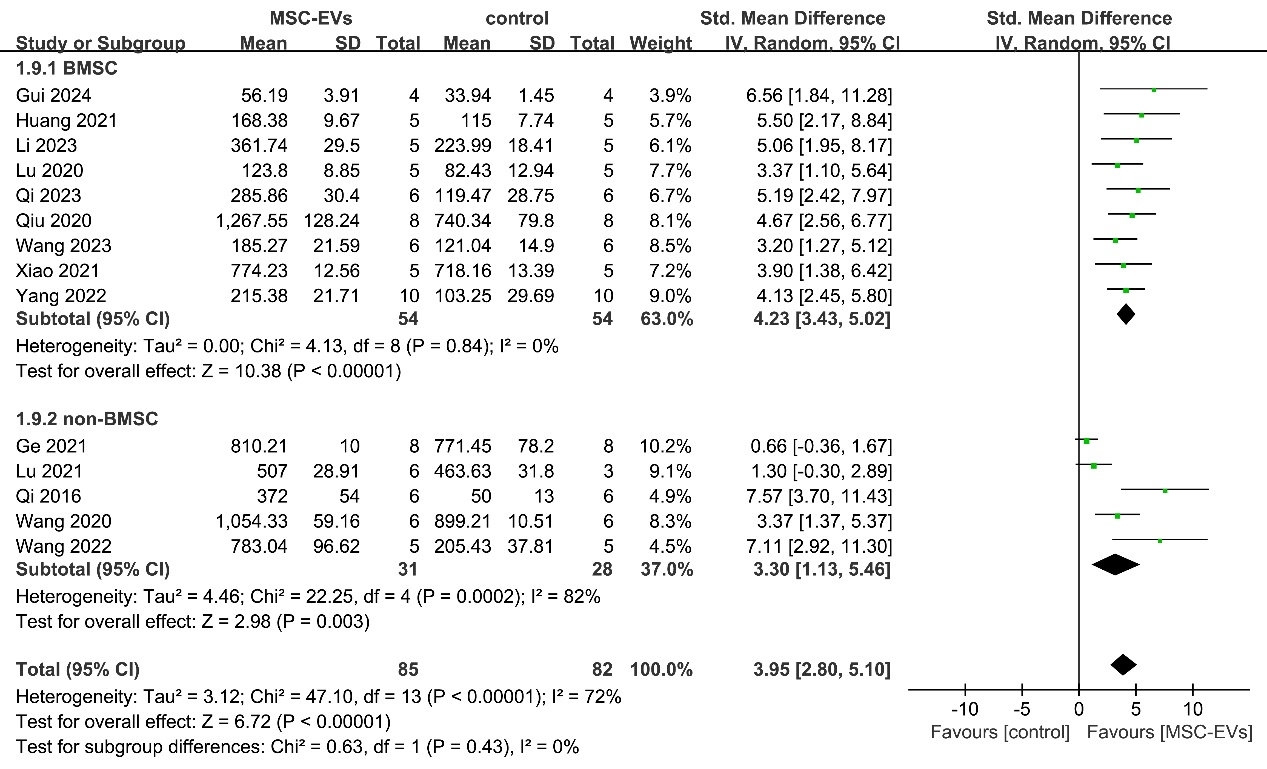
**

**Figure S1.** Subgroup analysis based on different MSC-EVs sources for bone mineral density (BMD). Data are presented as standardized mean difference (SMD) with 95% confidence intervals (CI).


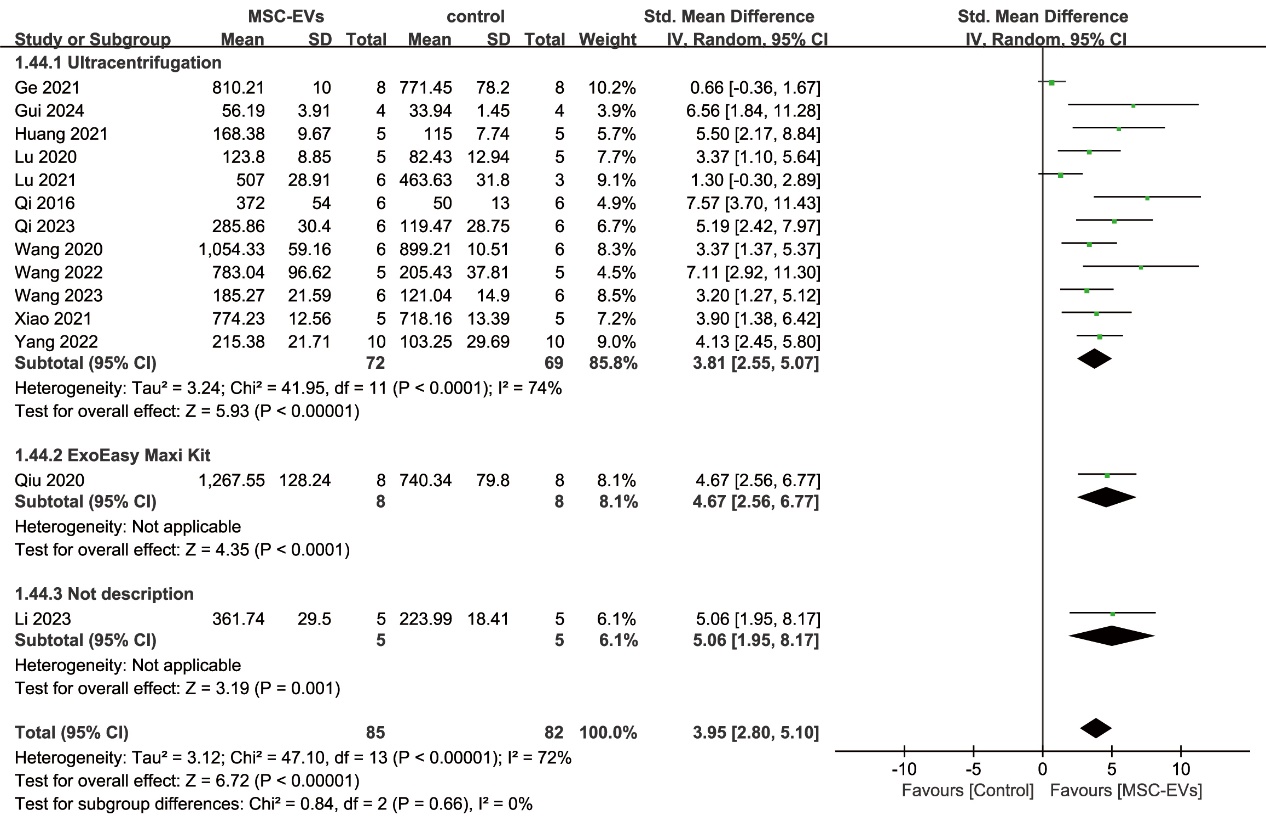


**Figure S2.** Subgroup analysis of bone mineral density (BMD) based on different MSC-EVs isolation methods. Data are presented as standardized mean difference (SMD) with 95% confidence intervals (CI).


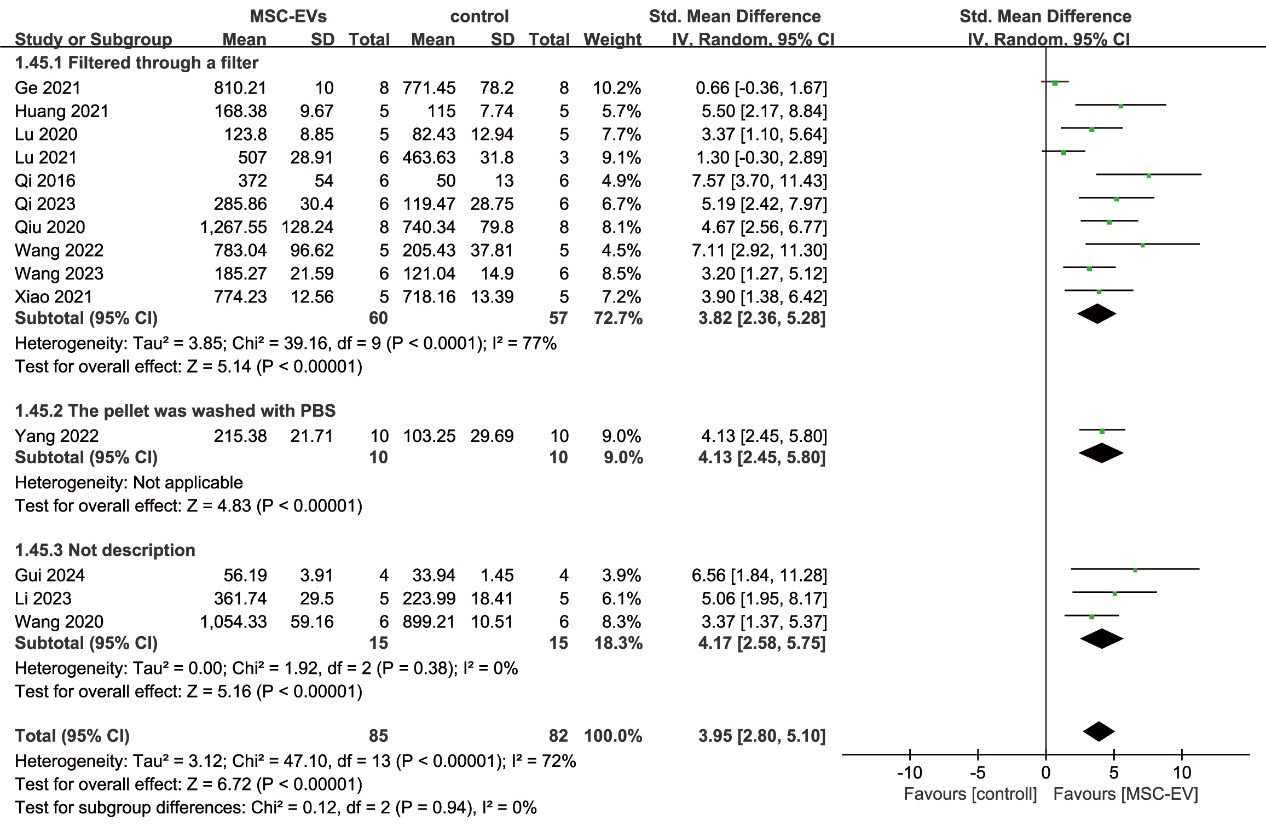


**Figure S3.** Subgroup analysis of bone mineral density (BMD) based on different MSC-EV purification methods. Data are presented as standardized mean difference (SMD) with 95% confidence intervals (CI).


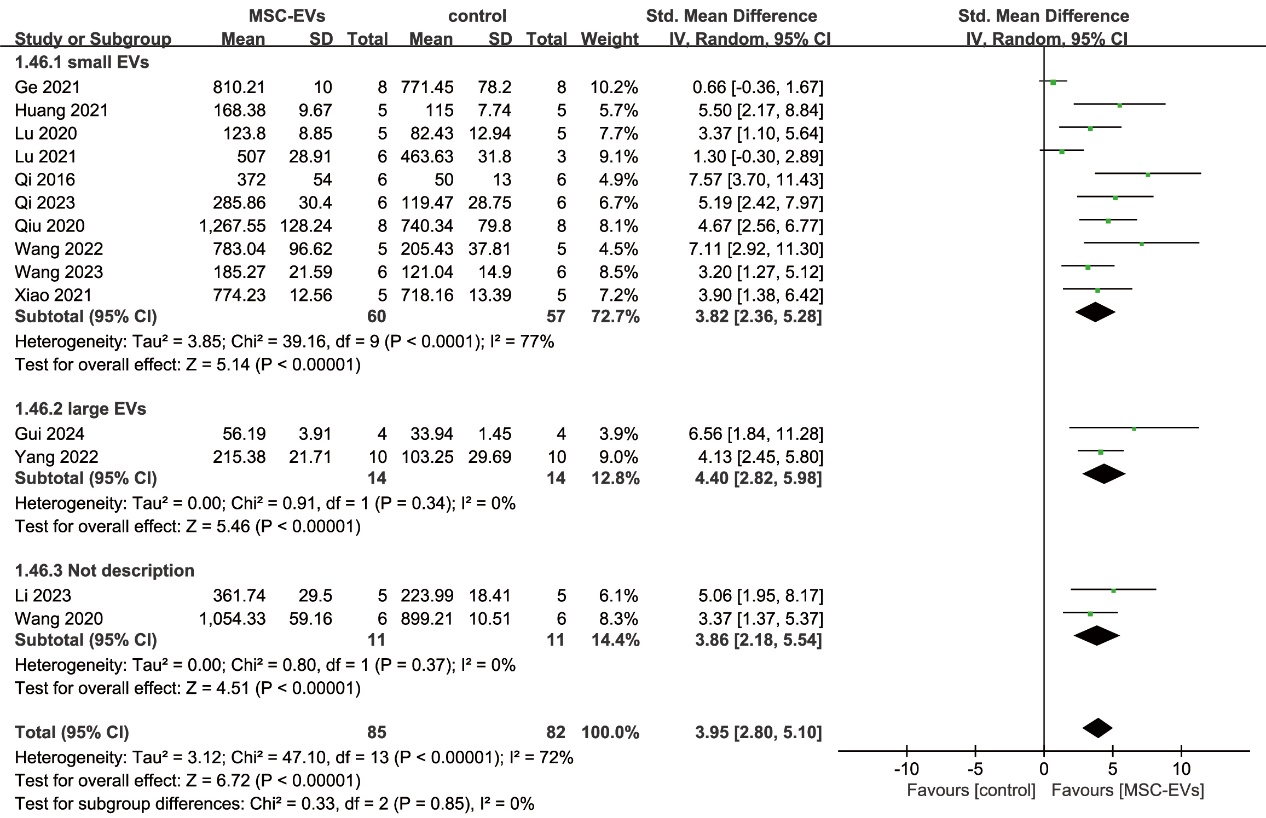


**Figure S4.** Subgroup analysis of bone mineral density (BMD) based on different MSC-EV sizes. Data are presented as standardized mean difference (SMD) with 95% confidence intervals (CI).

**
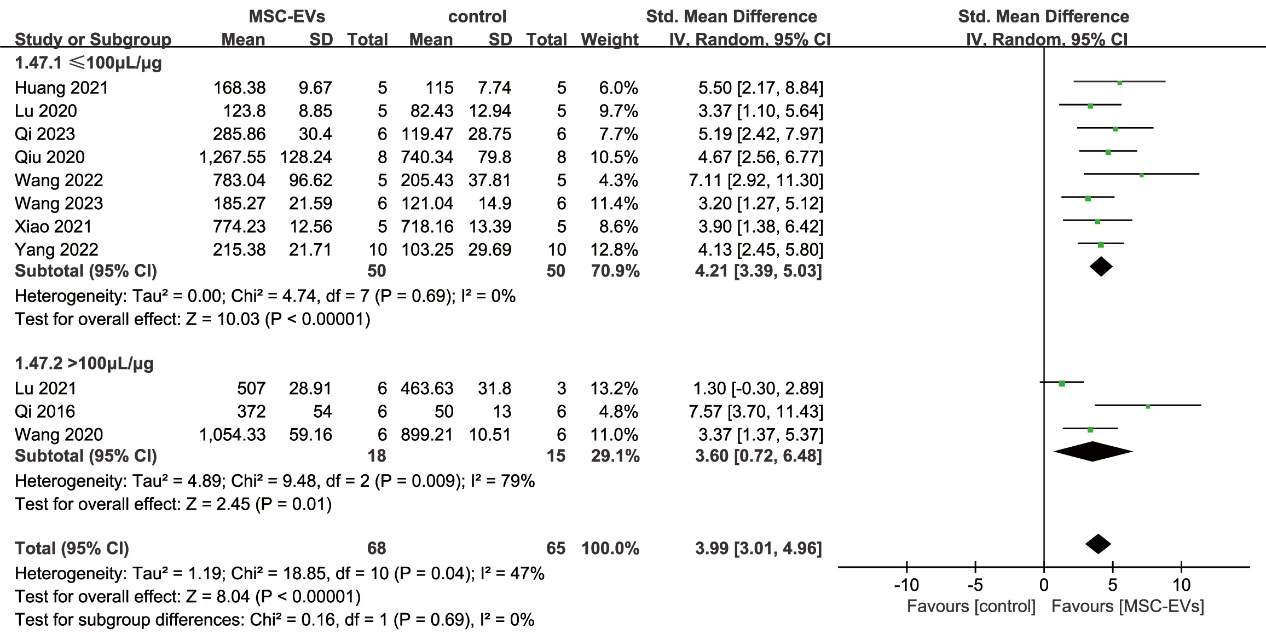
**

**Figure S5.** Subgroup analysis of Bone mineral density (BMD) based on different MSC-EV intervention doses. Data are presented as standardized mean difference (SMD) with 95% confidence intervals (CI).


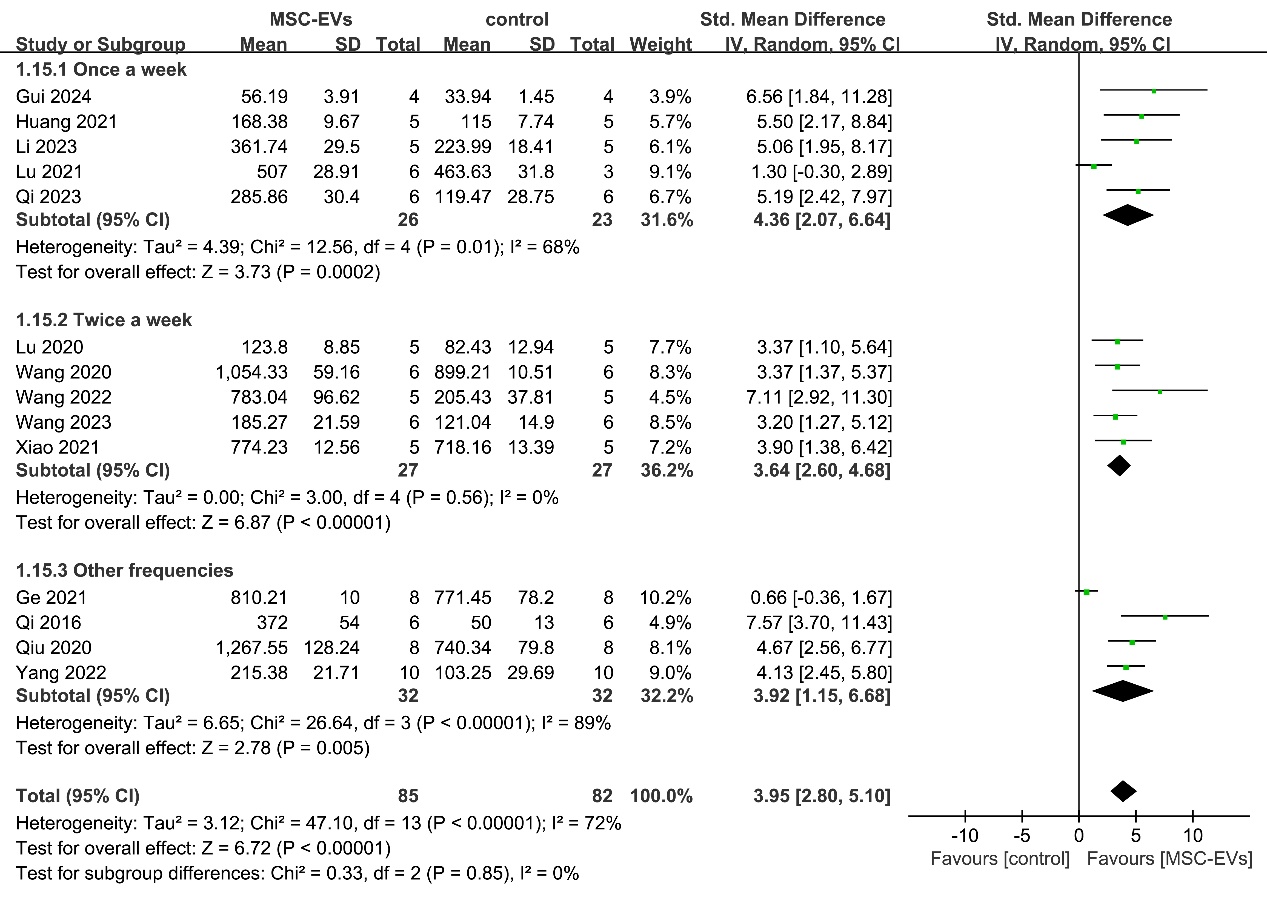
**Figure S6.** Subgroup analysis of bone mineral density (BMD) based on different administration frequencies. Data are presented as standardized mean difference (SMD) with 95% confidence intervals (CI).


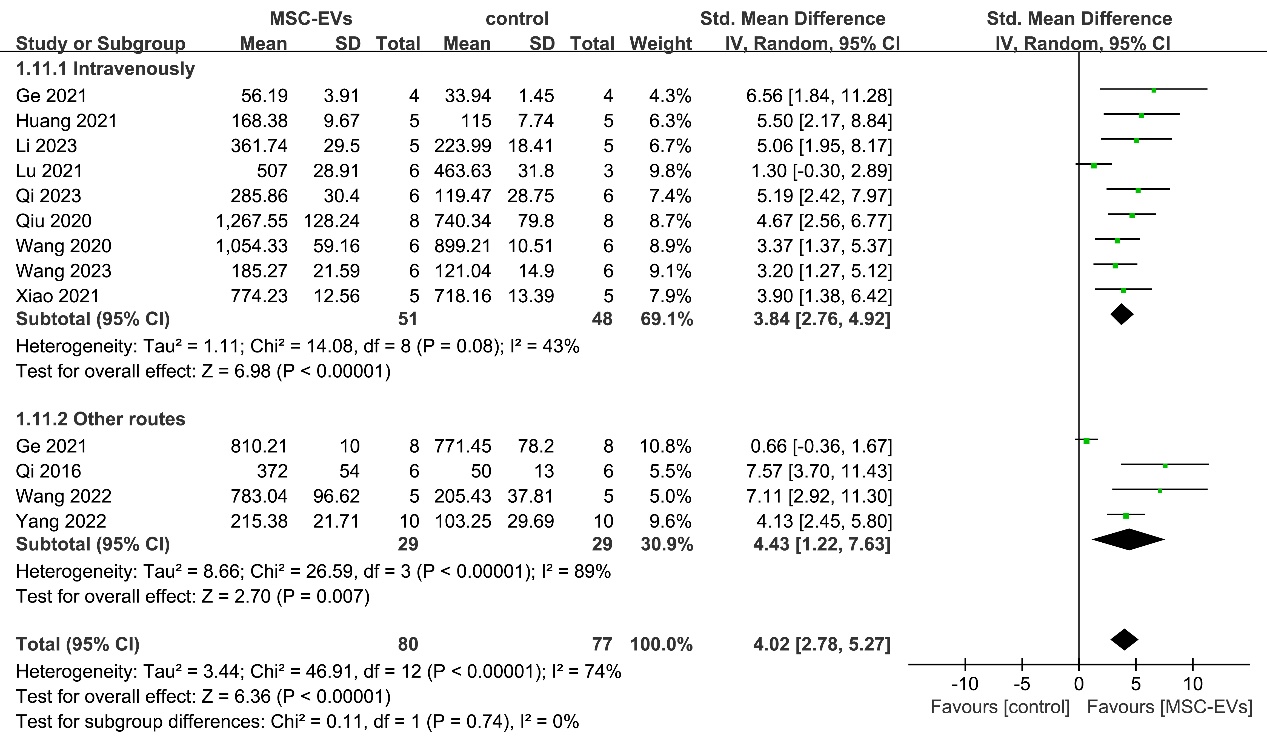


**Figure S7.** Subgroup analysis of bone mineral density (BMD) based on different administration routes. Data are presented as standardized mean difference (SMD) with 95% confidence intervals (CI).


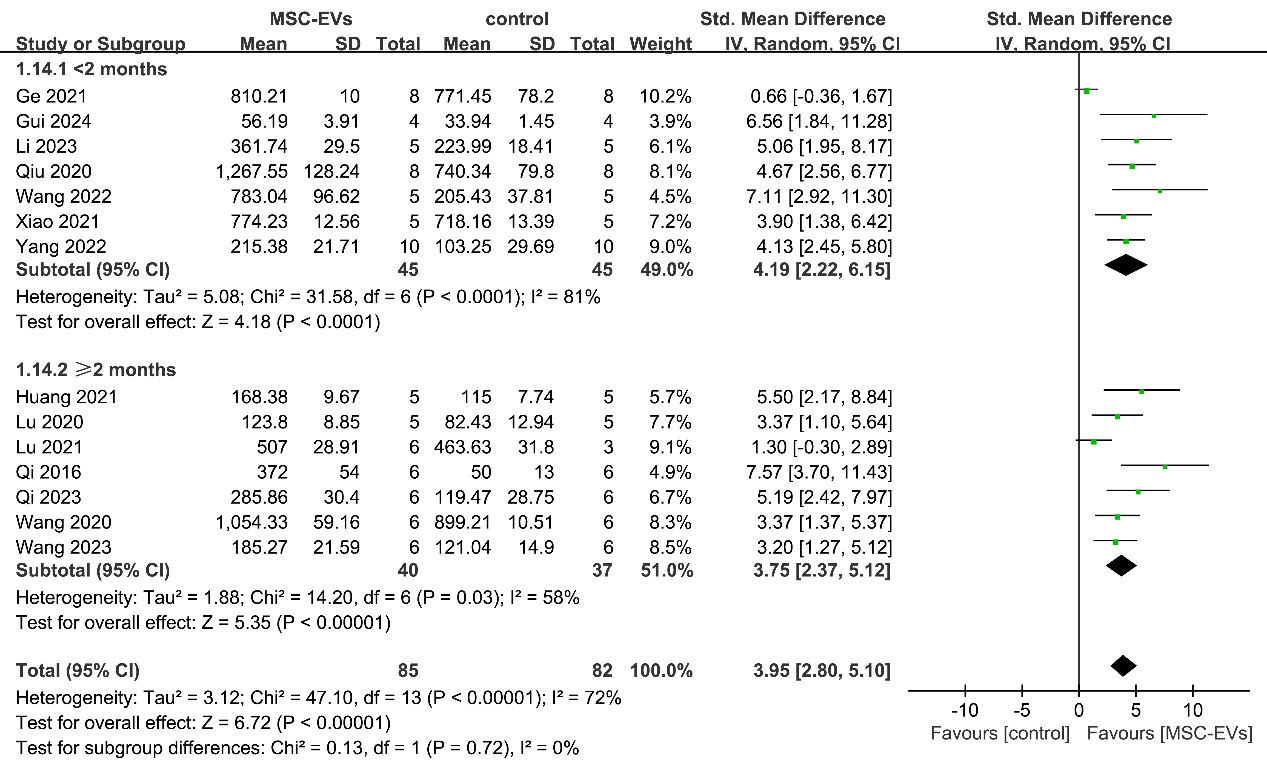


**Figure S8.** Subgroup analysis of bone mineral density (BMD) based on different treatment durations. Data are presented as standardized mean difference (SMD) with 95% confidence intervals (CI).

**
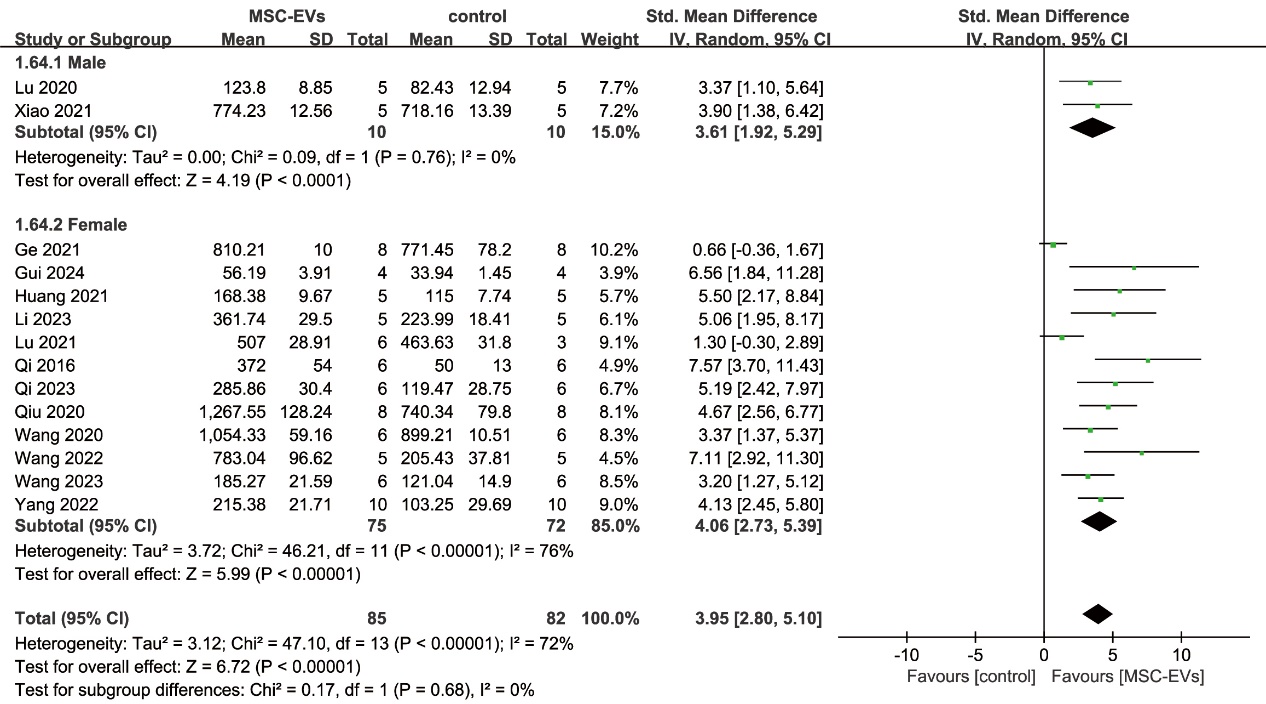
**

**Figure S9.** Subgroup analysis of bone mineral density (BMD) based on different animal sexes. Data are presented as standardized mean difference (SMD) with 95% confidence intervals (CI).


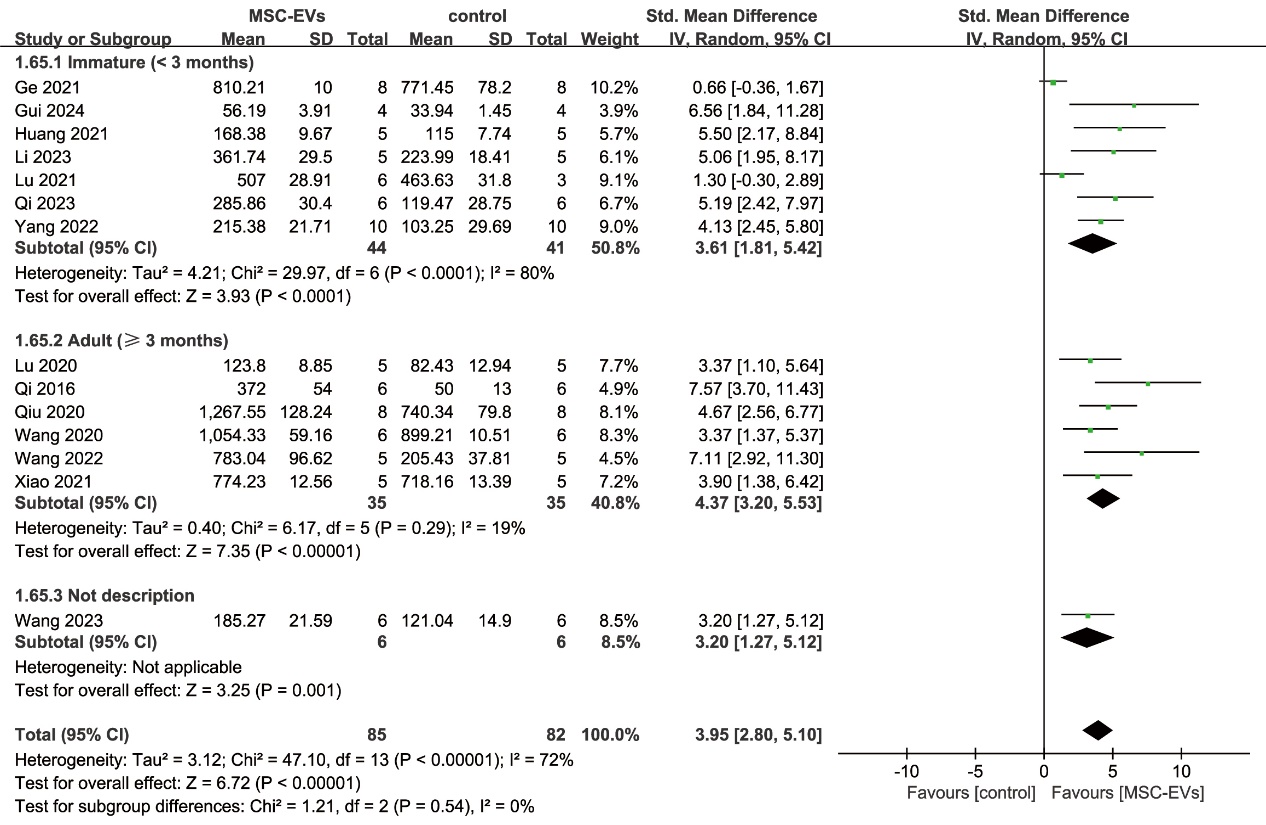


**Figure S10.** Subgroup analysis of bone mineral density (BMD) based on different animal ages. Data are presented as standardized mean difference (SMD) with 95% confidence intervals (CI).

**
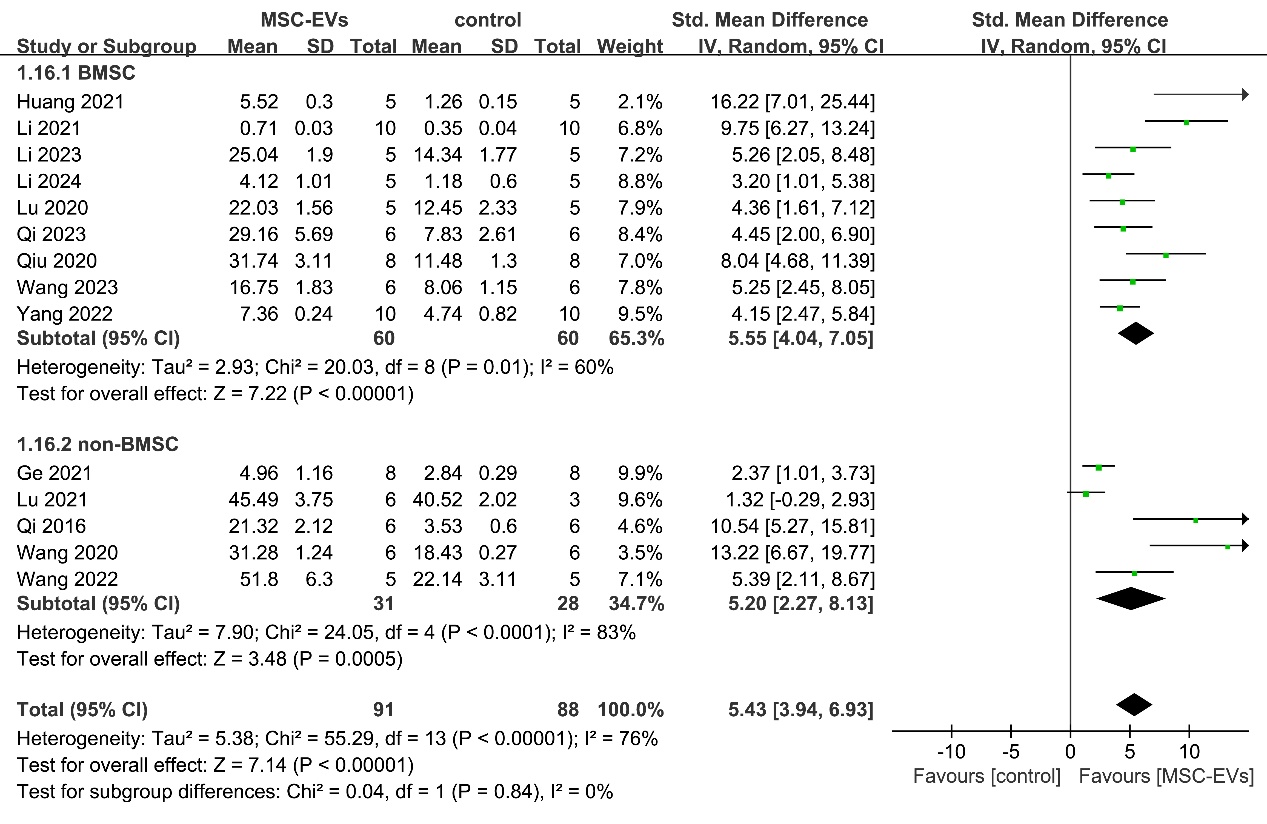
**

**Figure S11.** Subgroup analysis based on different MSC-EVs sources for BV/TV. Data are presented as standardized mean difference (SMD) with 95% confidence intervals (CI).


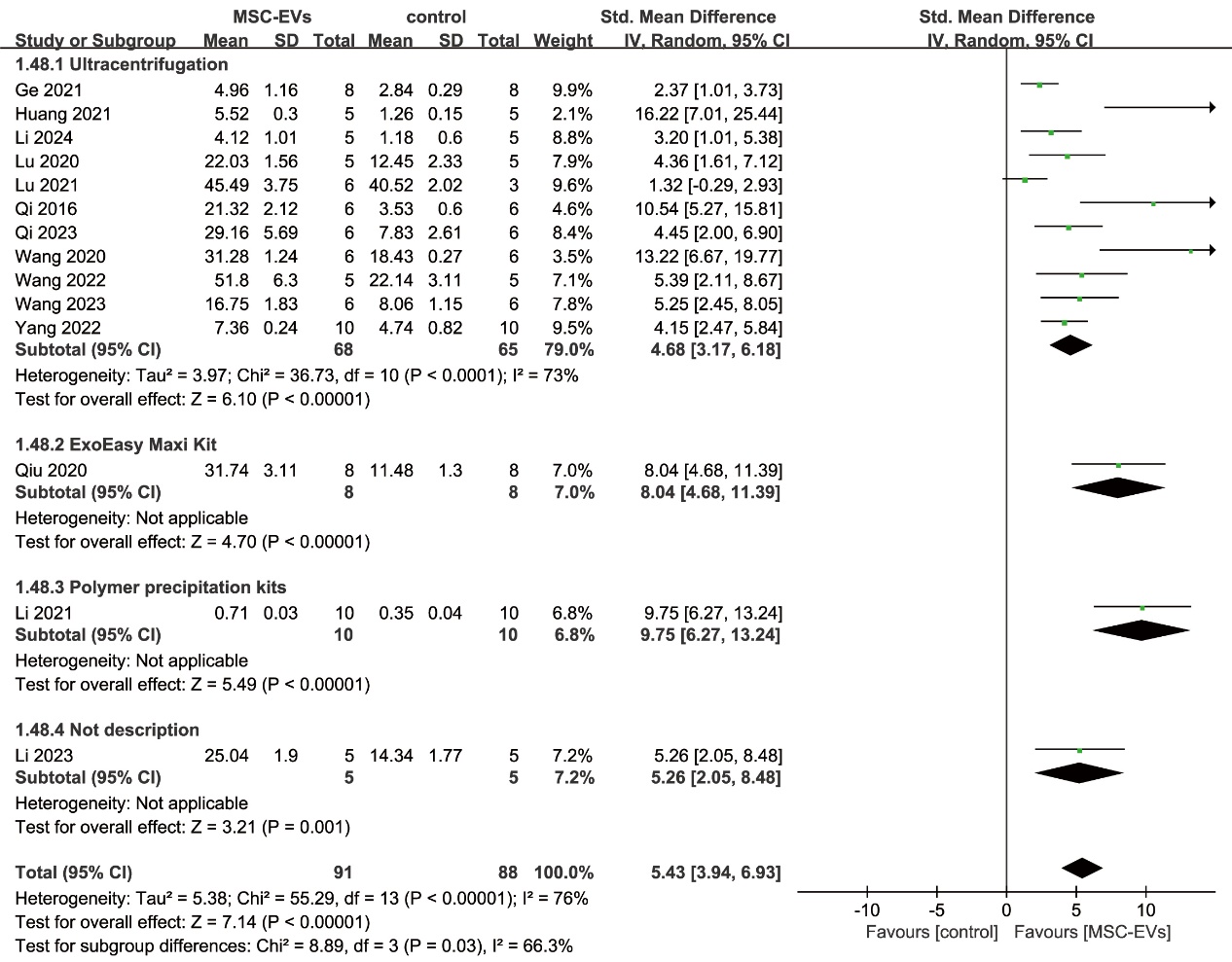


**Figure S12.** Subgroup analysis of BV/TV based on different MSC-EVs isolation methods. Data are presented as standardized mean difference (SMD) with 95% confidence intervals (CI).


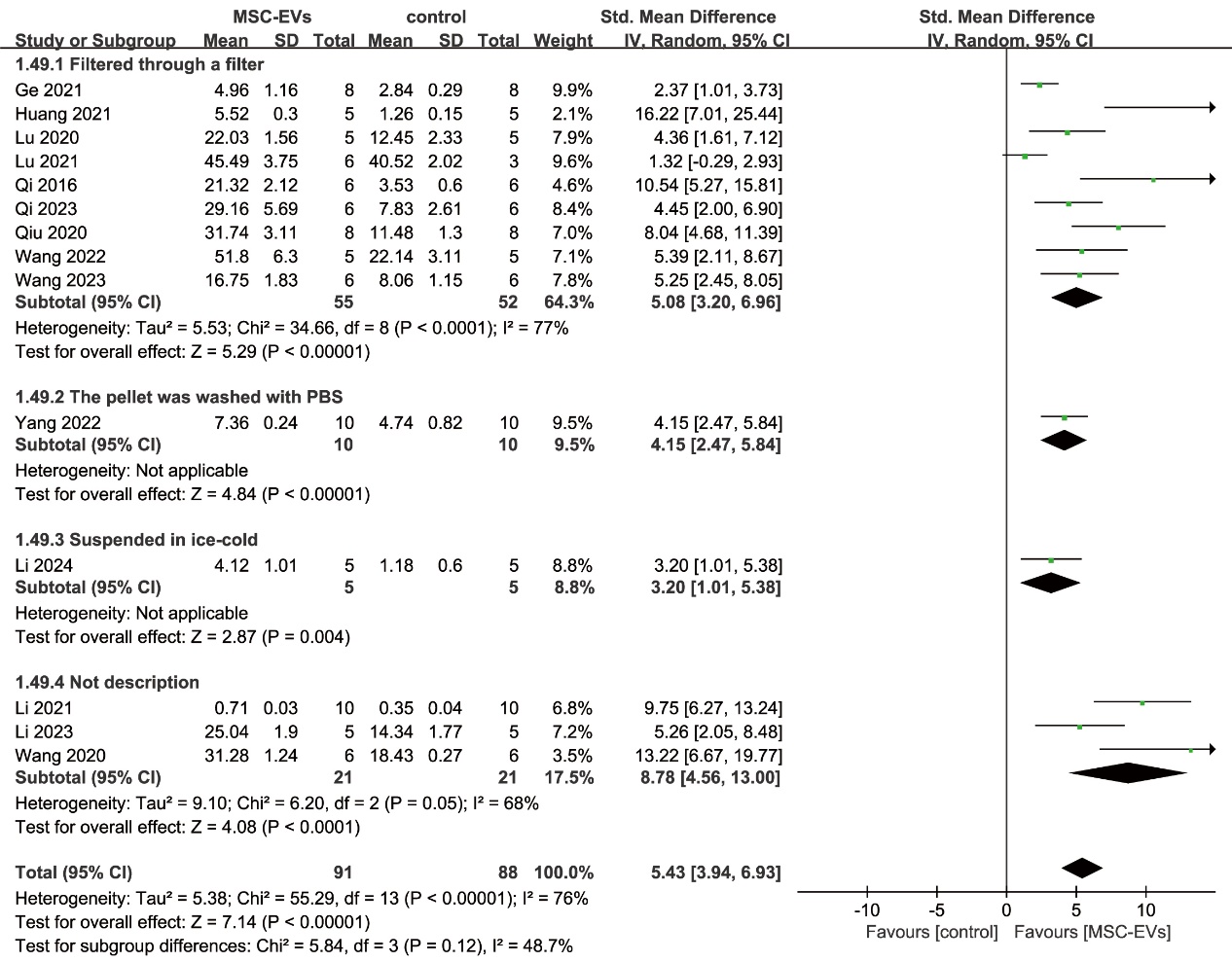


**Figure S13.** Subgroup analysis of BV/TV based on different MSC-EVs purification methods. Data are presented as standardized mean difference (SMD) with 95% confidence intervals (CI).


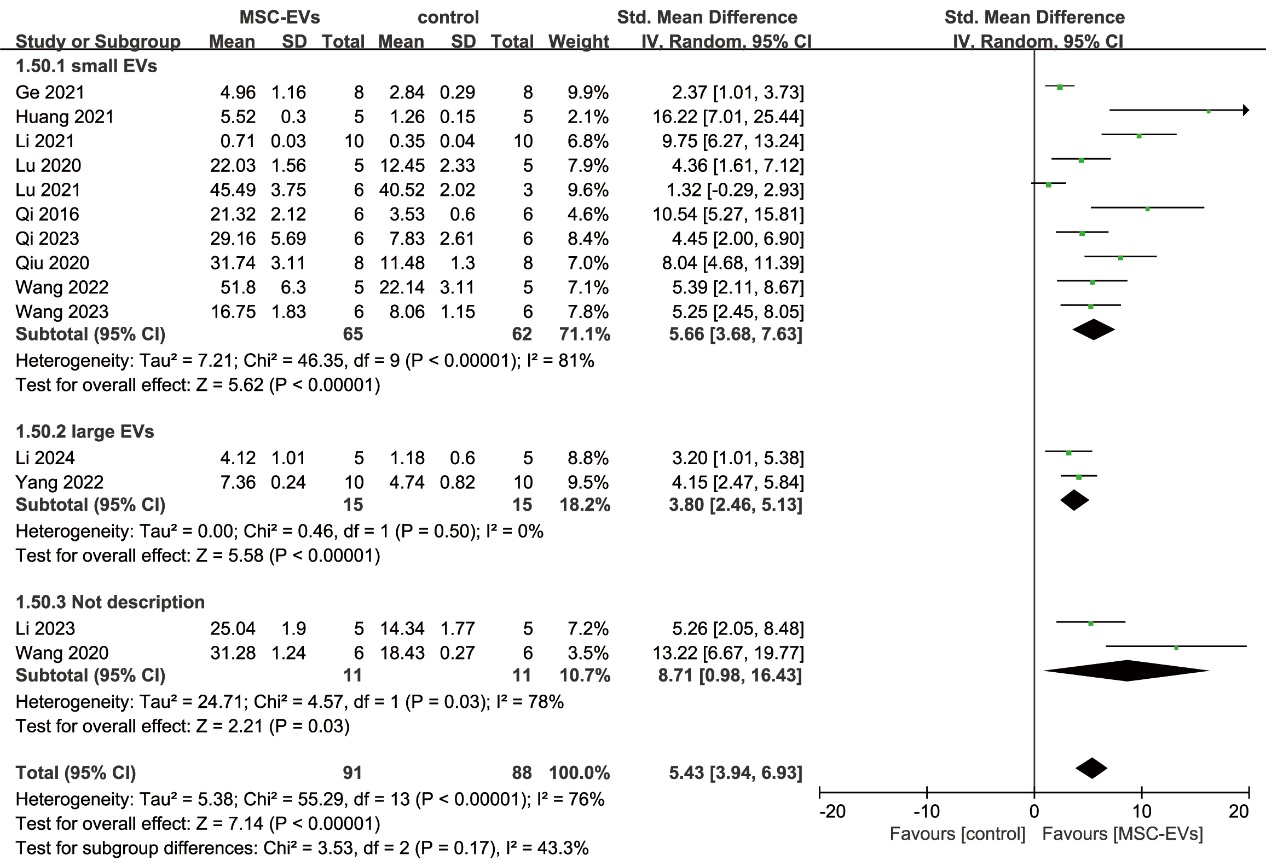


**Figure S14.** Subgroup analysis of BV/TV based on different MSC-EV sizes. Data are presented as standardized mean difference (SMD) with 95% confidence intervals (CI).

**
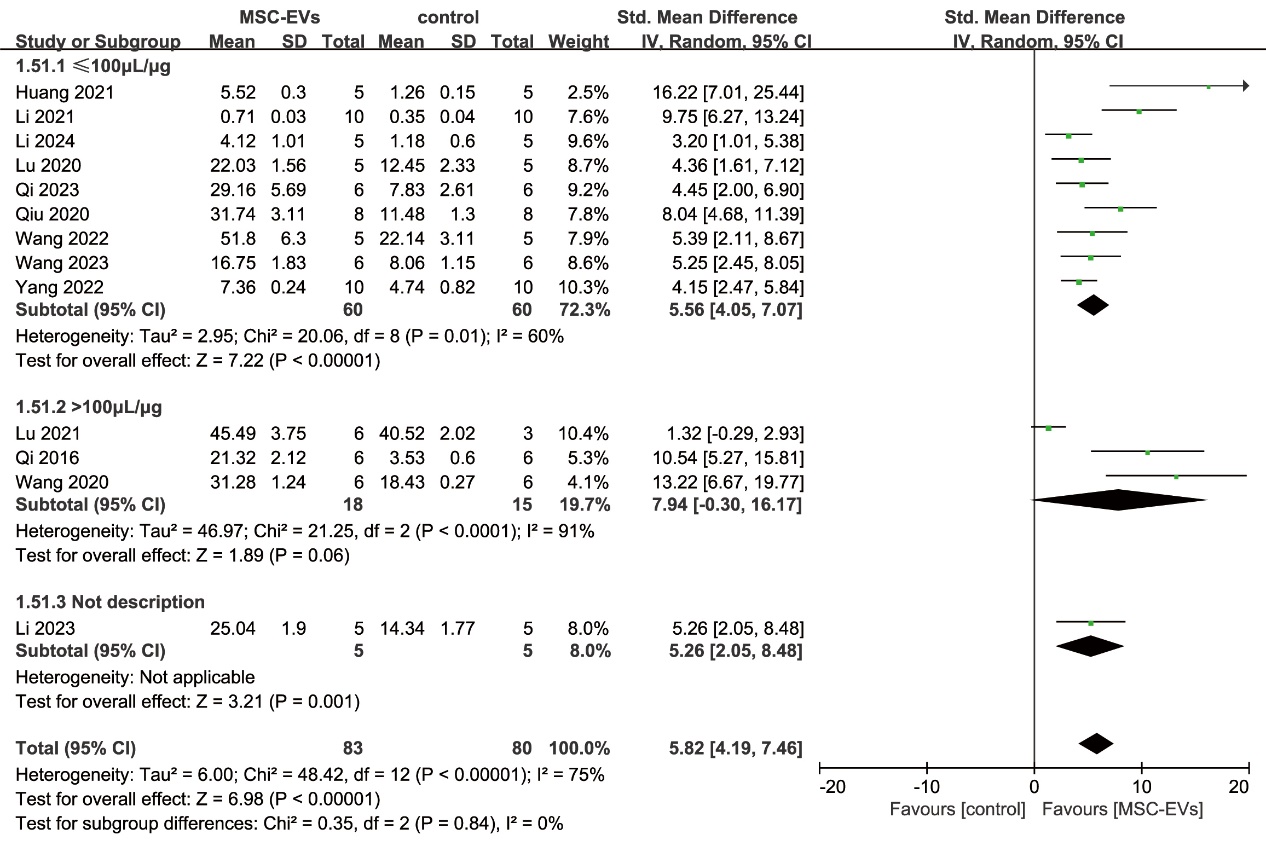
**

**Figure S15.** Subgroup analysis of BV/TV based on different MSC-EV intervention doses. Data are presented as standardized mean difference (SMD) with 95% confidence intervals (CI).

**
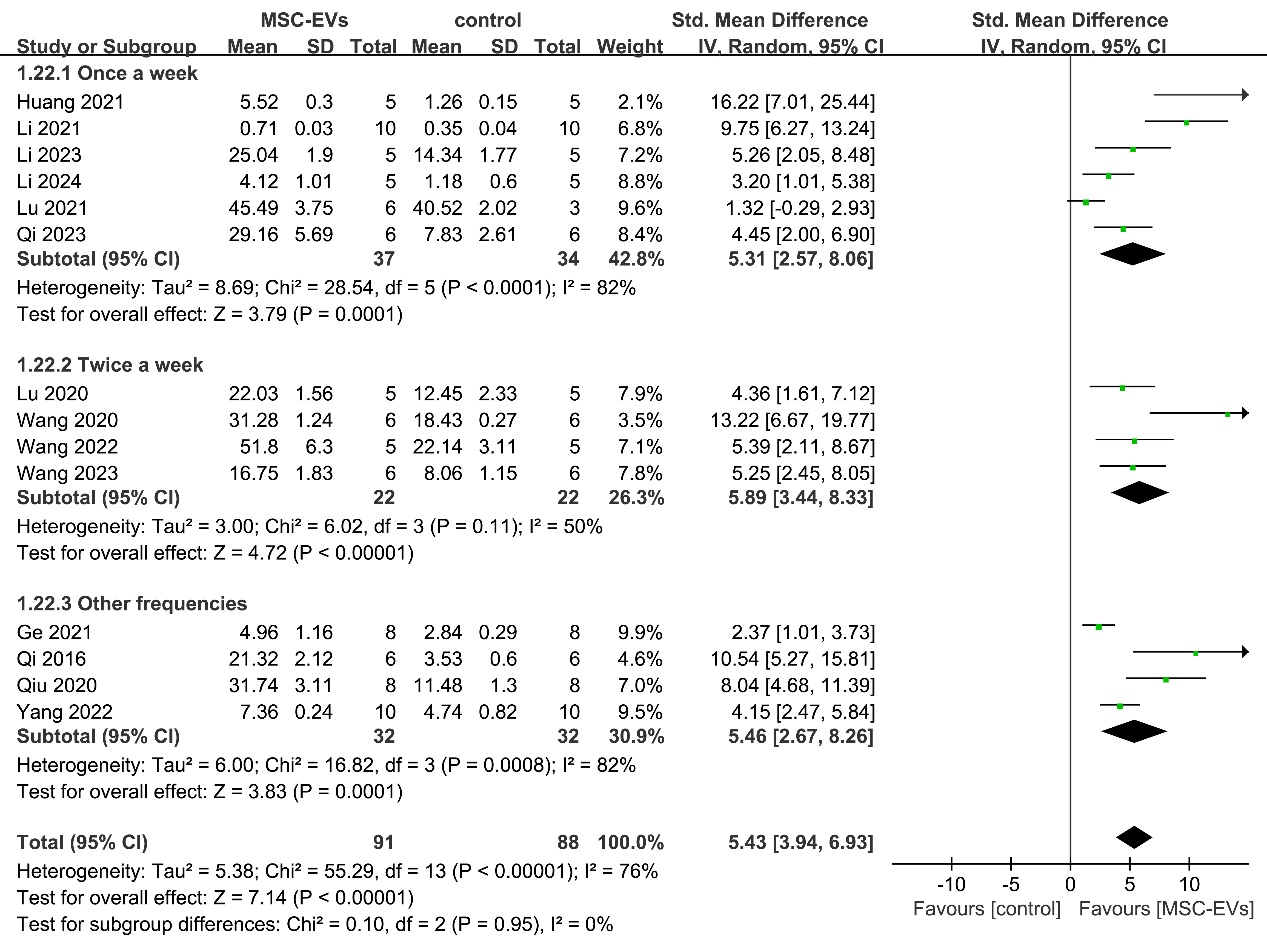
**

**Figure S16.** Subgroup analysis of BV/TV based on different administration frequencies. Data are presented as standardized mean difference (SMD) with 95% confidence intervals (CI).

**
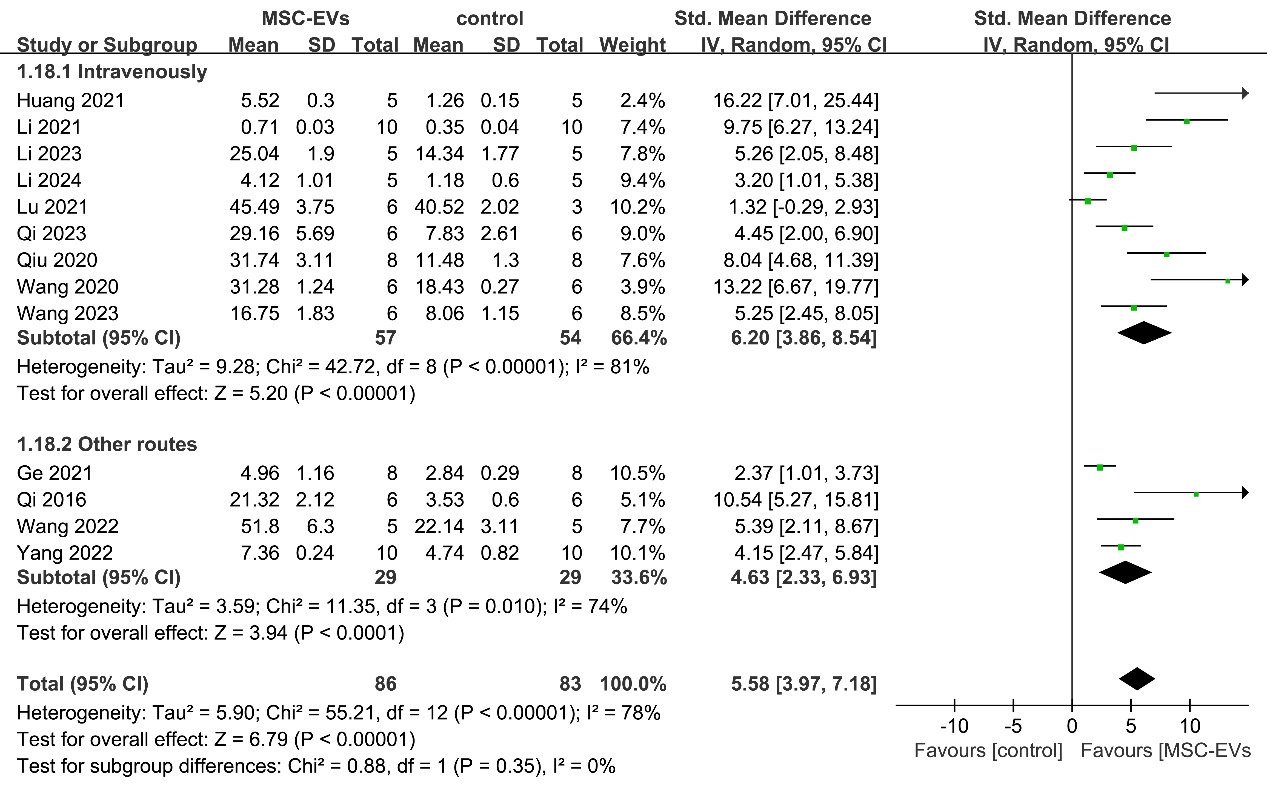
**

**Figure S17.** Subgroup analysis of BV/TV based on different administration routes. Data are presented as standardized mean difference (SMD) with 95% confidence intervals (CI).

**
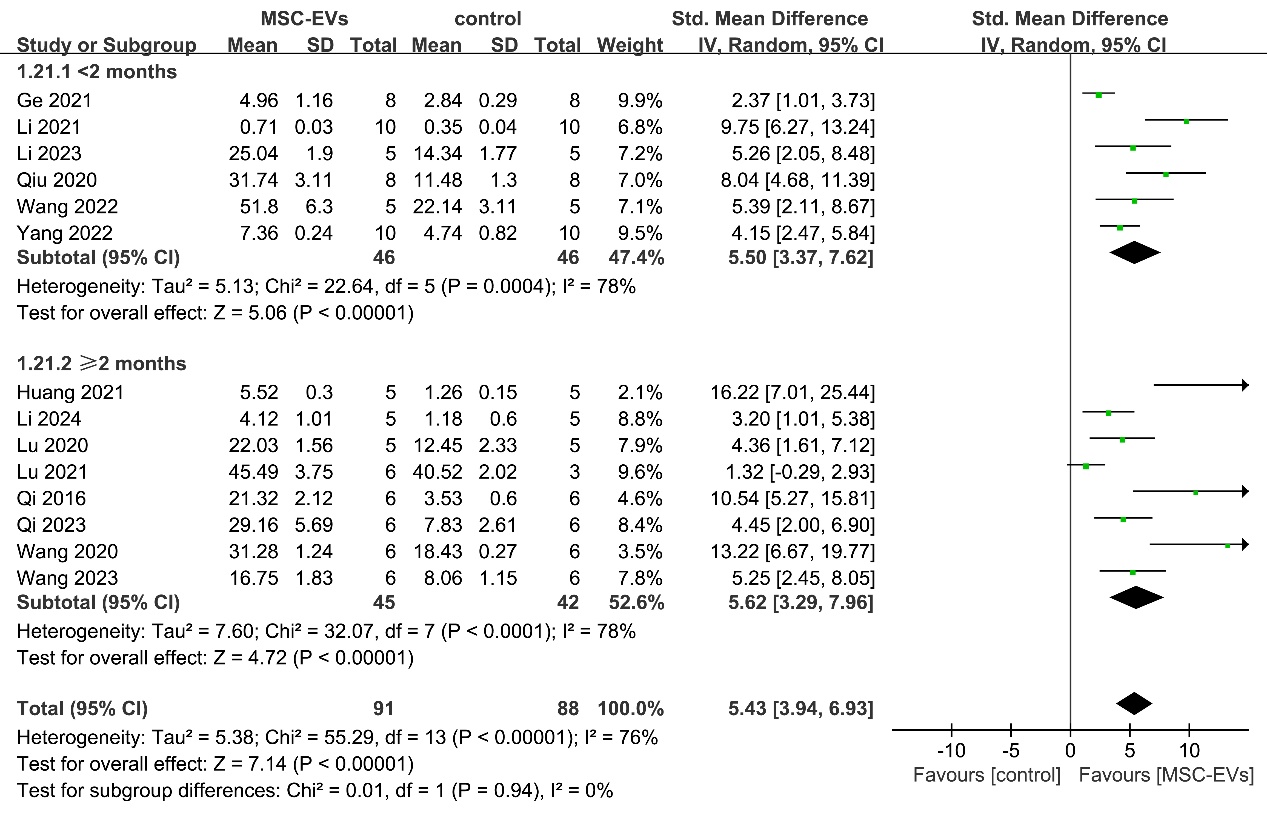
**

**Figure S18.** Subgroup analysis of BV/TV based on different treatment durations. Data are presented as standardized mean difference (SMD) with 95% confidence intervals (CI).

**
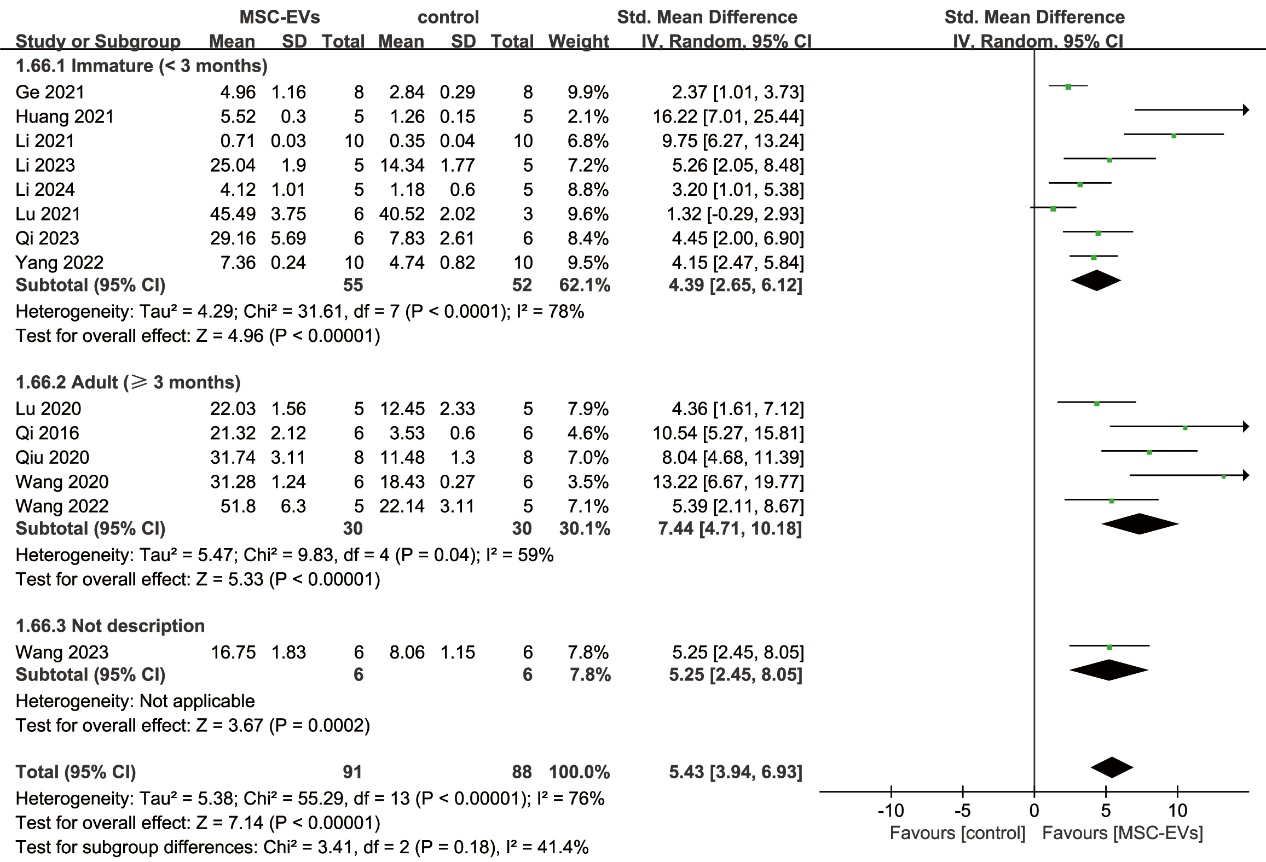
**

**Figure S19.** Subgroup analysis of BV/TV based on different animal ages. Data are presented as standardized mean difference (SMD) with 95% confidence intervals (CI).


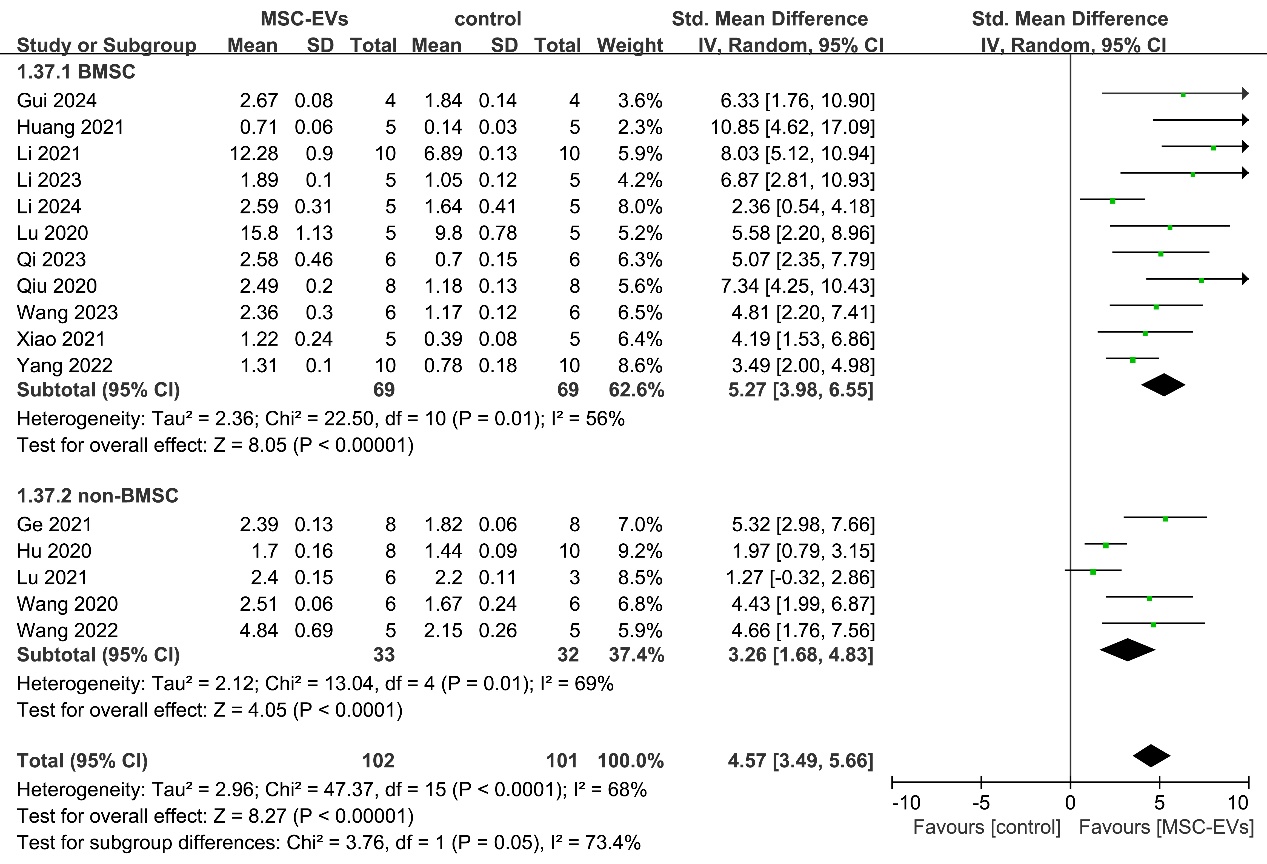


**Figure S20.** Subgroup analysis based on different MSC-EVs sources for Tb. N. Data are presented as standardized mean difference (SMD) with 95% confidence intervals (CI).


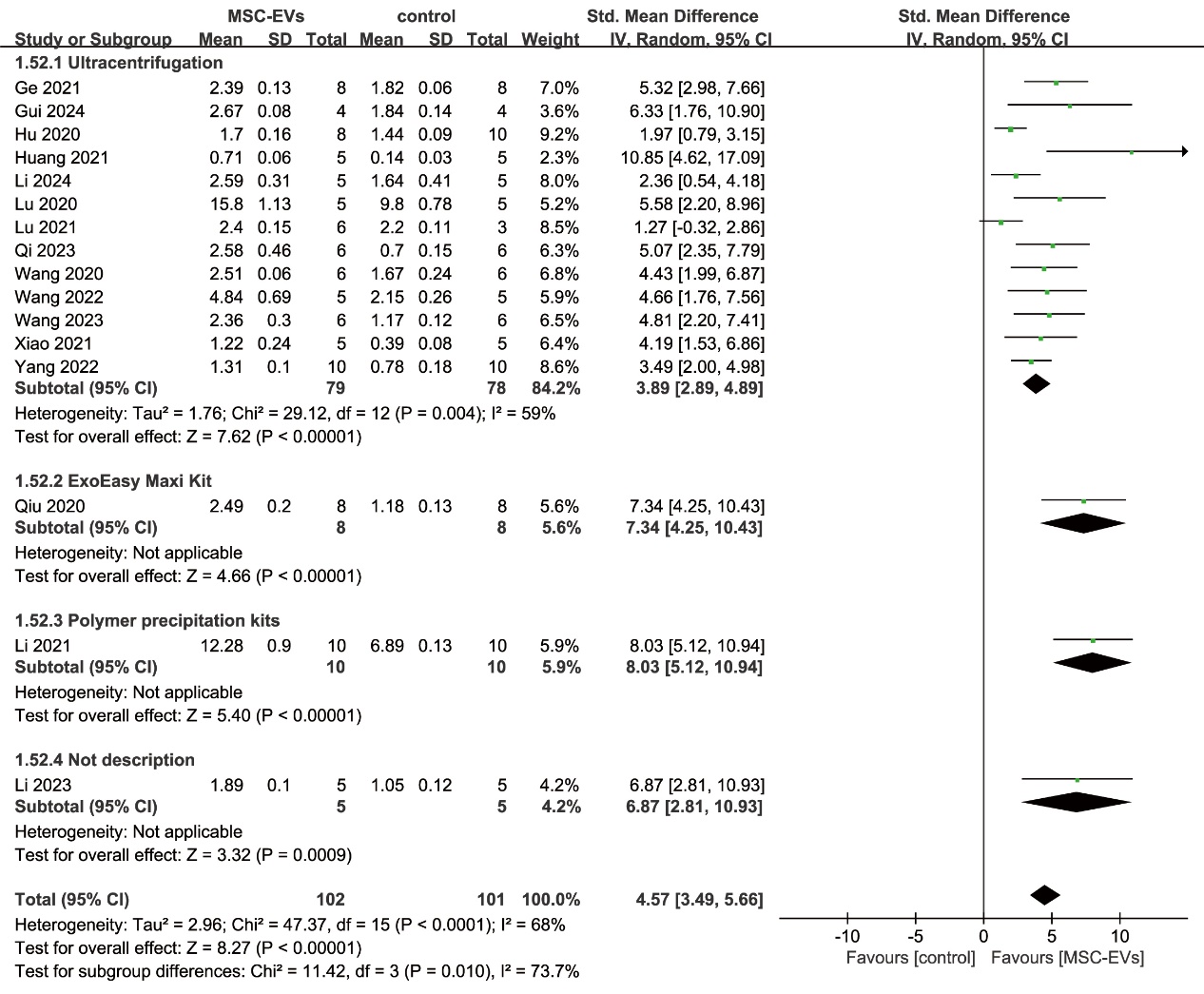


**Figure S21.** Subgroup analysis of Tb. N based on different MSC-EVs isolation methods. Data are presented as standardized mean difference (SMD) with 95% confidence intervals (CI).

**
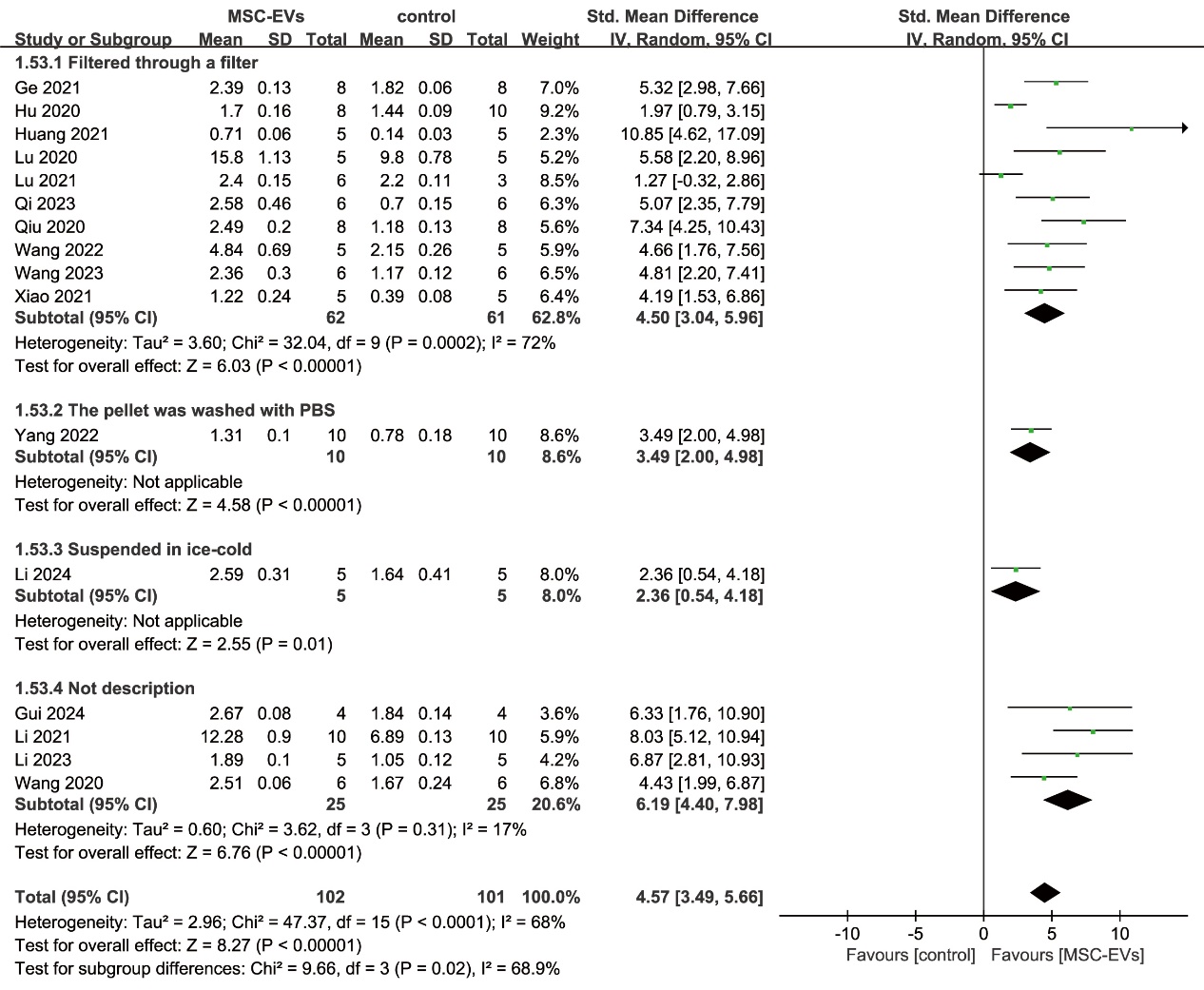
**

**Figure S22.** Subgroup analysis of Tb. N based on different MSC-EVs purification methods. Data are presented as standardized mean difference (SMD) with 95% confidence intervals (CI).


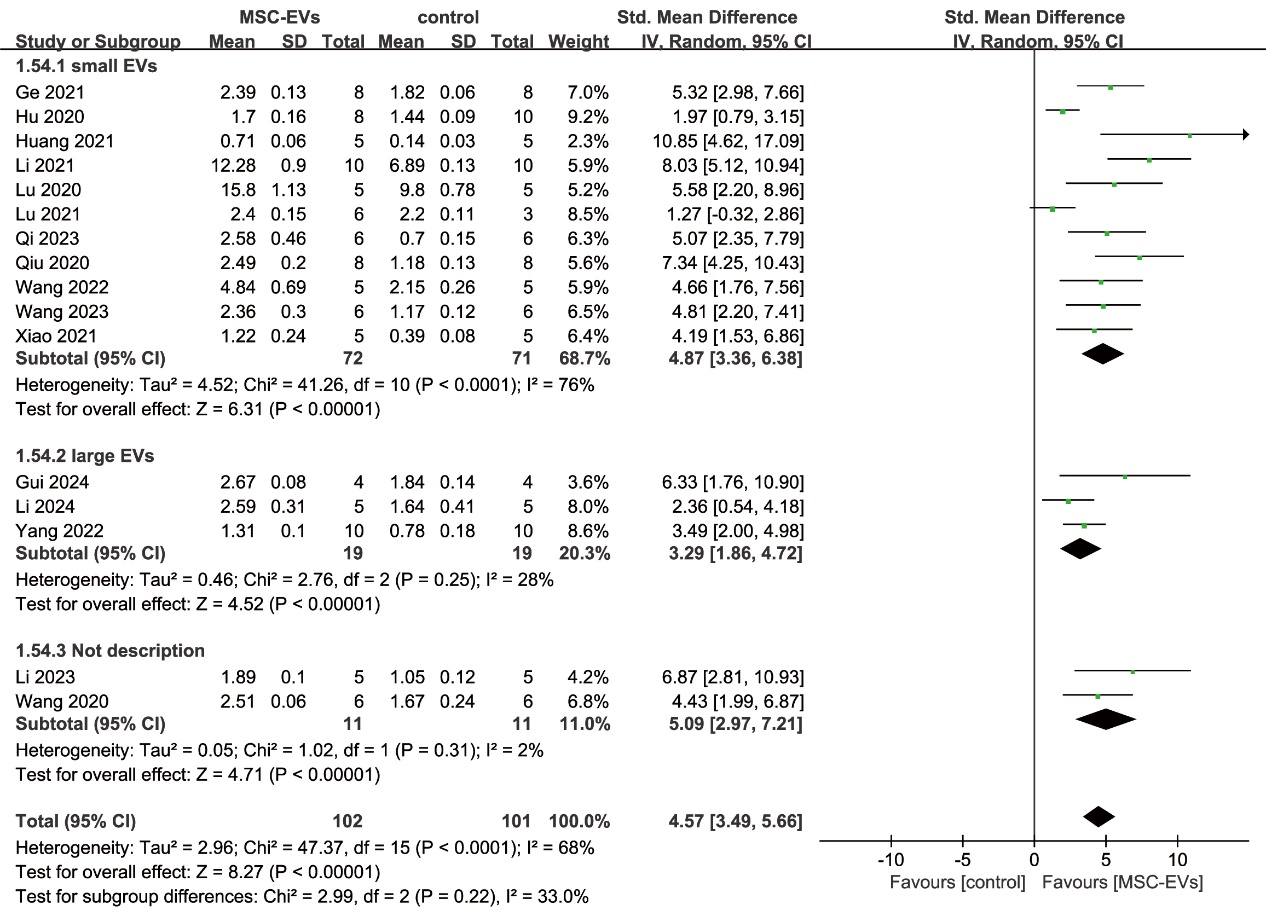


**Figure S23.** Subgroup analysis of Tb. N based on different MSC-EV sizes. Data are presented as standardized mean difference (SMD) with 95% confidence intervals (CI).


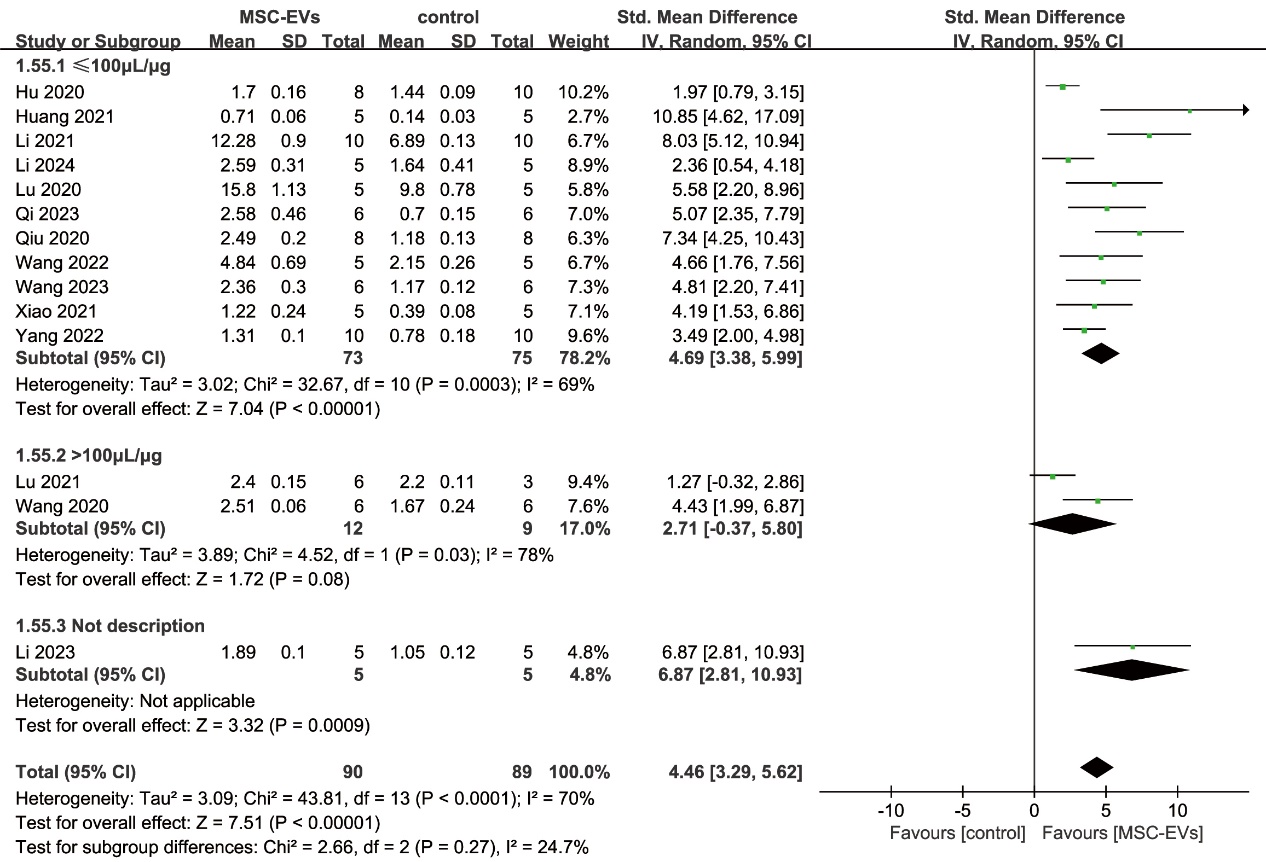


**Figure S24.** Subgroup analysis of Tb. N based on different MSC-EV intervention doses. Data are presented as standardized mean difference (SMD) with 95% confidence intervals (CI).


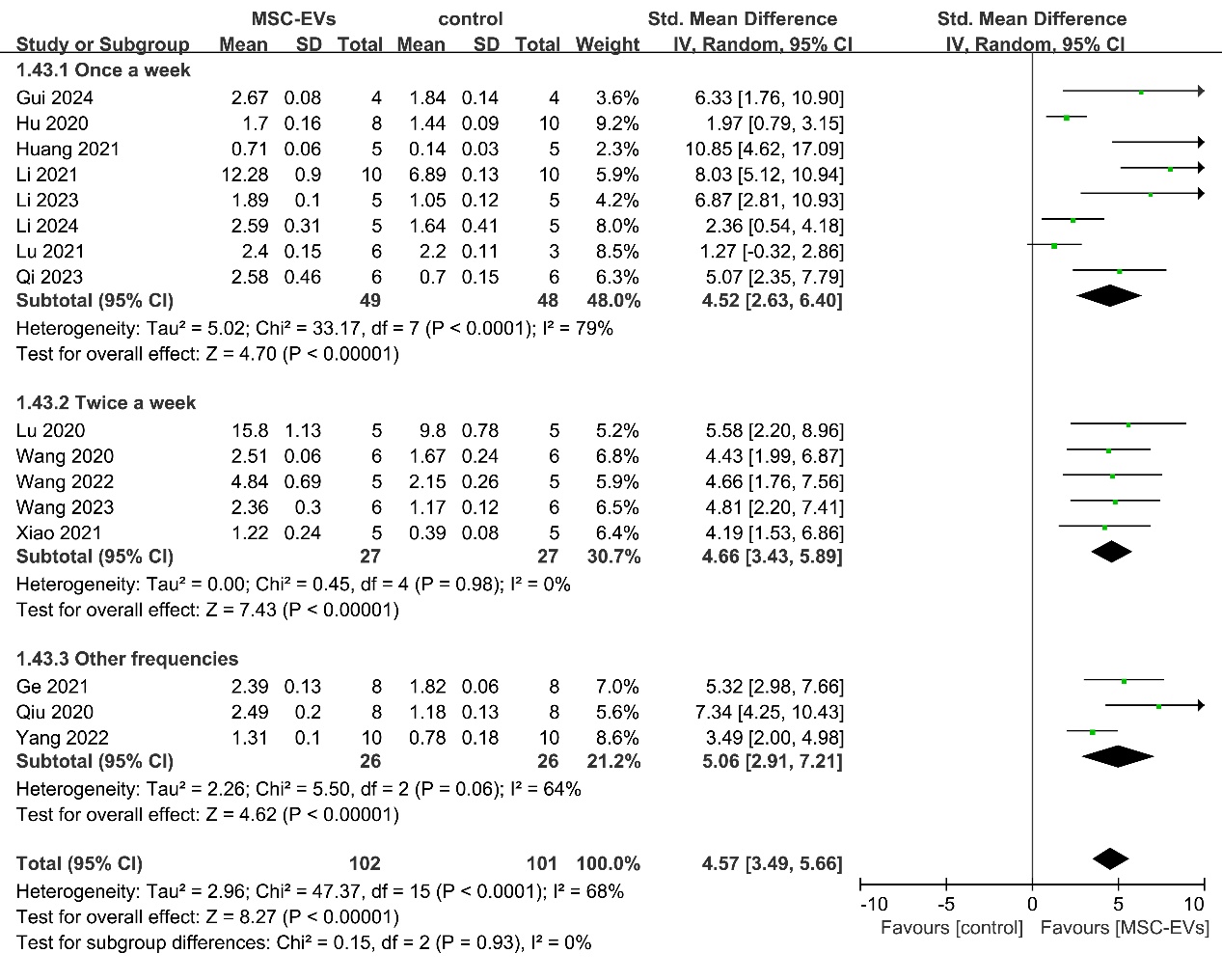
**Figure S25.** Subgroup analysis of Tb. N based on different administration frequencies. Data are presented as standardized mean difference (SMD) with 95% confidence intervals (CI).


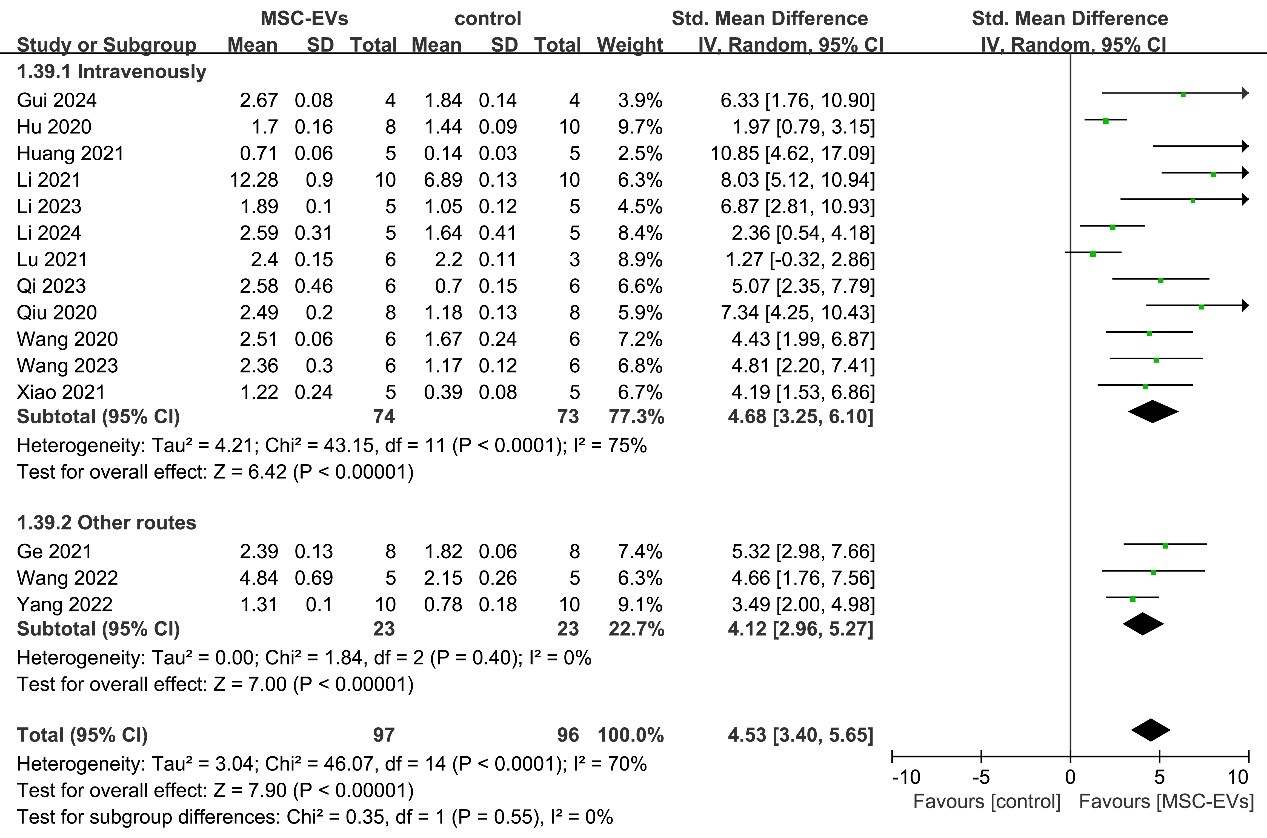


**Figure S26.** Subgroup analysis of Tb. N based on different administration routes. Data are presented as standardized mean difference (SMD) with 95% confidence intervals (CI).


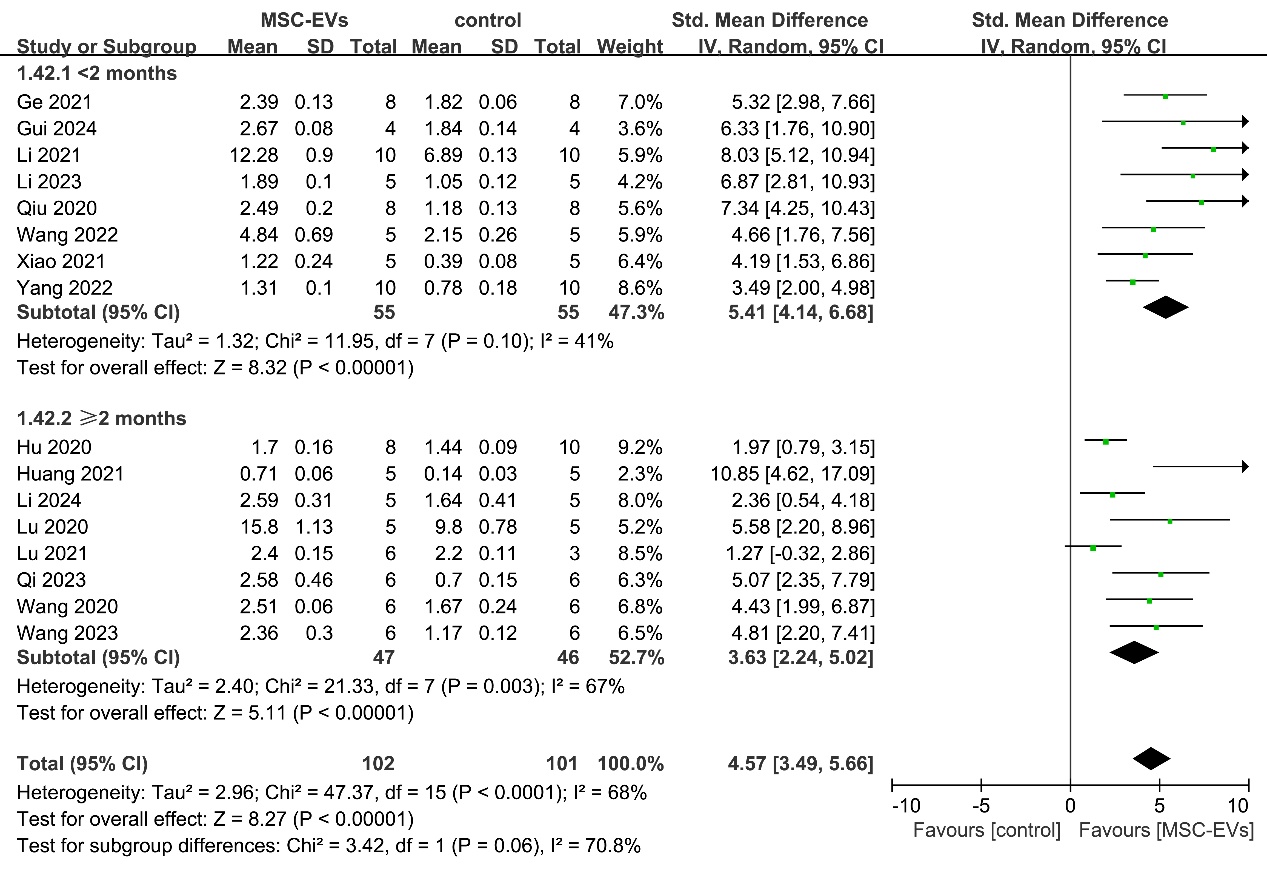


**Figure S27.** Subgroup analysis of Tb. N based on different treatment durations. Data are presented as standardized mean difference (SMD) with 95% confidence intervals (CI).


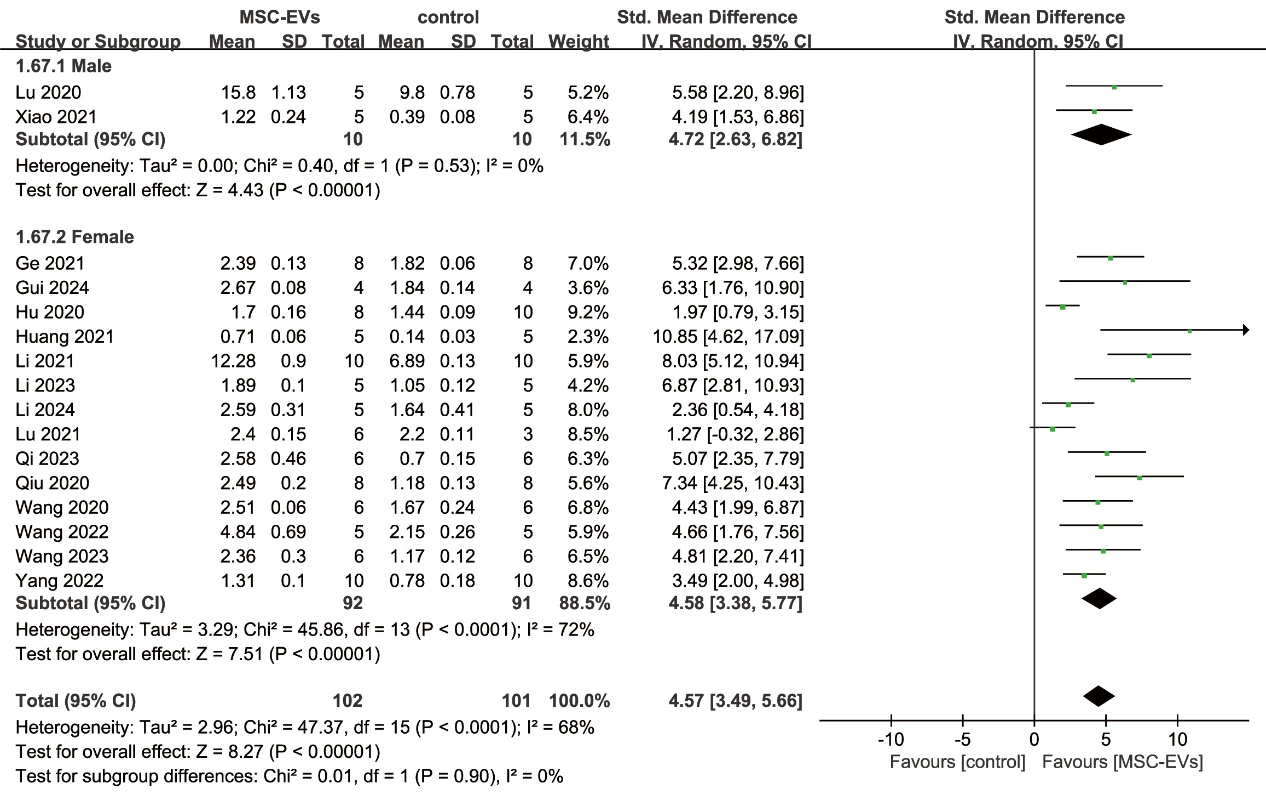


**Figure S28.** Subgroup analysis of Tb. N based on different animal sexes. Data are presented as standardized mean difference (SMD) with 95% confidence intervals (CI).


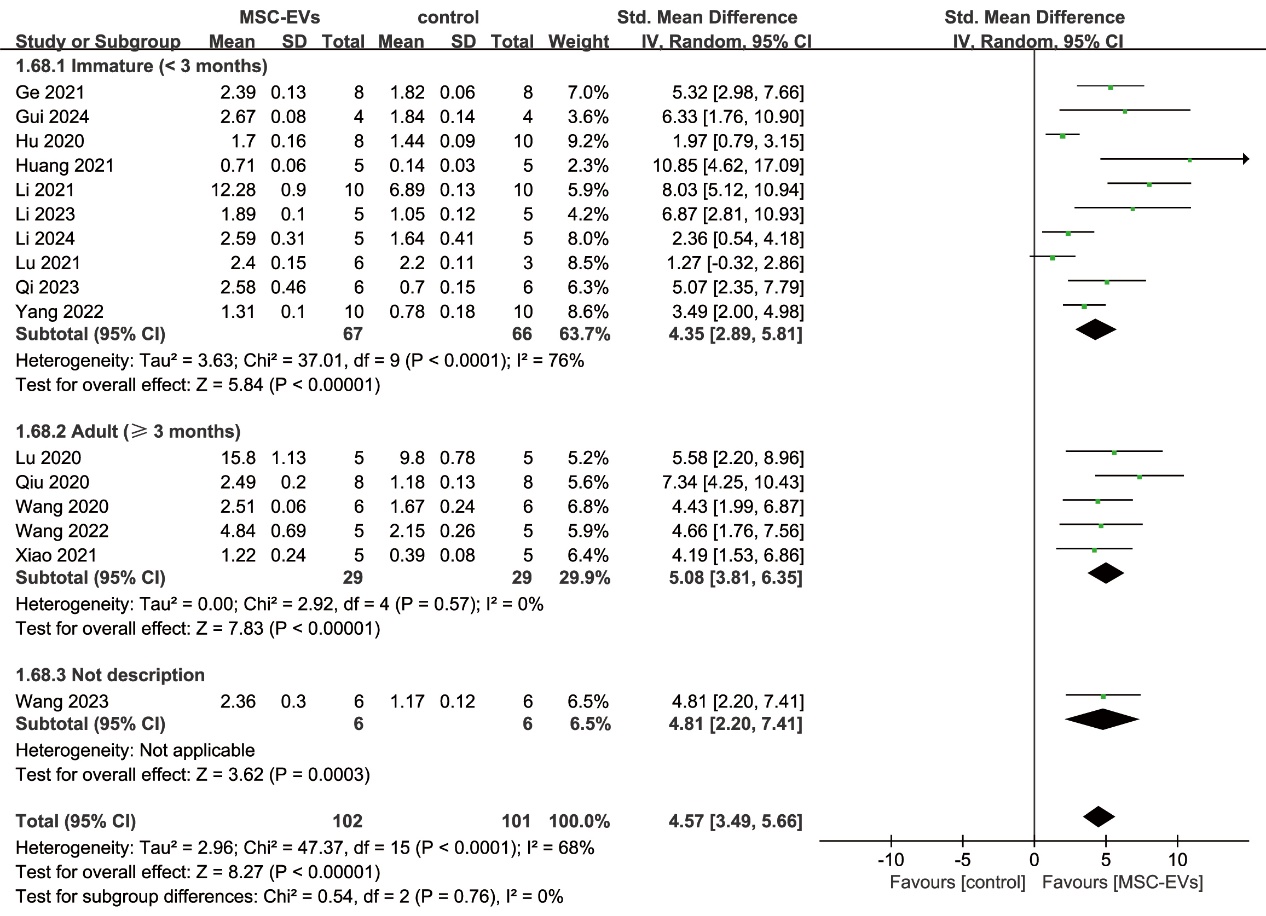


**Figure S29.** Subgroup analysis of Tb. N based on different animal ages. Data are presented as standardized mean difference (SMD) with 95% confidence intervals (CI).


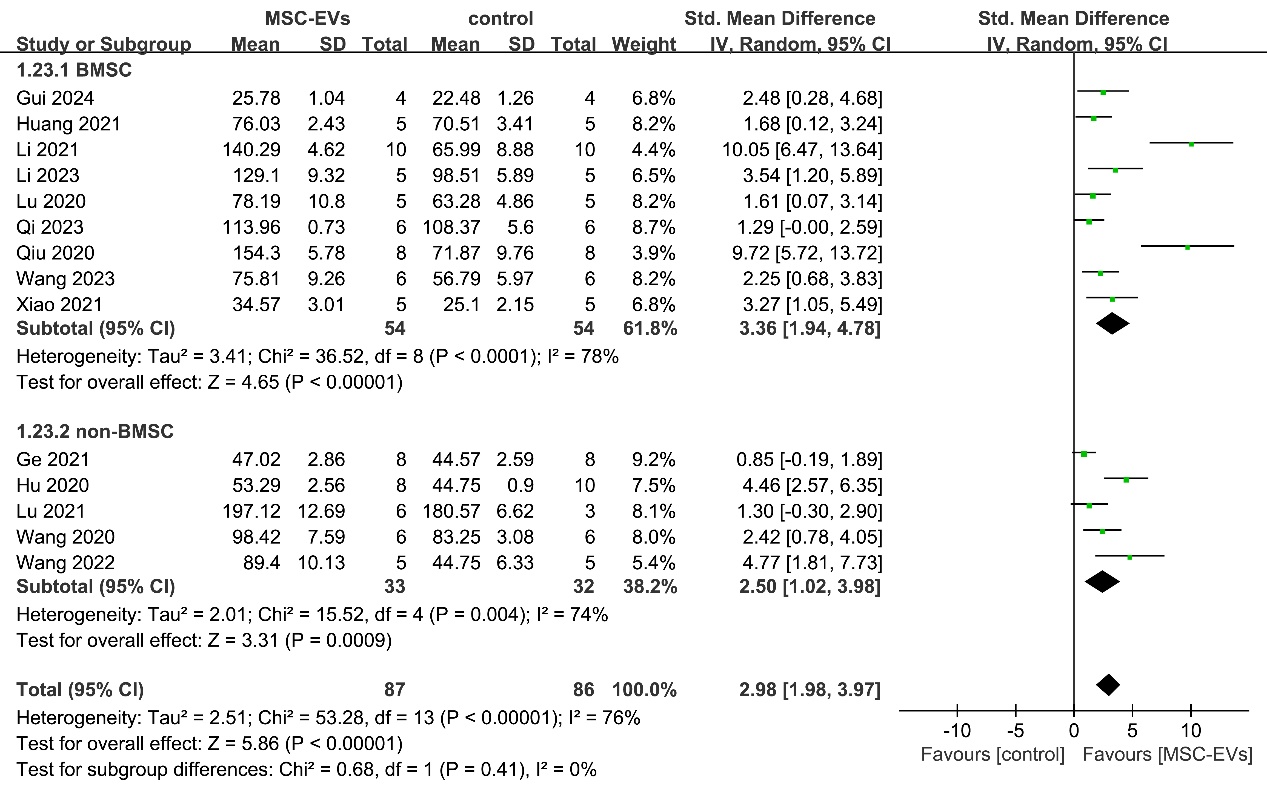


**Figure S30.** Subgroup analysis based on different MSC-EVs sources for Tb. Th. Data are presented as standardized mean difference (SMD) with 95% confidence intervals (CI).


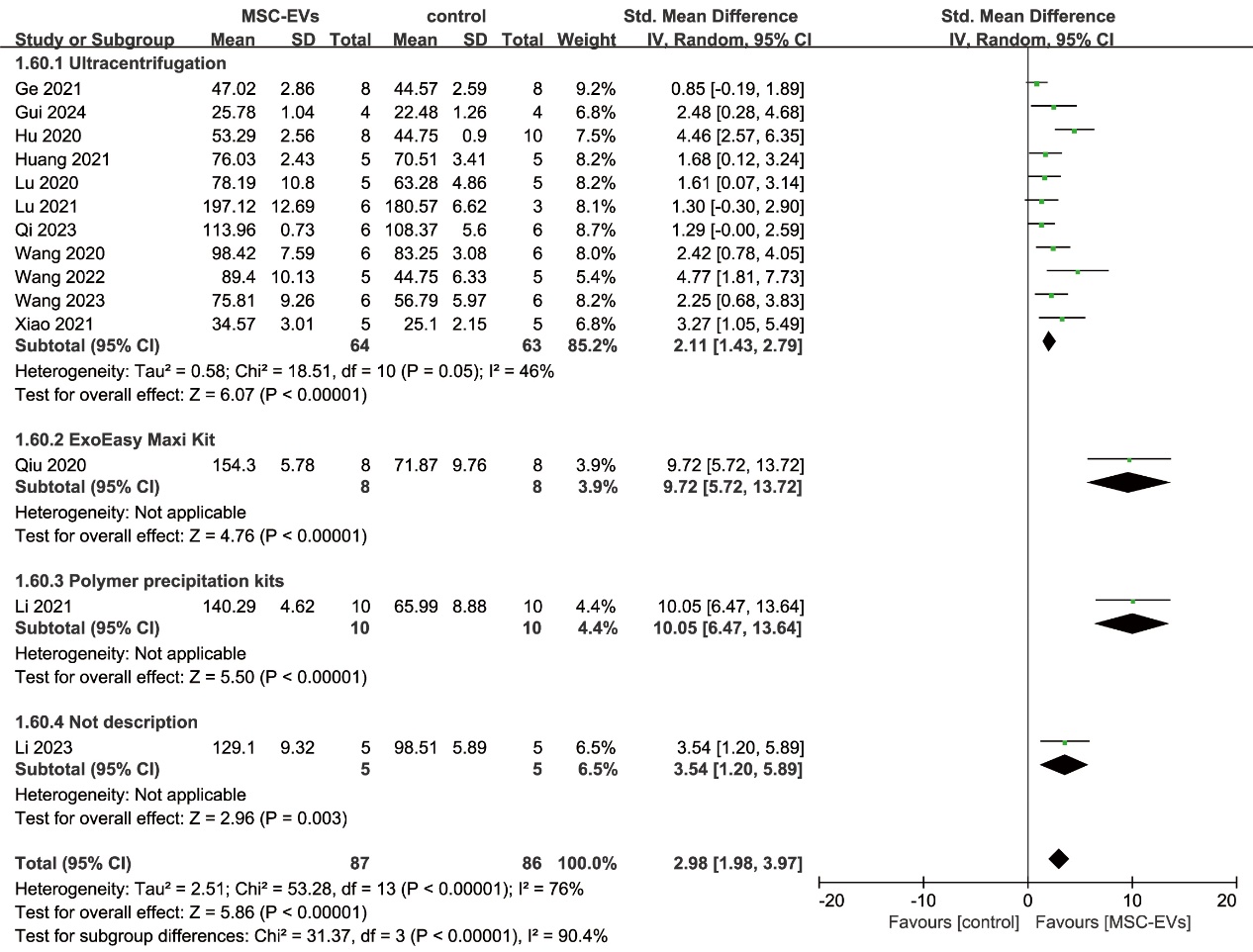


**Figure S31.** Subgroup analysis of Tb. Th based on different MSC-EVs isolation methods. Data are presented as standardized mean difference (SMD) with 95% confidence intervals (CI).

**
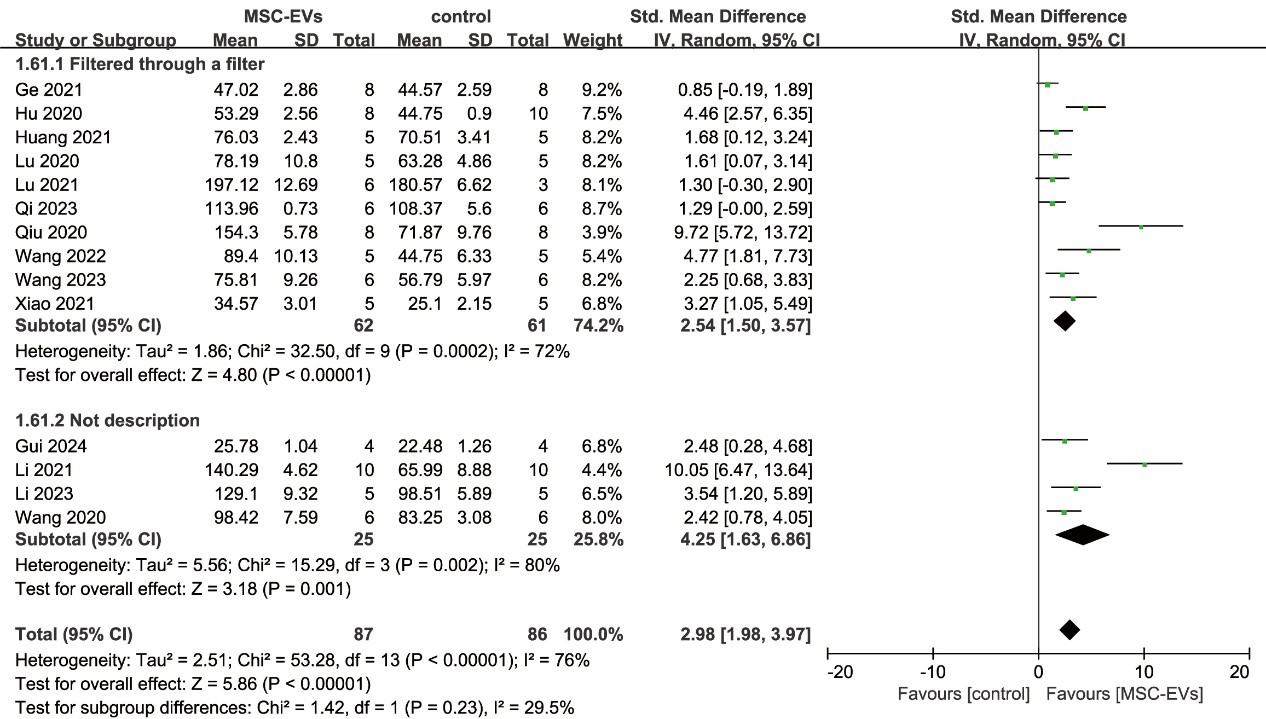
**

**Figure S32.** Subgroup analysis of Tb. Th based on different MSC-EVs purification methods. Data are presented as standardized mean difference (SMD) with 95% confidence intervals (CI).

**
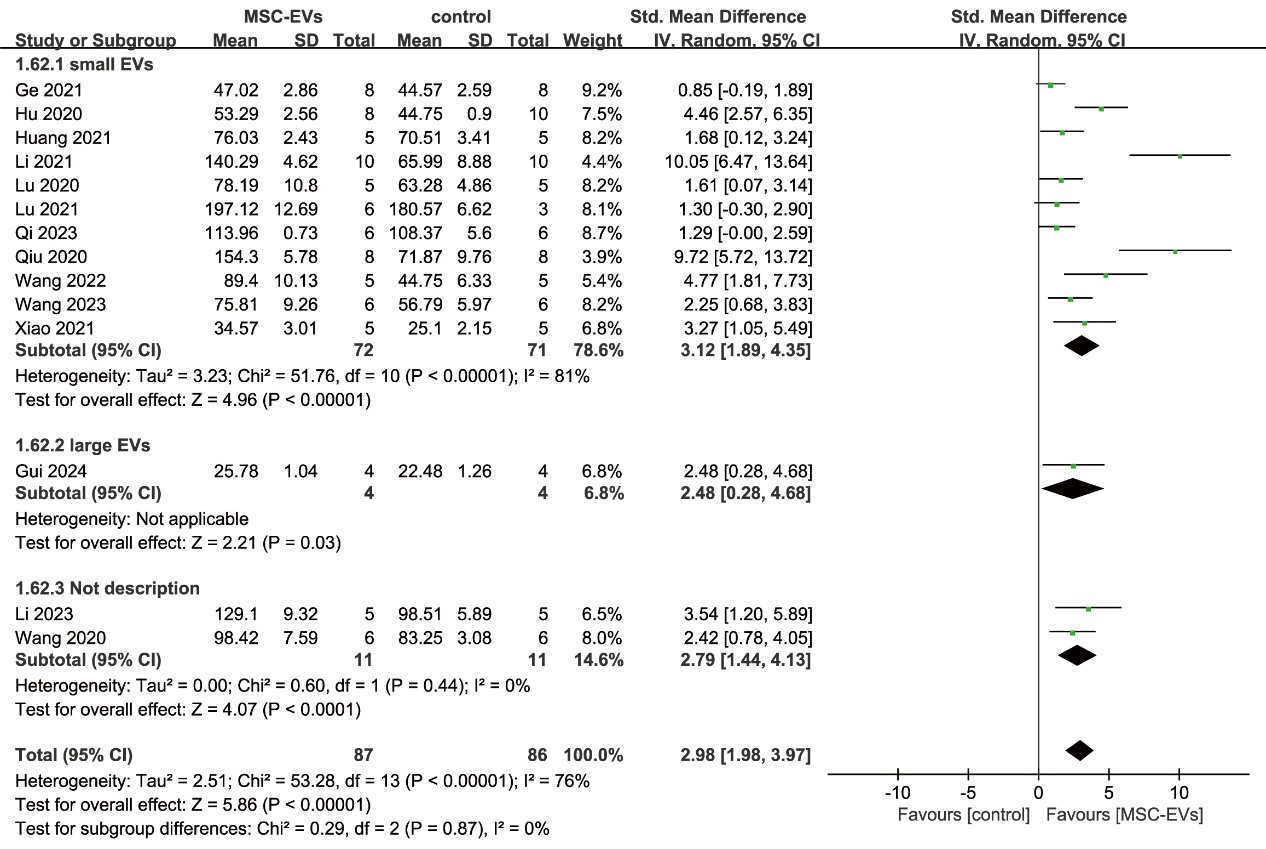
**

**Figure S33.** Subgroup analysis of Tb. Th based on different MSC-EV sizes. Data are presented as standardized mean difference (SMD) with 95% confidence intervals (CI).


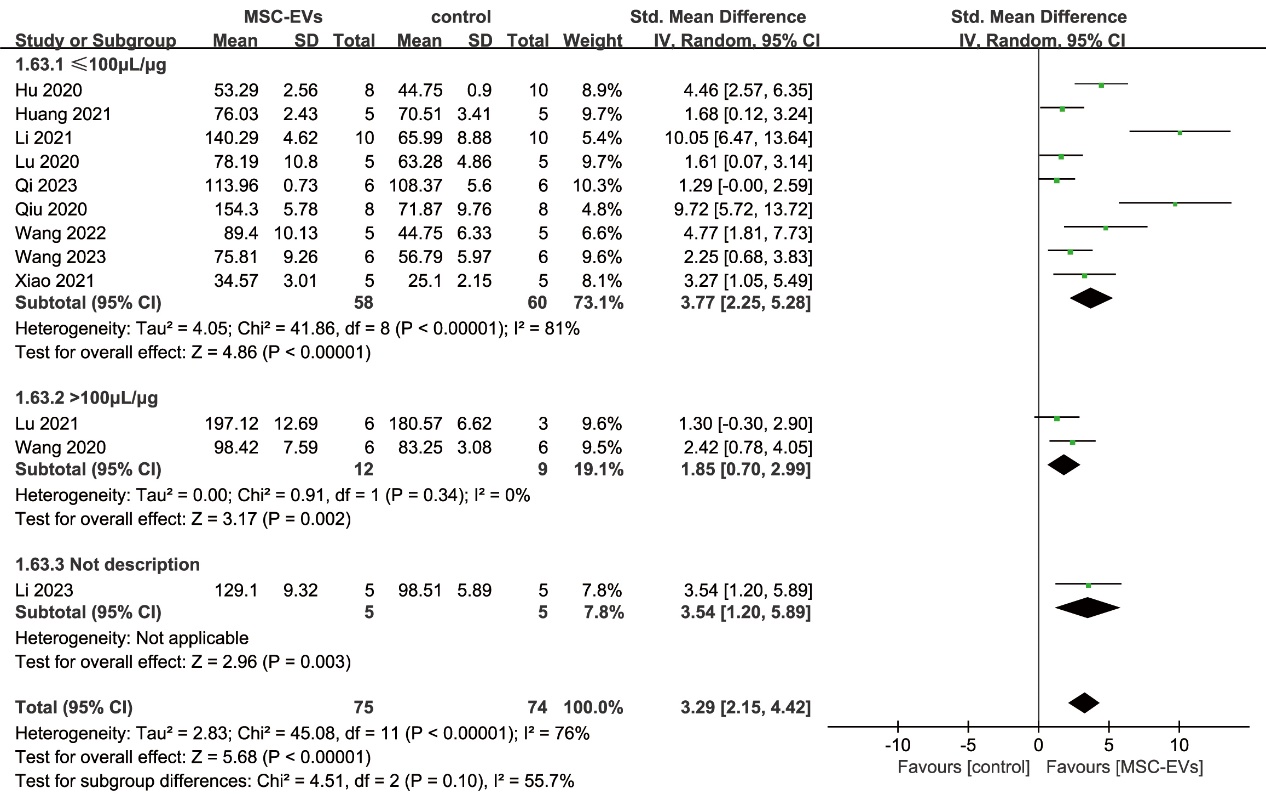


**Figure S34.** Subgroup analysis of Tb. Th based on different MSC-EV intervention doses. Data are presented as standardized mean difference (SMD) with 95% confidence intervals (CI).


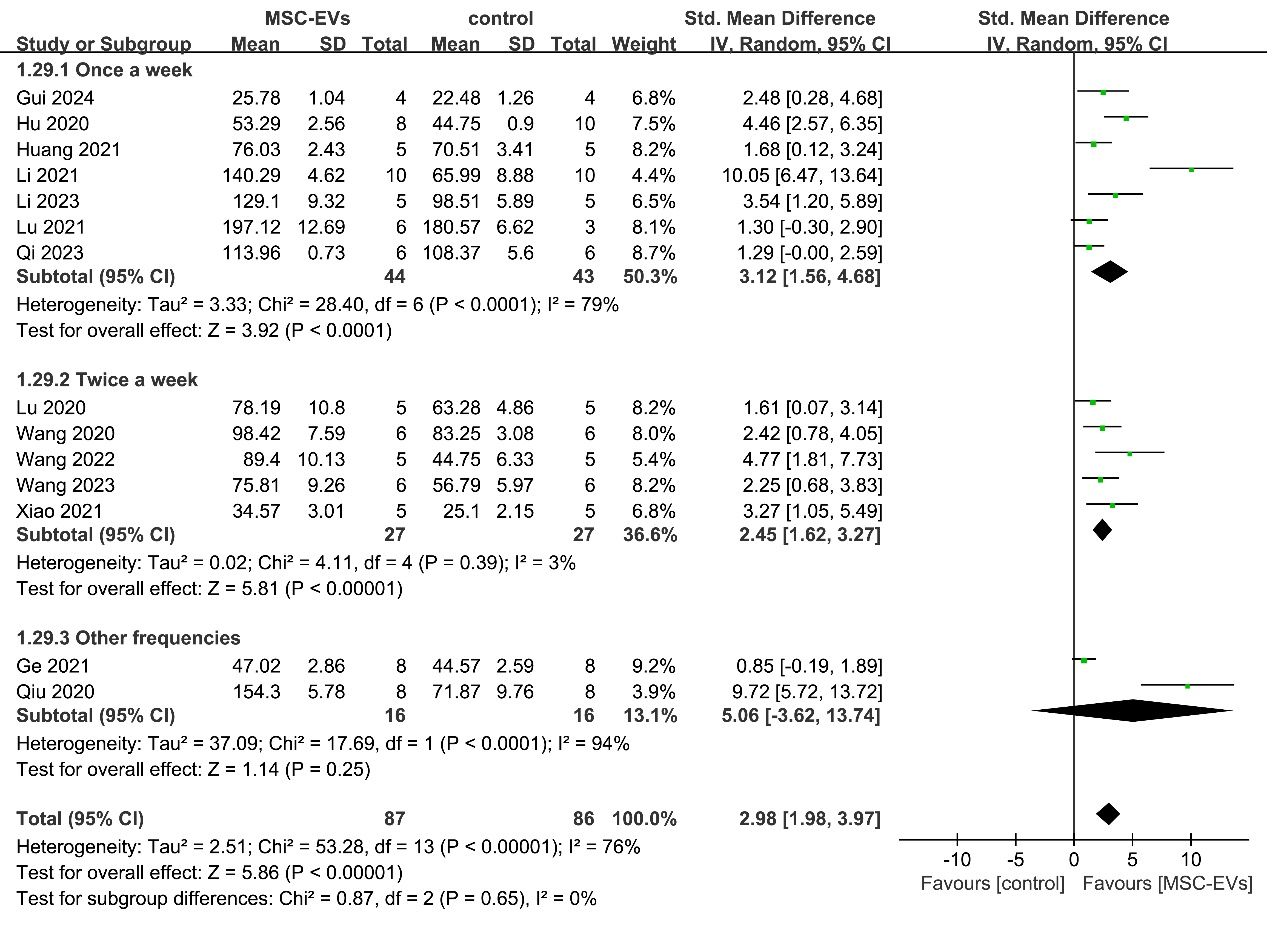


**Figure S35.** Subgroup analysis of Tb. Th based on different administration frequencies. Data are presented as standardized mean difference (SMD) with 95% confidence intervals (CI).


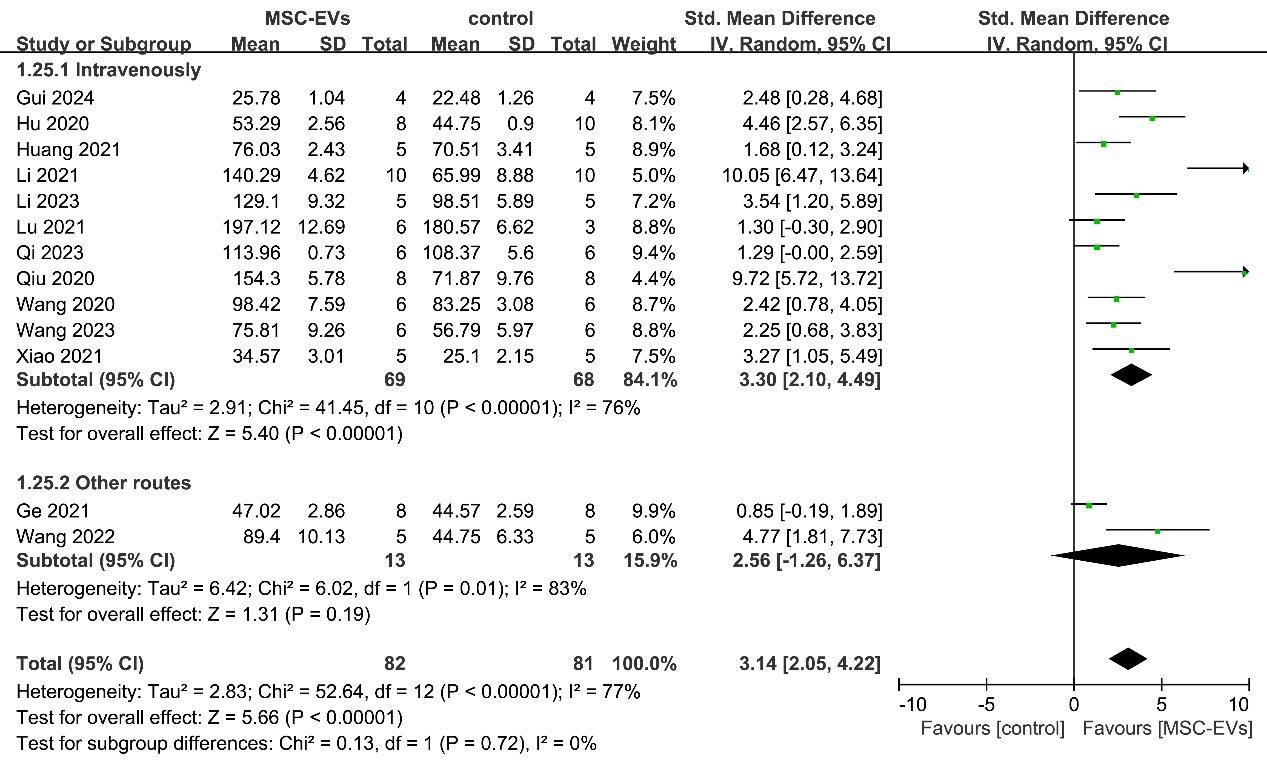


**Figure S36.** Subgroup analysis of Tb. Th based on different administration routes. Data are presented as standardized mean difference (SMD) with 95% confidence intervals (CI).


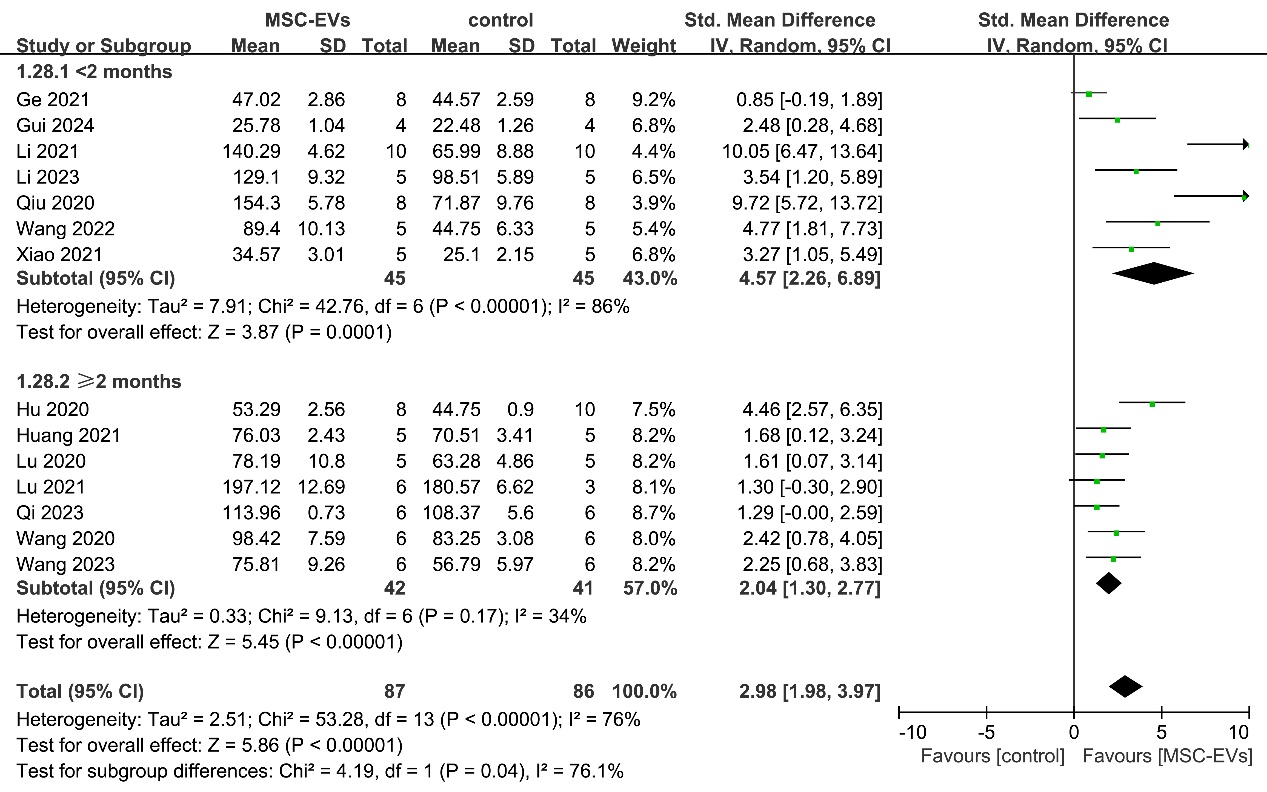


**Figure S37.** Subgroup analysis of Tb. Th based on different treatment durations. Data are presented as standardized mean difference (SMD) with 95% confidence intervals (CI).

**
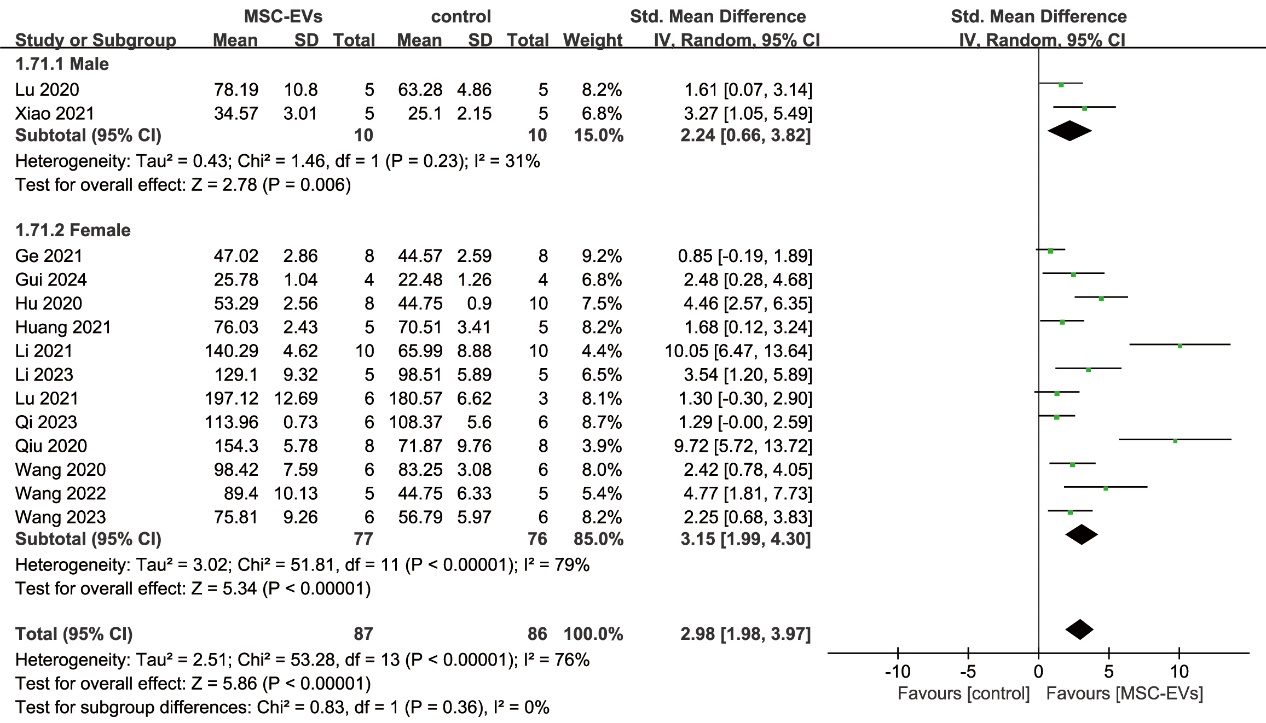
**

**Figure S38.** Subgroup analysis of Tb. Th based on different animal sexes. Data are presented as standardized mean difference (SMD) with 95% confidence intervals (CI).


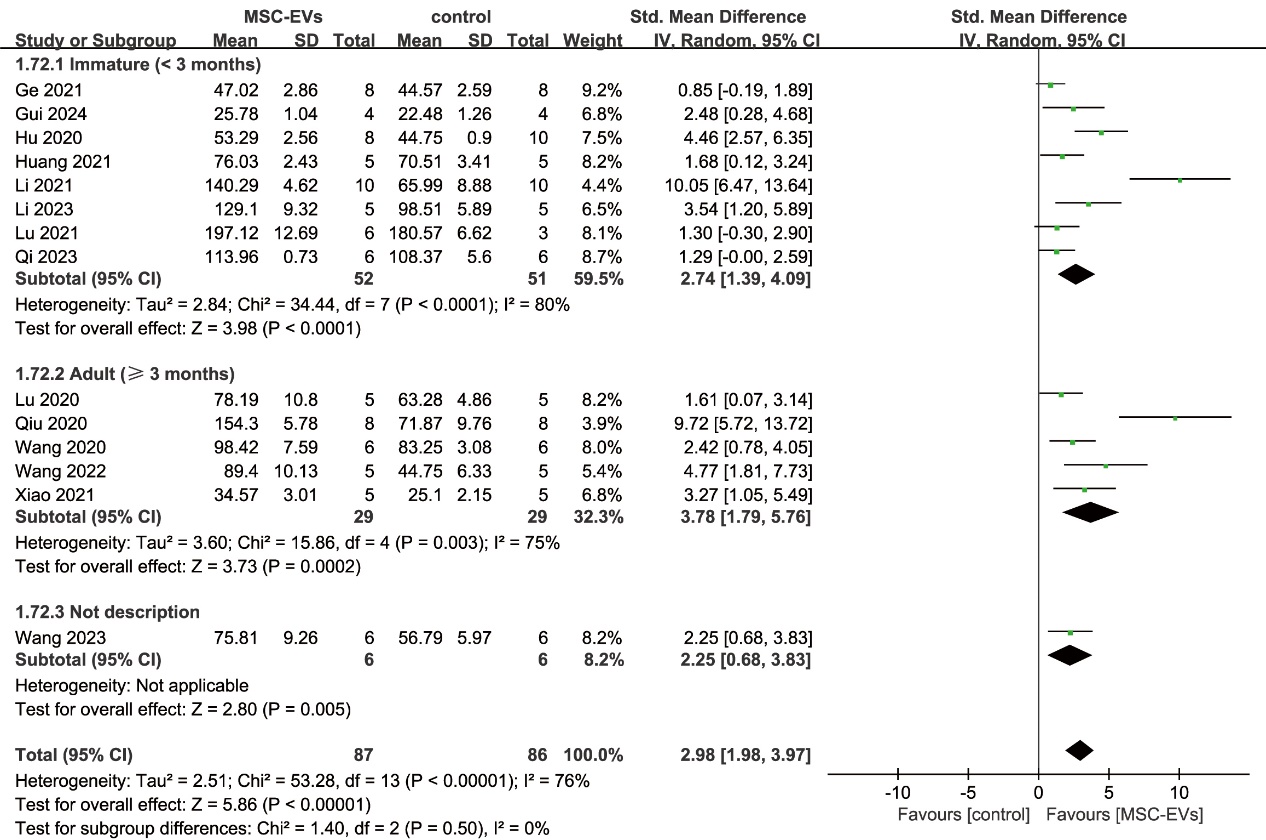


**Figure S39.** Subgroup analysis of Tb. Th based on different animal ages. Data are presented as standardized mean difference (SMD) with 95% confidence intervals (CI).


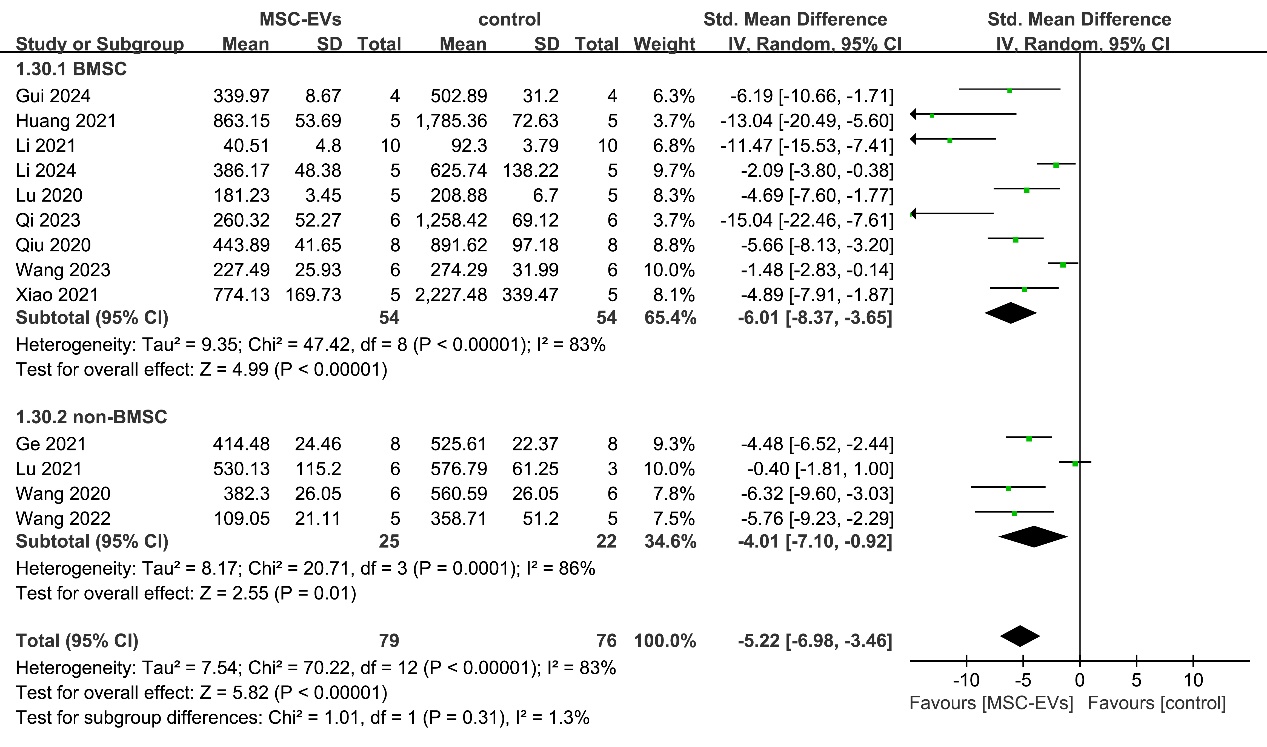


**Figure S40.** Subgroup analysis based on different MSC-EVs sources for Tb. Sp. Data are presented as standardized mean difference (SMD) with 95% confidence intervals (CI).


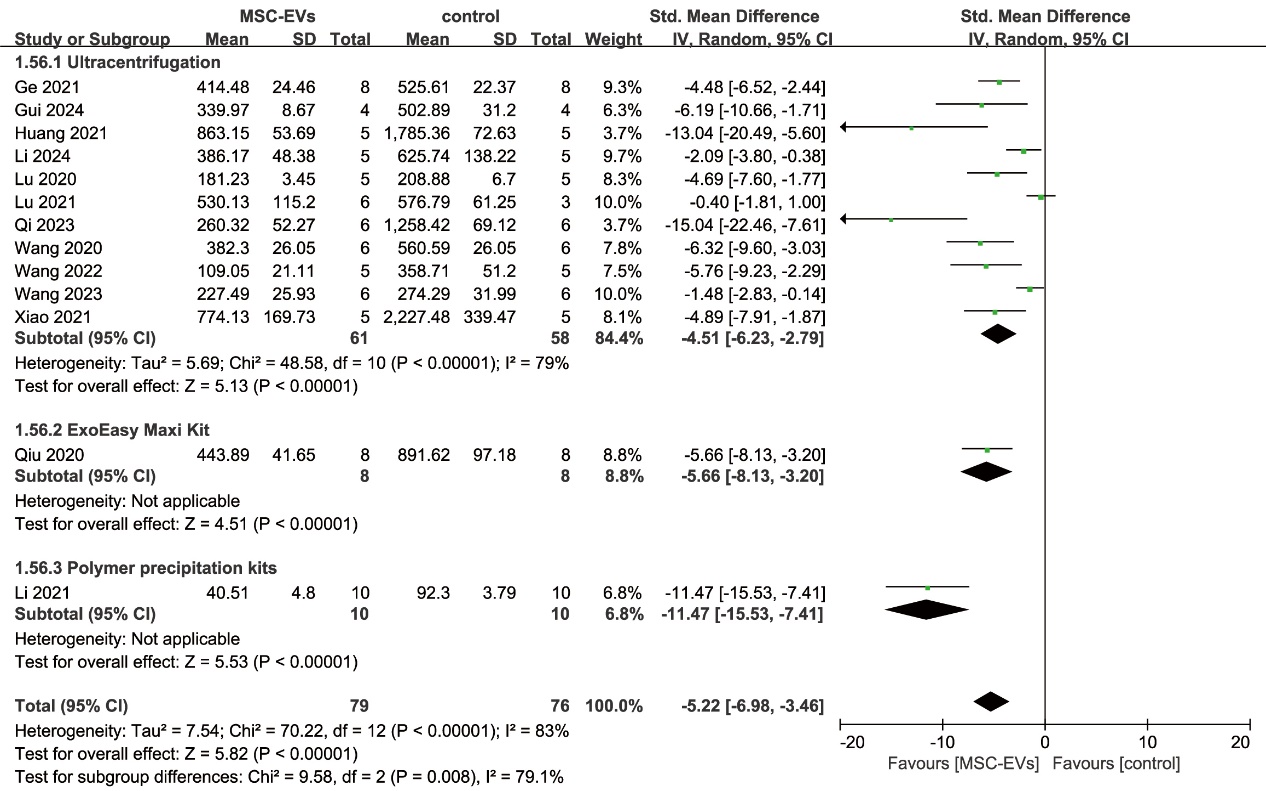


**Figure S41.** Subgroup analysis of Tb. Sp based on different MSC-EVs isolation methods. Data are presented as standardized mean difference (SMD) with 95% confidence intervals (CI).


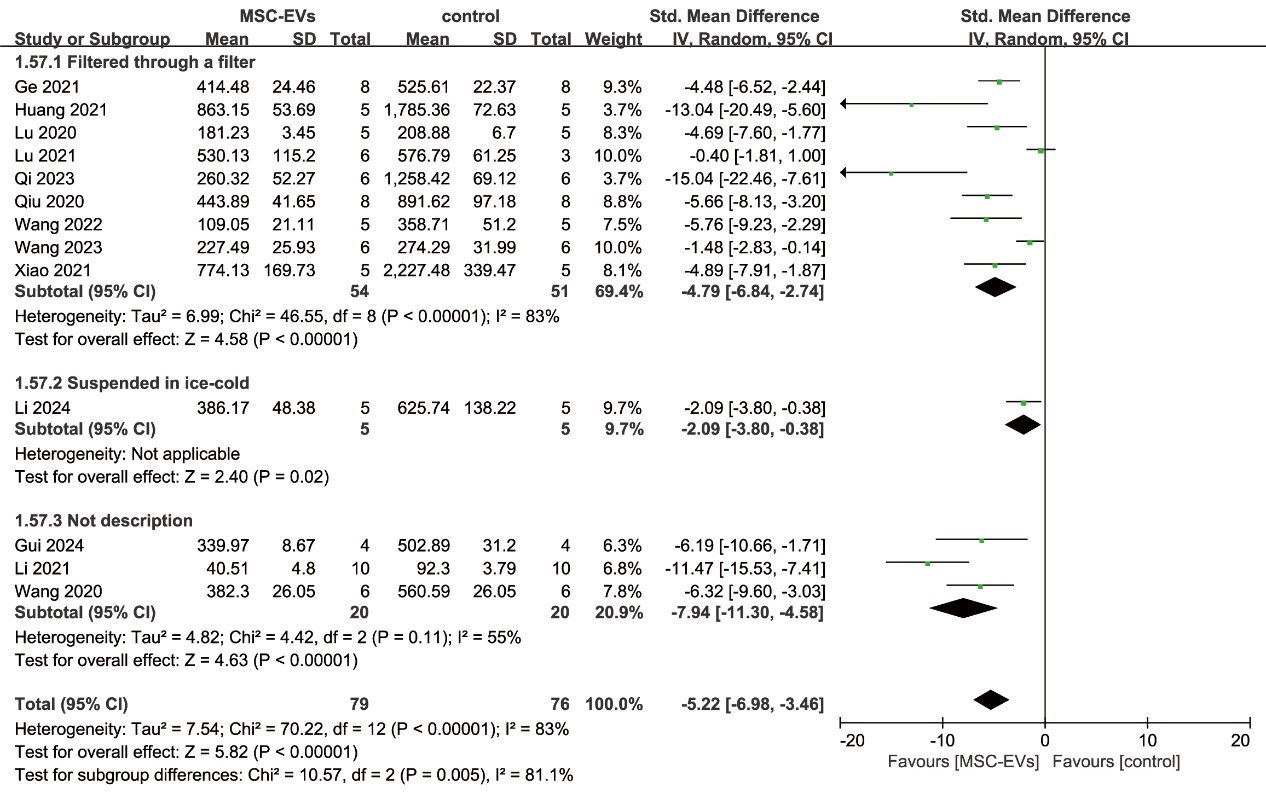


**Figure S42.** Subgroup analysis of Tb. Sp based on different MSC-EVs purification methods. Data are presented as standardized mean difference (SMD) with 95% confidence intervals (CI).


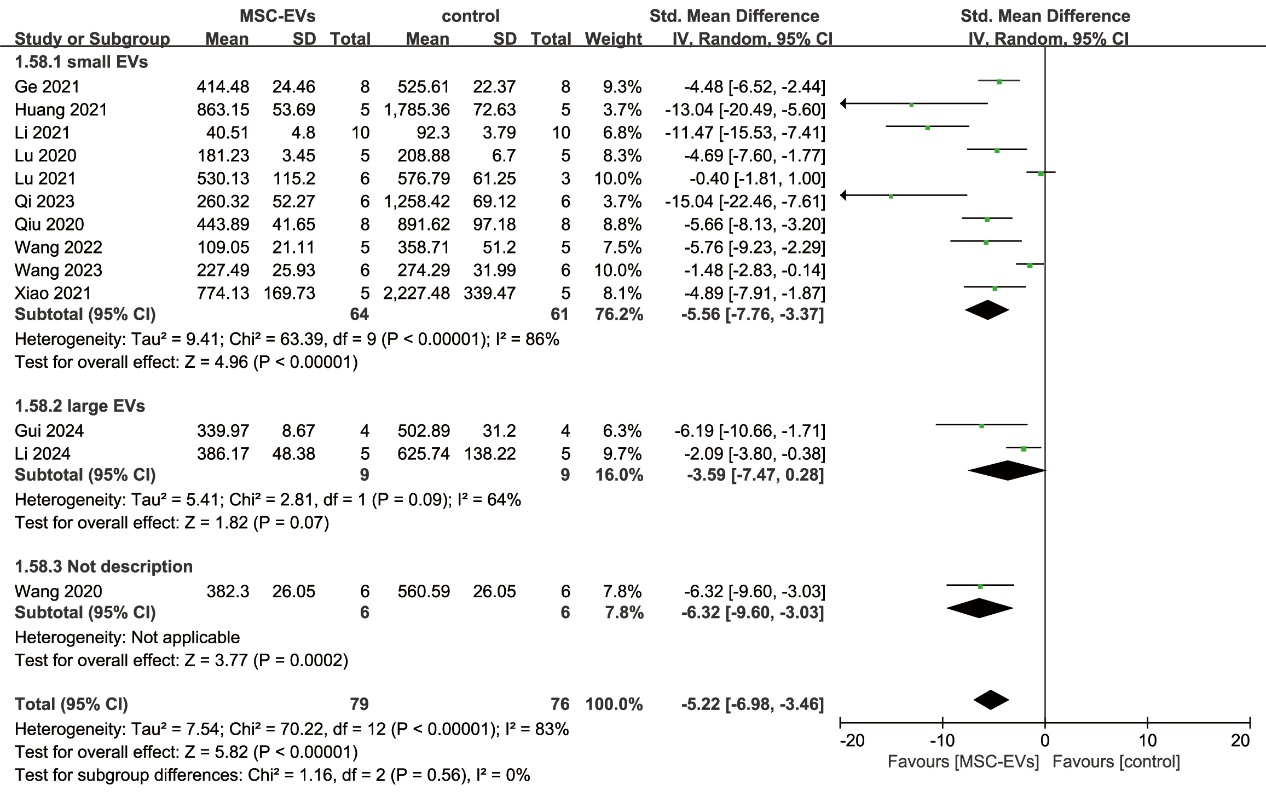


**Figure S43** Subgroup analysis of Tb. Sp based on different MSC-EV sizes. Data are presented as standardized mean difference (SMD) with 95% confidence intervals (CI).


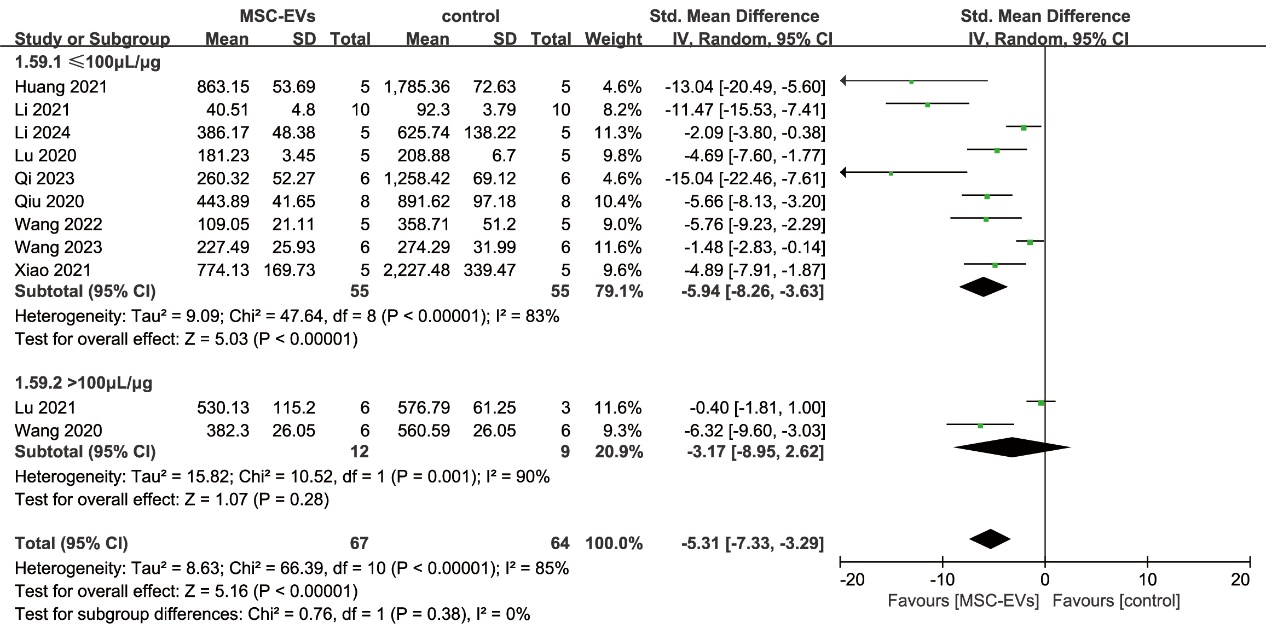


**Figure S44.** Subgroup analysis of Tb. Sp based on different MSC-EV intervention doses. Data are presented as standardized mean difference (SMD) with 95% confidence intervals (CI).


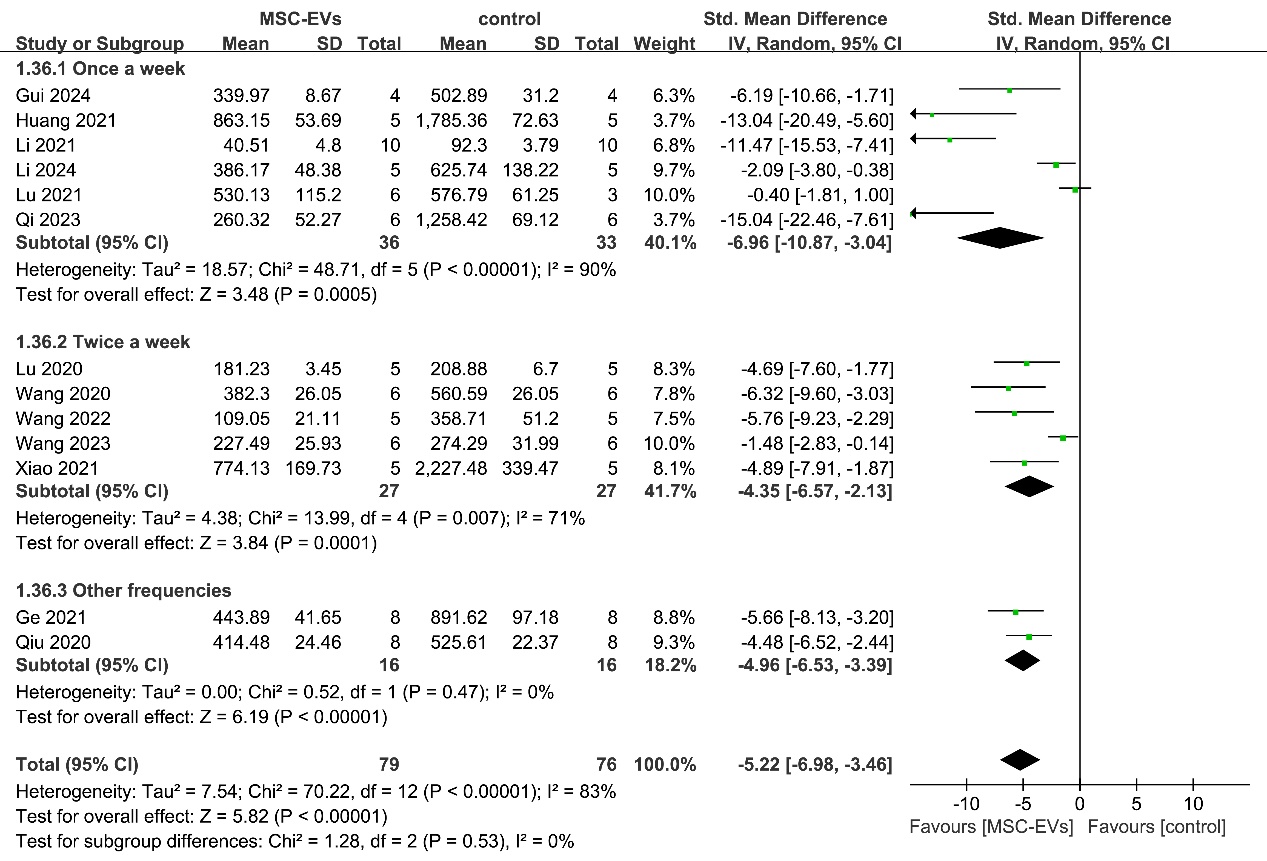


**Figure S45.** Subgroup analysis of Tb. Sp based on different administration frequencies. Data are presented as standardized mean difference (SMD) with 95% confidence intervals (CI).


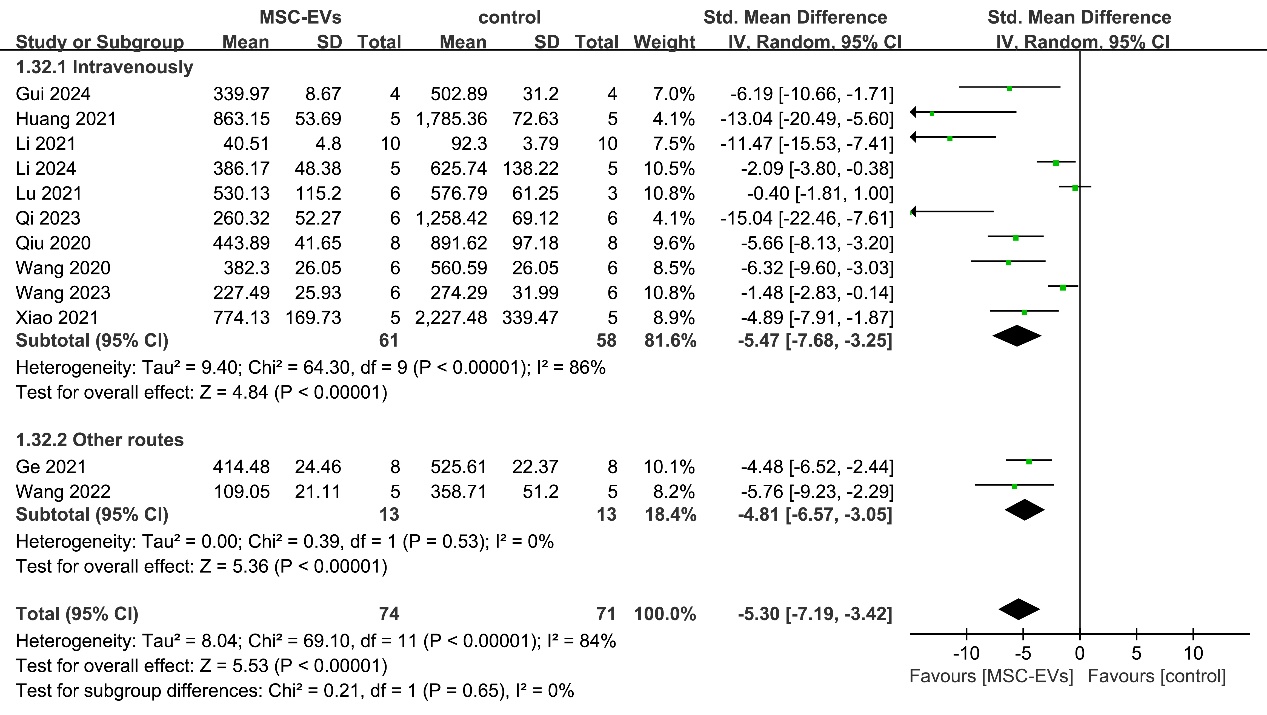


**Figure S46.** Subgroup analysis of Tb. Sp based on different administration routes. Data are presented as standardized mean difference (SMD) with 95% confidence intervals (CI).


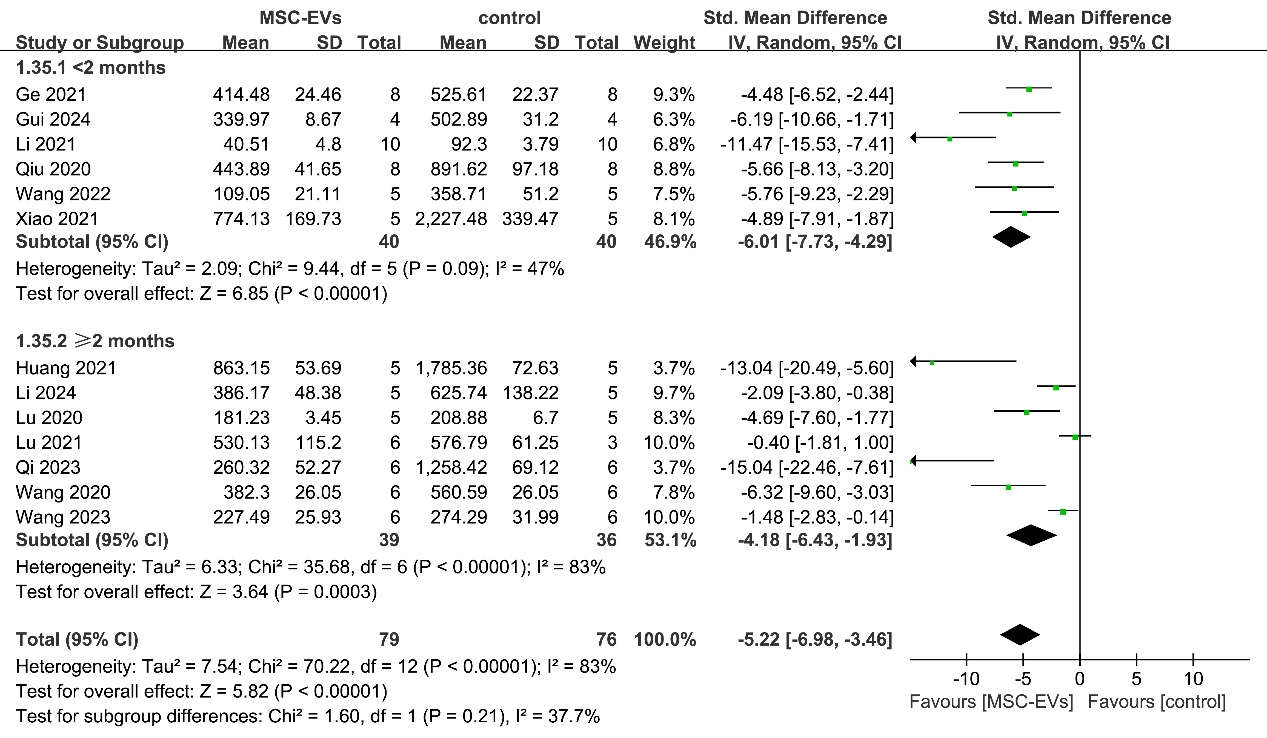


**Figure S47.** Subgroup analysis of Tb. Sp based on different treatment durations. Data are presented as standardized mean difference (SMD) with 95% confidence intervals (CI).


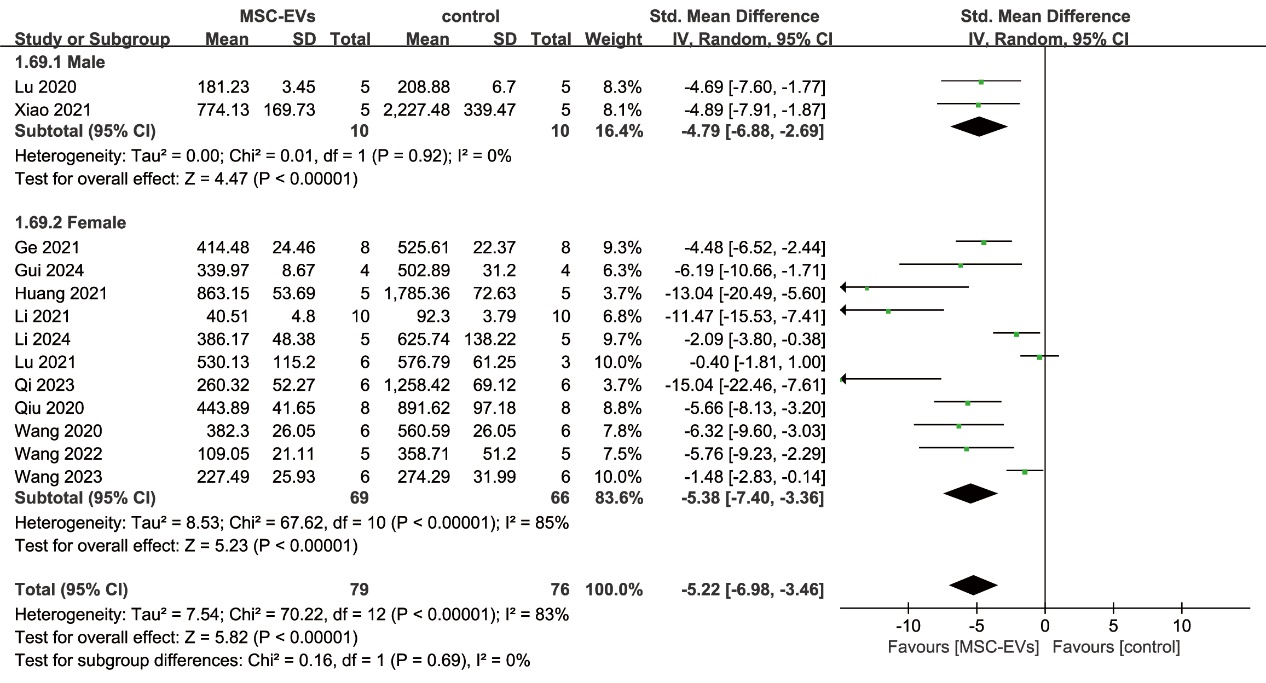


**Figure S48.** Subgroup analysis of Tb. Sp based on different animal sexes. Data are presented as standardized mean difference (SMD) with 95% confidence intervals (CI).


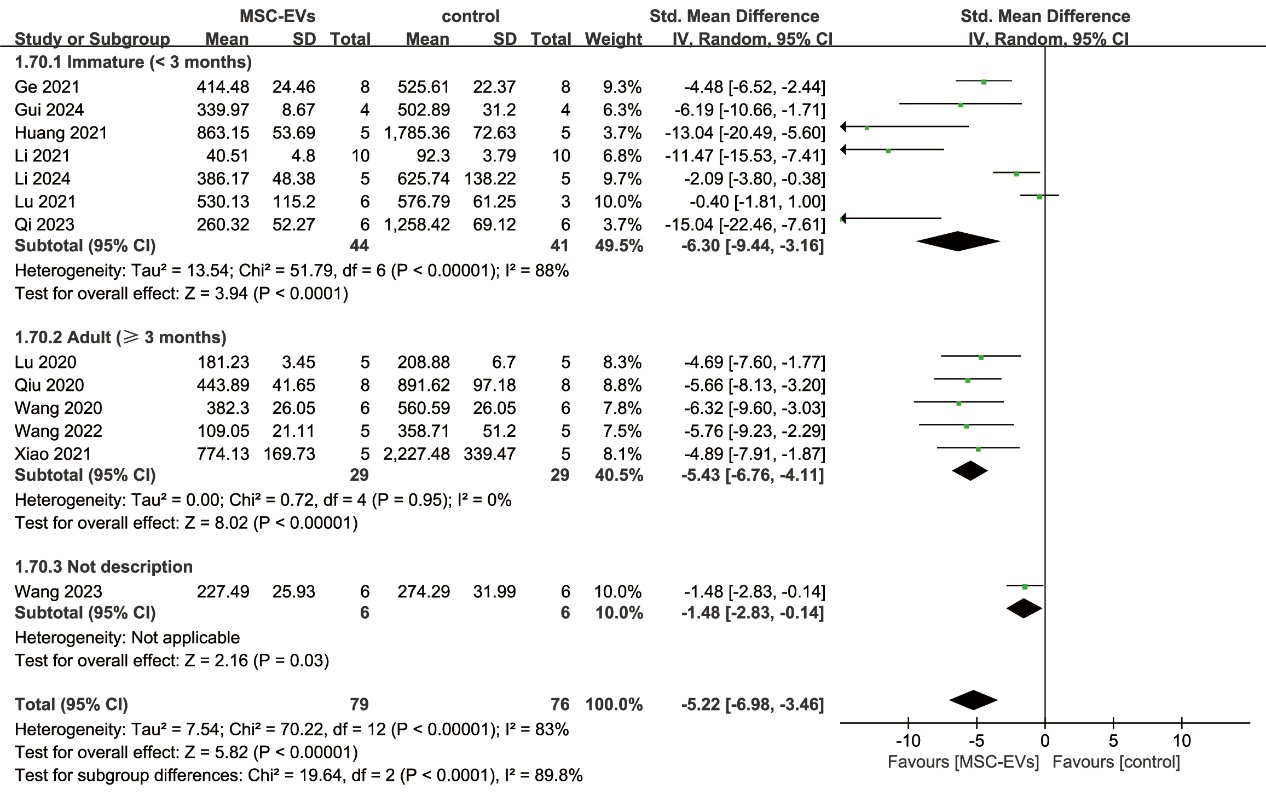


**Figure S49.** Subgroup analysis of Tb. Sp based on different animal ages. Data are presented as standardized mean difference (SMD) with 95% confidence intervals (CI).


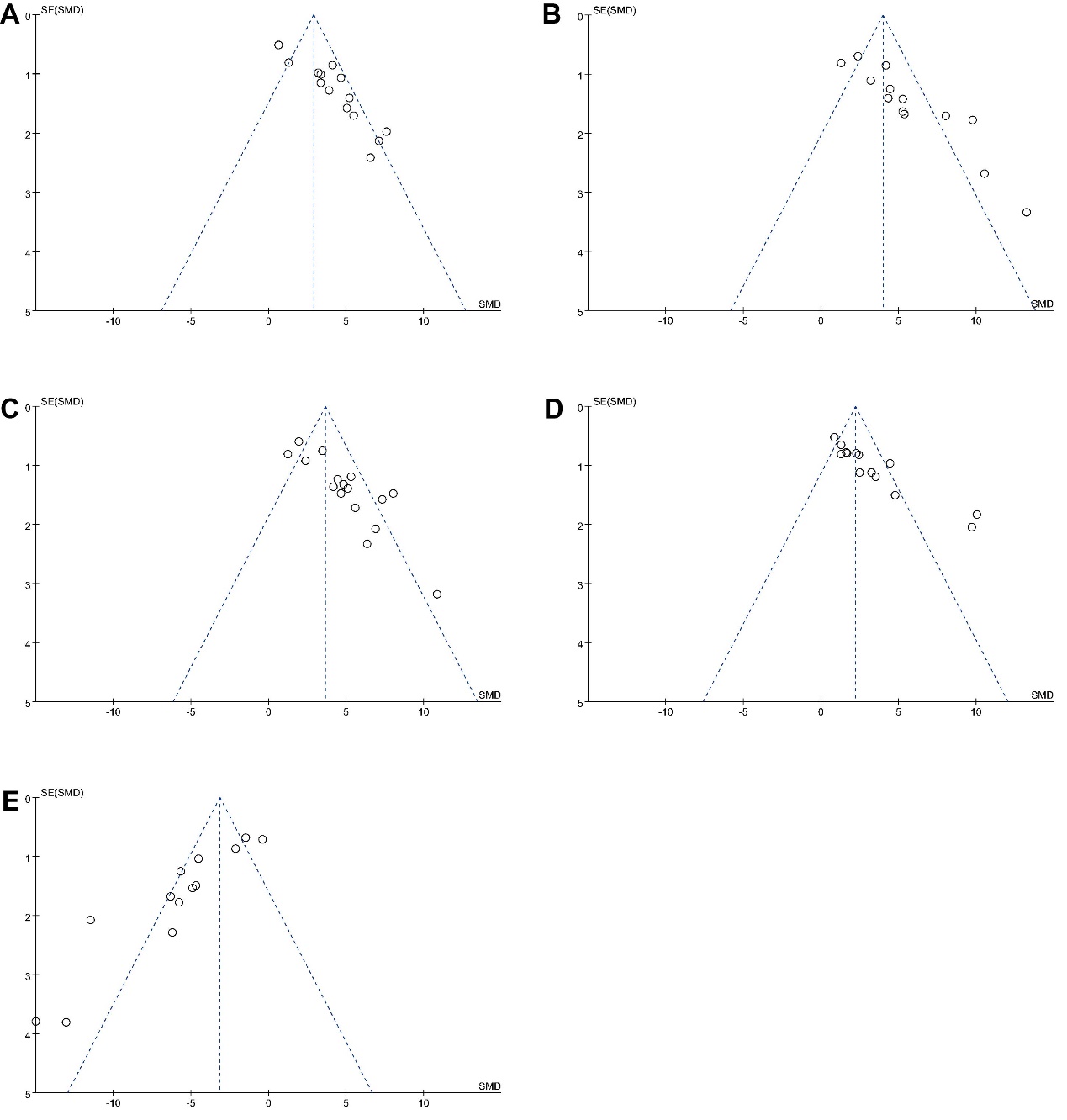


**Supplementary Figure 50.** Funnel plot illustrating the comparison between MSC-EVs and the control group. (A)BMD；(B)BV/TV；(C) Tb. Np；(D) Tb. Th；(E) Tb. Sp.


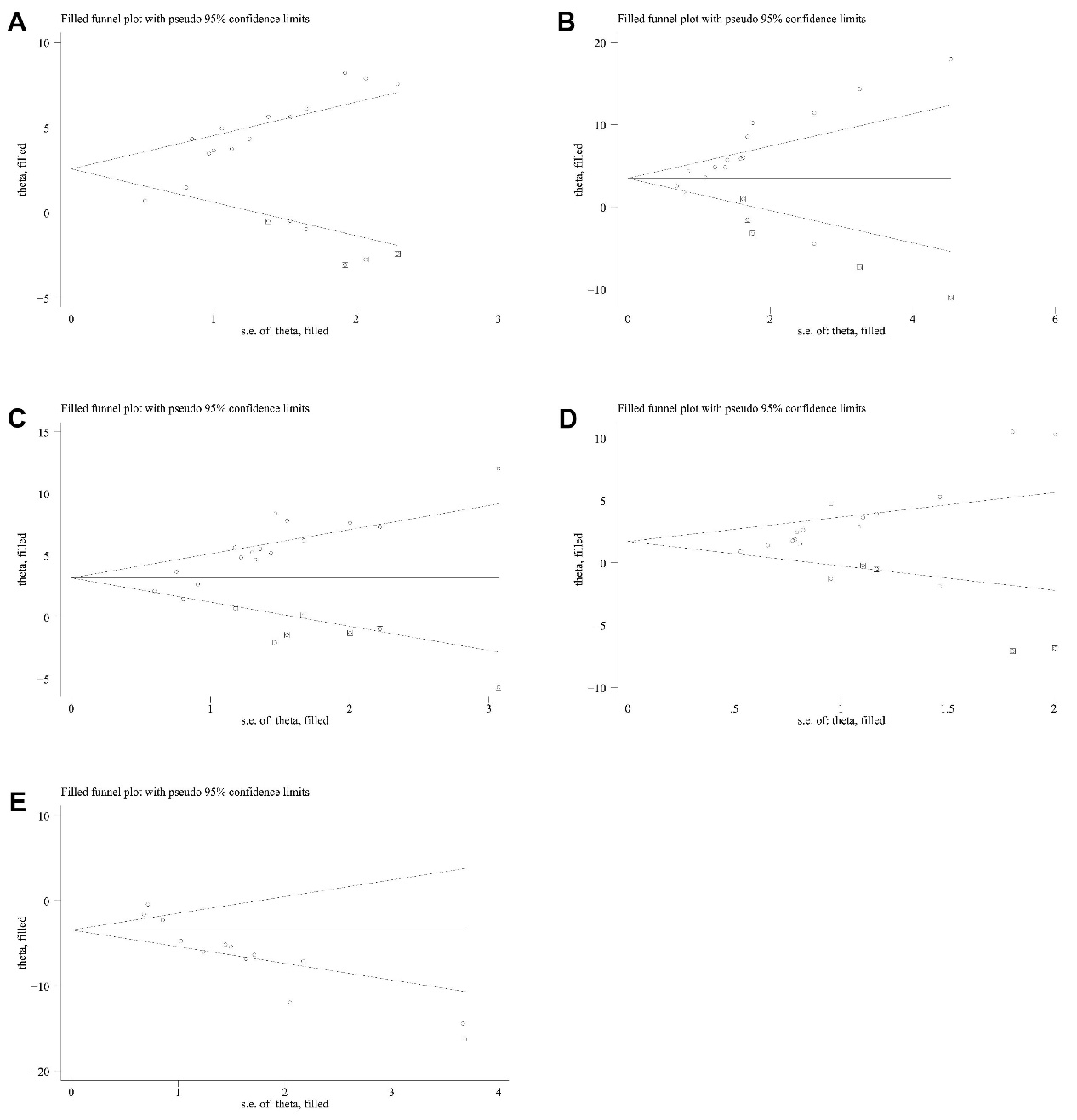


**Supplementary Figure 51.** Trim-and-fill analysis illustrating the comparison between MSC-EVs and the control group. (A)BMD；(B)BV/TV；(C) Tb. Np；(D) Tb. Th；(E) Tb. Sp.
